# A combined model of human erythropoiesis and granulopoiesis under growth factor and chemotherapy treatment

**DOI:** 10.1186/1742-4682-11-24

**Published:** 2014-05-26

**Authors:** Sibylle Schirm, Christoph Engel, Markus Loeffler, Markus Scholz

**Affiliations:** 1 Institute for Medical Informatics, Statistics and Epidemiology, University of Leipzig, Leipzig, Germany; 2 LIFE Research Center of Civilization Diseases, University of Leipzig, Leipzig, Germany

**Keywords:** Leucopenia, Anaemia, G-CSF, EPO, Filgrastim, Pegfilgrastim, Darbepoetin

## Abstract

**Background:**

Haematotoxicity of conventional chemotherapies often results in delays of treatment or reduction of chemotherapy dose. To ameliorate these side-effects, patients are routinely treated with blood transfusions or haematopoietic growth factors such as erythropoietin (EPO) or granulocyte colony-stimulating factor (G-CSF). For the latter ones, pharmaceutical derivatives are available, which differ in absorption kinetics, pharmacokinetic and -dynamic properties. Due to the complex interaction of cytotoxic effects of chemotherapy and the stimulating effects of different growth factor derivatives, optimal treatment is a non-trivial task. In the past, we developed mathematical models of thrombopoiesis, granulopoiesis and erythropoiesis under chemotherapy and growth-factor applications which can be used to perform clinically relevant predictions regarding the feasibility of chemotherapy schedules and cytopenia prophylaxis with haematopoietic growth factors. However, interactions of lineages and growth-factors were ignored so far.

**Results:**

To close this gap, we constructed a hybrid model of human granulopoiesis and erythropoiesis under conventional chemotherapy, G-CSF and EPO applications. This was achieved by combining our single lineage models of human erythropoiesis and granulopoiesis with a common stem cell model. G-CSF effects on erythropoiesis were also implemented. Pharmacodynamic models are based on ordinary differential equations describing proliferation and maturation of haematopoietic cells. The system is regulated by feedback loops partly mediated by endogenous and exogenous EPO and G-CSF. Chemotherapy is modelled by depletion of cells. Unknown model parameters were determined by fitting the model predictions to time series data of blood counts and cytokine profiles. Data were extracted from literature or received from cooperating clinical study groups. Our model explains dynamics of mature blood cells and cytokines after growth-factor applications in healthy volunteers. Moreover, we modelled 15 different chemotherapeutic drugs by estimating their bone marrow toxicity. Taking into account different growth-factor schedules, this adds up to 33 different chemotherapy regimens explained by the model.

**Conclusions:**

We conclude that we established a comprehensive biomathematical model to explain the dynamics of granulopoiesis and erythropoiesis under combined chemotherapy, G-CSF, and EPO applications. We demonstrate how it can be used to make predictions regarding haematotoxicity of yet untested chemotherapy and growth-factor schedules.

## Background

Haematotoxicity of conventional multidrug, multi-cycle chemotherapies often results in delays of treatment or reduction of chemotherapy dose [1]. To ameliorate these side-effects, patients are routinely treated with blood transfusions (erythrocyte, platelet concentrates) or haematopoietic growth factors. For the latter ones, highly potential pharmaceutical derivatives are available, which however differ in their pharmacokinetic and -dynamic (PK/PD) properties. Due to the complex interaction of cytotoxic effects of chemotherapy and the stimulating effects of different growth factors, optimal treatment is a non-trivial task.

In the past, we developed mathematical models of thrombopoiesis (formation of platelets responsible for coagulation), granulopoiesis (formation of neutrophil granulocytes responsible for unspecific immune defense) and erythropoiesis (formation of red blood cells responsible for oxygen supply) under chemotherapy and growth-factor applications which can be used to perform clinically relevant predictions regarding feasibility of chemotherapy schedules and cytopenia prophylaxis with haematopoietic growth factors [2-4]. A few predictions were tested and validated in subsequent clinical trials, resulting in improved granulocyte colony-stimulating factor (G-CSF) schedules [5,6]. However, interactions of lineages and growth-factors were ignored so far.

In this paper, we aim to combine our models of erythropoiesis and granulopoiesis by constructing a model of both cell lines under chemotherapy, G-CSF and erythropoietin (EPO) applications. Rather than constructing a model from scratch we rely on the above mentioned established models to perform a second step towards a more comprehensive model. This implies that we kept the major model assumptions, equations and parameters of the isolated erythropoiesis and granulopoiesis model as far as possible.

Our goal is to create a hybrid model of human granulopoiesis and erythropoiesis usable for simulations of various chemotherapy schedules with EPO and G-CSF support taking into account interactions of the lineages via G-CSF effects on erythropoiesis [7-9]. Combining the models at stem cell level is a challenging issue, since the regulation of the stem cell compartment differs between the single lineage models.

In the present paper, we describe adaptations necessary to combine the models in detail. We compare the new hybrid model with the single lineage models for a number of scenarios such as cell loss or single injections of growth-factors. We also validate the hybrid model on the basis of a large number of data sets obtained from phase I, II and III clinical trials containing growth-factor or chemotherapy applications or combinations of it. Finally, we perform predictions regarding combined EPO and G-CSF applications during chemotherapy.

## Methods

### General structure of the model

**Figure 1:**
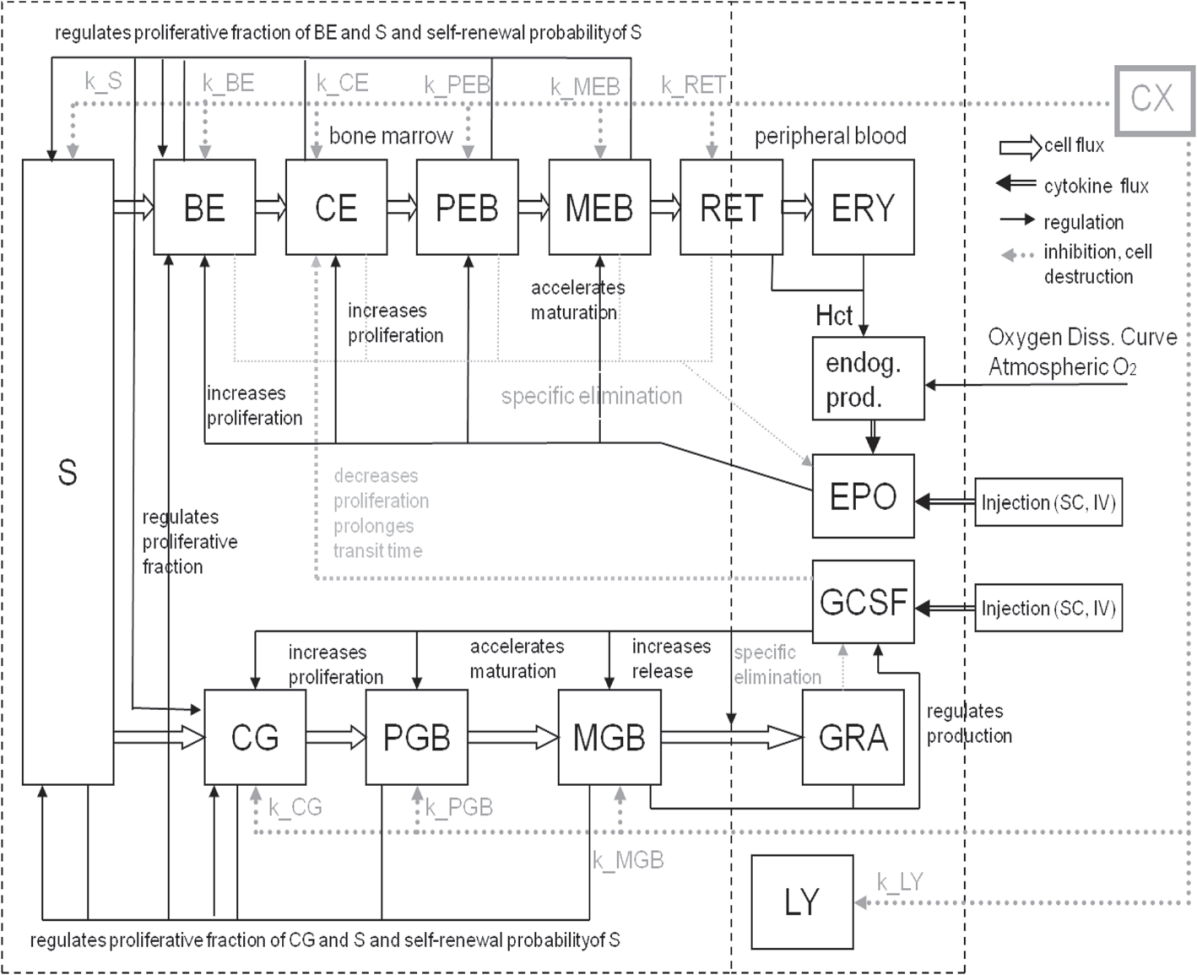
**Structure of the model of erythropoiesis and granulopoiesis under chemotherapy, G-CSF and EPO application.** Model compartments are presented in boxes (S = stem cells, BE = burst forming units - erythroid, CE = colony forming units - erythroid, PEB = proliferating erythrocytic blasts, MEB = maturing erythrocytic blasts, RET = reticulocytes, ERY = erythrocytes, EPO = erythropoietin, CG = granulopoietic progenitor cells (colony forming units of granulocytes and macrophages), PGB = proliferating granulopoietic precursor cells (myeloblasts, promyelocytes, myelocytes), MGB = maturing granulopoietic precursor cells (metamyelocytes, banded and segmented granulocytes)), GRA = granulocytes, LY = lymphocytes, CX = chemotherapy. Several regulatory feedback loops are displayed. The most important two are mediated by EPO and by G-CSF which are produced endogenously and could also be applied externally. Chemotherapy is modelled by a transient depletion of cells.

We aim at constructing a hybrid model of human erythropoiesis and granulopoiesis under chemotherapy, G-CSF and EPO applications by combining an ordinary differential equations model of erythropoiesis [10] and granulopoiesis [3] established recently. The single lineage models have a similar structure: Both consist of a number of concatenated cell compartments representing different proliferating and maturing cell stages in bone marrow and circulation (see Figure 1). Dynamics of cell compartements are described by balance equations of the general form:

(1) $$\begin{array}{l} \frac{d}{\text{dt}}C=A^{\text{in}}\cdot C^{\text{in}}-\frac{C}{T} \end{array}$$

where *A*^in^ is the amplification of the influx derived from the overall amplification *A* (see Additional file 1 section A.1 for details). *T* is the transition time, *C*^in^ is the efflux rate from the preceding cell compartment, and *C* the content of the cell compartment (see [3,4,10-14]).

The quantities *A* and *T* are usually regulated by growth factors, namely EPO for the erythropoiesis model and G-CSF for the granulopoiesis model. Concentrations of these growth-factors as well as external applications are also explicitely modelled. The quantities *A* and *T* are regulated between a minimum and a maximum according to the following sigmoidal functions (*Y*∈{*A*,*T*} in the following)

(2) $$\begin{array}{l} Y=Z_{Y}\left(C_{\text{Cyto}}(t);Y^{\text{min}},Y^{\text{nor}},Y^{\text{max}},b_{Y}\right) \end{array}$$

(3) $$Z_{Y}\left(C_{\text{Cyto}}\right)=\left\{\begin{array}{c} Y^{\text{max}}-(Y^{\text{max}}-Y^{\text{min}})e^{-\ln\left(\frac{Y^{\text{max}}-Y^{\text{min}}}{Y^{\text{max}}-Y^{\text{nor}}}\right){\left(C_{\text{Cyto}}\right)}^{b_{Y}}}\\ \text{for}\;Y^{\text{min}}<Y^{\text{nor}}<Y^{\text{max}}\;\text{or}\;Y^{\text{max}}<Y^{\text{nor}}<Y^{\text{min}}\\ Y^{\text{nor}}\;\text{for}\;Y^{\text{min}}<Y^{\text{nor}}<Y^{\text{max}}, \end{array}\right)$$

where *Y*^nor^ is the corresponding steady state value, $C_{\text{Cyto}}\in \left\{C_{\text{EPO}}^{\text{int}},C_{\text{GCSF}}^{\text{rel}}\right\}$ is the concentration of either EPO or G-CSF and *b*_*Y*_ is the sensitivity of *Y* under stimulation. *Y*^min^ and *Y*^max^ are minimum and maximum values of *Y* respectively. The function 3 is called *regulatory function* in the following (see also [12], p. 69).

Amplification and maturation time may depend on the concentration of the growth factors G-CSF and EPO, denoted as $C_{\text{GCSF}}^{\text{rel}}(t)$ for the G-CSF concentration in the central compartment, or $C_{\text{EPO}}^{\text{int}}(t)$ for the EPO internalised by red blood cells respectively. Details of growth factor mediated regulations as well as assumptions regarding pharmacokinetics and -dynamics of growth-factors are explained in sections ‘Erythropoiesis model’ and ‘Granulopoiesis model’.

Both single lineage models contain similar but not identical models of stem cell dynamics which have a different structure than Equation 1. Since the stem cell compartment is crucial for combining the models, we explain it in more detail in section ‘Stem cell compartment S’. Finally, a model of chemotherapy effects was introduced to the lineage models which is explained in section ‘Chemotherapy model’. A complete set of all model equations can be found in the Additional file 1.

### Erythropoiesis model

The cell kinetic model of erythropoiesis is adopted from earlier modelling works of our group with respect to erythropoiesis in mice and humans [10-13,15,16]. This part of the model describes the development of mature erythrocytes from haematopoietic stem cells and its regulation by endogenous EPO. It consists of the cell compartments S (stem cells), BE (burst forming units - erythroid), CE (colony forming units - erythroid), PEB (proliferating erythrocytic blasts), MEB (maturing erythrocytic blasts), RET (reticulocytes), ERY (erythrocytes), and EPO concentrations of different sites (see also Figure 1).

EPO is assumed to increase the proliferation and to shorten the maturation time of red blood cells in the bone marrow [12,14,16]. Endogenous production of EPO (EPO_prod_) [16] is assumed to depend on the oxygen partial pressure in the kidneys and the number of circulating red blood cells [11,13]. A detailed description of the model of EPO applications can be found in [10]. In brief, it consists of a pharmacokinetic model of EPO (adapted from [17]) and a subcutaneous injection model of EPO (adapted from [18]). The PK model consists of three compartments: EPO in central serum $\left(C_{\text{EPO}}^{\text{cent}}\right)$, the peripheral compartment $\left(C_{\text{EPO}}^{\text{peri}}\right)$, and EPO bound to receptors $\left(C_{\text{EPO}}^{\text{rb}}\right)$[17], with first order transitions between the peripheral and the central compartment describing reversible protein binding of EPO. EPO can bind to free EPO receptors forming a drug-receptor complex which dissociates again or EPO is internalised. EPO is removed from circulation by the latter mechanism or via unspecific elimination modelled by a first order loss term. Internalised EPO serves as argument of all regulation functions regarding EPO.

Different pharmaceutical EPO derivatives are modelled: EPO Alfa, EPO Beta, EPO Delta and Darbepoetin Alfa. The first three are recombinant human EPO derivatives of the first generation differing only in their glycosylation patterns. Darbepoetin Alfa is a next generation EPO pharmaceutical with increased half-life in serum.

Different application sites of EPO result in different absorption kinetics [18]. While intravenous injections can simply be modelled by pulse functions, subcutaneous injections are characterised by delayed and incomplete absorption resulting in a reduced bioavailability [19-21]. To account for this fact, we adapted a model of subcutaneous EPO injection developed for sheep [18]. The model contains two absorption routes: directly from the subcutaneous tissue into the bloodstream, or indirectly via the lymphatic system. In both processes, we included time delays by concatenated sub-compartments with first order transitions (see Additional file 1, section A.3 for details). A loss of EPO is assumed at the injection site and in the lymphatic system. The structure of this injection model is described in detail in [10,18]. All equations can be found in the Additional file 1.

Due to differences in the lymphatic flow at different anatomical regions [18,19,22], we split available injection modi into different groups. While the parameters describing subcutaneuos injection can differ between these groups, the PK parameters are the same for endogenous EPO and all EPO derivatives except for Darbepoetin. Available data allowed to specify parameter settings for injections of EPO Alfa into thigh, EPO Alfa into shoulder, EPO Alfa into forearm, EPO Alfa into upper arm or abdomen, EPO Beta into forearm, EPO Beta into arm or abdomen, EPO Beta into thigh, EPO Delta, and finally, Darbepoetin Alfa (see [10]).

A model of chemotherapy action on erythropoiesis is added as explained in section ‘Chemotherapy model’.

### Granulopoiesis model

The cell kinetic model of granulopoiesis also constructed by our group [3,4,12,23] describes the development of mature granulocytes from haematopoietic stem cells and its regulation by G-CSF. It consists of the cell compartments S (stem cells), the granulopoietic progenitors CG (colony forming units of granulocytes and macrophages), the proliferating granulopoietic precursors PGB (myeloblasts, promyelocytes, myelocytes), the maturing granulopoietic precursors MGB (metamyelocytes, banded and segmented granulocytes), the granulocytes GRA and G-CSF concentrations at different sites (see also Figure 1).

We consider both, endogenously produced G-CSF and injections of the derivatives Filgrastim and Pegfilgrastim. Endogenous G-CSF production is regulated by the demand of mature granulocytes. In analogy to former versions of our model [3,4], this is modelled phenomenologically as a function of the content of the final bone marrow compartment and circulating granulocytes.

Pharmacokinetics of endogenous G-CSF and injections are modelled by three compartments: a subcutaneous compartment $C_{\text{G-CSF}}^{\text{sc}}$ in which G-CSF pharmaceuticals are injected, a central compartment $C_{\text{G-CSF}}^{\text{cent}}$ in which the drugs are pharmacologically active and a peripheral compartment $C_{\text{G-CSF}}^{\text{peri}}$ representing reversible protein binding [24]. This model was originally developed for mice and rats and later adopted for the human situation [3,25,26].

Exogenous G-CSF applications are modelled by an injection function analogous to EPO injections. Delayed influx of injected G-CSF from the subcutaneous compartment into the central compartment caused, e.g., by lymphatic absorption [18] is modelled by division of the subcutaneous compartment into two subcompartments (for details regarding modelling of delays see Additional file 1 section A.3). Dose-dependent bioavailability of G-CSF [26] is modelled by a Michaelis-Menten loss term within the first subcompartment of the subcutaneous tissue [3]. Transitions between central and peripheral compartments were modelled by two-way first order kinetics [27].

G-CSF is eliminated from the central compartment via two routes: Unspecific renal elimination of G-CSF is modelled by a first order kinetic [27,28]. Specific degradation mediated by the number of circulating granulocytes is modelled by a Michaelis-Menten kinetic proportional to this number [3,25,29-32].

We use the same pharmacokinetic and -dynamic parameter settings for Filgrastim and endogenous G-CSF because of their high similarity [33-35]. The observed differences between Filgrastim and Pegfilgrastim [4,28,36-40] are modelled using the same model structure but different parameters settings for absorption, distribution, degradation and regulatory mechanisms (regulatory functions). Receptor competitions between Pegfilgrastim and endogenous G-CSF (or Filgrastim) is modelled by adding the regulatory functions of Pegfilgrastim and Filgrastim using a weighting factor.

Pharmacokinetic and -dynamic modelling of G-CSF is described in detail in [3,4]. In our former model, we assumed a delayed effect of G-CSF on bone marrow cells. Since this effect was rather small, we decided to drop it in the combined model in order to reduce complexity and computational burden.

A model of chemotherapy is attached to our granulopoiesis model as explained in section ‘Chemotherapy model’.

### Stem cell compartment S

Cells differentiating into granulopoietic or erythropoietic lineages originate from a common pool of cells called haematopoietic stem cells ([41]). We used the same equations of stem cell dynamics in our models of granulopoiesis and erythropoiesis, but regulations were slightly different. Basic concepts can be traced back to the book of *Loeffler & Wichmann*[12]. Since the stem cell compartment is crucial for combining the models we present its structure in more detail now.

The stem cell compartment has self-renewal capability. To achieve a steady state, on average 50% of the proliferating stem cells remain in this compartment, and 50% differentiate into red or white blood cell lineages. Hence, the compartment equation differs from Equation 1:

(4) $$\begin{array}{ll} \frac{d}{\text{dt}}C_{S}= & \;(2p-1)C_{S}\frac{a_{S}}{\tau_{S}}\; \end{array}$$

(5) $$\begin{array}{ll} C_{S}^{\text{out}}= & \;2(1-p)C_{S}\frac{a_{S}}{\tau_{S}},\; \end{array}$$

where *τ*_S_ is the average duration of a cell cycle, *p* the self-renewal probability, *a*_S_ is the proliferative fraction, *C*_S_ is the size of the stem cell compartment and $C_{S}^{\text{out}}$ its efflux committing to the haematopoietic lineages. According to [12], the self-renewal probability *p* is regulated by the demand of the hematopoietic bone marrow system. In steady state, we assume $p^{\text{nor}}=\frac{1}{2}$. Thus, for the initial conditions (steady state) it holds that

(6) $$C_{S}(0)=C_{S}^{\text{nor}}=1$$

(7) $$C_{S}^{\text{out}}(0)=C_{S}^{\text{out\_nor}}=2\left(1-p^{\text{nor}}\right)C_{S}^{\text{nor}}\frac{a_{S}^{\text{nor}}}{\tau_{S}}.\;$$

Similar to the stem cell model of [12], self-renewal of stem cells *p* is regulated by a competition of the stem cell content $\left(C_{S}^{\text{rel}}(t)\right)$, and the granulopoietic $\left(C_{G}^{\text{rel}}(t)\right)$ or the erythropoietic $\left(C_{E}^{\text{rel}}(t)\right)$ bone marrow cells.

$$\begin{array}{l} p=p\left(C_{S}^{\text{rel}}(t),C_{E}^{\text{rel}}(t),C_{G}^{\text{rel}}(t),p_{\delta },\vartheta_{E},\vartheta_{G},\vartheta_{S}(t)\right), \end{array}$$

The parameters *ϑ*_E_,*ϑ*_G_, and *ϑ*_S_ are hypothetical weighting factors originally defined as the strength of the influence of the bone marrow cells $C_{S}^{\text{rel}}(t)=\frac{C_{S}(t)}{C_{S}^{\text{nor}}}$, $C_{E}^{\text{rel}}(t)=\frac{C_{\text{BE}}(t)+C_{\text{CE}}(t)+C_{\text{PEB}}(t)+C_{\text{MEB}}(t)}{C_{\text{BE}}^{\text{nor}}+C_{\text{CE}}^{\text{nor}}+C_{\text{PEB}}^{\text{nor}}+C_{\text{BE}}^{\text{nor}}}$, and $C_{G}^{\text{rel}}(t)=\frac{C_{\text{CG}}(t)+C_{\text{PGB}}(t)+C_{\text{MGB}}}{C_{\text{CG}}^{\text{nor}}+C_{\text{PGB}}^{\text{nor}}+C_{\text{MGB}}^{\text{nor}}}$.

According to [12], it is assumed that

$$\begin{array}{l} p_{\delta }=p^{\text{nor}}-p^{\text{min}}=p^{\text{max}}-p^{\text{nor}} \end{array}$$

$$\vartheta_{S}(t)=\left\{\begin{array}{ll} \frac{2}{C_{S}^{\text{rel}}{\left(t\right)}^{0.6}} & \;\text{for}\;C_{S}^{\text{rel}}(t)\leq 1\\ 2 & \;\text{for}\;C_{S}^{\text{rel}}(t)>1 \end{array}\right)$$

(8) $$\begin{array}{l} p=p_{\delta }\tanh\left(-\vartheta_{S}(t)\left(C_{S}^{\text{rel}}(t)-1\right)-\vartheta_{E}\left(C_{E}^{\text{rel}}(t)-1\right)-\vartheta_{G}\left(C_{G}^{\text{rel}}(t)-1\right)\right)+0.5, \end{array}$$

where $p^{\text{nor}}=\frac{1}{2}$, *ϑ*_G_=-8, *ϑ*_E_=-2.

In our single lineage models, this regulation was simplified in different ways. Since erythropoiesis was not included in the model of granulopoiesis, only granulopoietic cells $C_{G}^{\text{rel}}$ were assumed to influence the self-renewal probability in S with the factor *ϑ*_G_=-10, i.e. we assumed $C_{E}^{\text{rel}}=C_{G}^{\text{rel}}$ and thus

$$\begin{array}{l} p=p_{\delta }\tanh\;\left(-\vartheta_{S}(t)\left(C_{S}^{\text{rel}}(t)-1\right)-\vartheta_{G}\left(C_{G}^{\text{rel}}(t)-1\right)\right)+0.5. \end{array}$$

In contrast, a constant granulocyte value $C_{G}^{\text{rel}}=1$ was assumed in our erythropoiesis model. The erythropoietic bone marrow cells $C_{E}^{\text{rel}}$ were multiplied by *ϑ*_E_=-2, thus

$$\begin{array}{l} p=p_{\delta }\tanh\left(-\vartheta_{S}(t)\left(C_{S}^{\text{rel}}(t)-1\right)-\vartheta_{E}\left(C_{E}^{\text{rel}}(t)-1\right)\right)+0.5. \end{array}$$

The proliferative fraction *a*_S_ can be interpreted as the percentage of cells which are currently in cell cycle. The proliferative fractions *a*_*X*_ of the compartments S, BE or CG are also regulated by the haematopoietic bone marrow system $C_{S}^{\text{rel}}(t)$ and $C_{G}^{\text{rel}}(t)$ or $C_{E}^{\text{rel}}(t)$:

$$\begin{array}{l} a_{X}=a_{X}\left(C_{S}^{\text{rel}}(t),C_{E}^{\text{rel}}(t),C_{G}^{\text{rel}}(t),a_{X}^{\text{min}},a_{X}^{\text{nor}},a_{X}^{\text{int}},a_{X}^{\text{max}},\omega_{E},\omega_{G},\omega_{S}\right), \end{array}$$

Again, the parameters *ω*_S_,*ω*_G_, and *ω*_E_ are weighting factors. They represent the strengths of the influence of stem cells, erythropoietic and granulopoietic cells on the proliferative fraction of BE, CG and S respectively. With

(9) $$\begin{array}{lll} x & =\omega_{E}\ln C_{E}^{\text{rel}}(t)+\omega_{G}\ln C_{G}^{\text{rel}}(t)\;\\ & \;+\omega_{S}\left\{\begin{array}{cc} \ln C_{S}^{\text{rel}}(t), & \text{for}\;C_{S}^{\text{rel}}\leq 1\\ C_{S}^{\text{rel}}(t)-1, & \text{for}\;C_{S}^{\text{rel}}>1 \end{array}\right)\; & \;\\ y & =-\frac{1}{2\ln2}\left(\ln\left(\frac{a_{X}^{\text{int}}-a_{X}^{\text{max}}}{a_{X}^{\text{min}}-a_{X}^{\text{int}}}\right)-\ln\left(\frac{a_{X}^{\text{nor}}-a_{X}^{\text{max}}}{a_{X}^{\text{min}}-a_{X}^{\text{nor}}}\right)\right)x\; & \;\\ & \;+\frac{1}{2}\ln\left(\frac{a_{X}^{\text{nor}}-a_{X}^{\text{max}}}{a_{X}^{\text{min}}-a_{X}^{\text{nor}}}\right),\; & \; \end{array}$$

the proliferative fraction is given by

(10) $$\begin{array}{ll} a_{X}= & \left\{\begin{array}{cc} \frac{a_{X}^{\text{max}}e^{-y}+a_{X}^{\text{min}}e^{y}}{e^{-y}+e^{y}}\; & \text{for}\;a_{X}^{\text{min}}<a_{X}^{\text{nor}}<a_{X}^{\text{int}}<a_{X}^{\text{max}}\\ a_{X}^{\text{nor}}\; & \text{for}\;a_{X}^{\text{min}}=a_{X}^{\text{nor}}=a_{X}^{\text{int}}=a_{X}^{\text{max}} \end{array}\right).\; \end{array}$$

It is a monotone function ranging between $a_{X}^{\text{min}}$ and $a_{X}^{\text{max}}$. Low cell numbers in the bone marrow compartments cause a higher demand of proliferating cells, and therefore, a larger proliferative fraction *a*_*X*_. The value of *y* defines the actual point on the regulatory curve. The variable *x* is a measure of the total bone marrow content. It is calculated as a weighted sum of the logarithms of the relative counts of stem cells, erythropoietic cells and granulopoietic cells. If any cell counts tend to zero, *x* tends to minus infinity, and with it, *a* becomes maximal. Parameter values *a*^int^ corresponds to *x*=- ln2 and *a*^nor^ corresponds to *x*=0 (see Figure S2 in the Additional file 1).

In analogy to the regulation of the self-renewal probability, a factor *ω*_G_=0.4 is assumed for the influence of the granulopoietic bone marrow compartments $C_{G}^{\text{rel}}$ in the granulopoiesis model, thus

$$\begin{array}{l} x=\omega_{G}\ln C_{G}^{\text{rel}}(t)+\omega_{S}\left\{\begin{array}{cc} \ln C_{S}^{\text{rel}}(t),\; & \text{for}\;C_{S}^{\text{rel}}\leq 1\\ C_{S}^{\text{rel}}(t)-1,\; & \text{for}\;C_{S}^{\text{rel}}>1 \end{array},\;\right\}. \end{array}$$

In contrast, in the erythropoiesis model we assumed a constant granulocyte value $C_{G}^{\text{rel}}=1$ and the logarithmized erythropoietic bone marrow cells $C_{E}^{\text{rel}}$ are multiplied by *ω*_E_=0.3, i.e.

$$\begin{array}{l} x=\omega_{E}\ln C_{E}^{\text{rel}}(t)+\omega_{S}\left\{\begin{array}{cc} \ln C_{S}^{\text{rel}}(t),\; & \text{for}\;C_{S}^{\text{rel}}\leq 1\\ C_{S}^{\text{rel}}(t)-1,\; & \text{for}\;C_{S}^{\text{rel}}>1 \end{array},\;\right\}. \end{array}$$

### Chemotherapy model

We used the same model of chemotherapy in both the erythropoiesis and the granulopoiesis model. It is based on the following assumptions which are extensively discussed in [2-4]: Chemotherapy results in a delayed, reversible and transient depletion of the compartments S, CG, PGB, MGB, BE, CE, PEB, MEB, and RET, where the toxic effect is specific for the cell stages, for different drugs or drug combinations, and for different doses of the same drug. This is quantified by corresponding sets of toxicity parameters (see tables A.8 and A.9 in the Additional file 1). A higher toxicity in the first chemotherapy cycle is modelled by a factor $f_{\text{fc}}^{\text{drug}}\geq 1$. It is assumed that different cytotoxic drugs damage independently of each other. This allows us to add toxicity functions of chemotherapeutic drugs applied in combinations.

Infusion of chemotherapeutic drugs is again modelled using pulse functions. The effect of chemotherapy is introduced to the balance equations of the bone marrow cell compartments by a first-order loss term *Ψ*_*X*_. Hence, the modified schematic compartment Equation 1 has the form

(11) $$\begin{array}{l} \frac{d}{\text{dt}}C_{X}=A^{\text{in}}\cdot C_{X}^{\text{in}}-\frac{C_{X}}{T}-\Psi_{X}\cdot C_{X} \end{array}$$

for *X*∈{CG, PGB, MGB, BE, CE, PEB, MEB, RET}. *Ψ*_*X*_ depends on the kind, dose and timing schedule of chemotherapy as well as on the affected bone marrow comparted *X* (see Additional file 1 for further details). Analogously, the modified stem cell Equation 4 has the form

(12) $$\begin{array}{ll} \frac{d}{\text{dt}}C_{S}= & \;(2p-1)C_{S}\frac{a_{S}}{\tau_{S}}-\Psi_{S}\cdot C_{S}.\; \end{array}$$

In clinical practice often only leukocytes, i.e. the sum of unspecific (granulocytes) and specific (lymphocytes) immune cells, are available. To avoid additional modelling of lymphopoiesis, the reduced lymphocyte count under chemotherapy is described by an exponential function of the form (see [3]):

$$C_{\text{WBC}}\left(t\right)\approx c_{\text{LY}}\exp\left(-\Psi_{\text{LY}}\left(t\right)\right)+c_{\text{GRA}}\frac{C_{\text{GRA}}\left(t\right)}{C_{\text{GRA}}^{\text{nor}}},$$

where *c*_LY_=3000 cells per *μl* and *c*_GRA_=4000 cells per *μl* are the steady state concentrations of lymphocytes and granulocytes respectively. *Ψ*_LY_ is the toxicity function for lymphocytes which is analogously defined as the toxicity functions of bone marrow cell stages.

Age is a major risk factor of haematotoxicity under chemotherapy [1]. In our former granulopoiesis model [4], we assumed different toxicity parameters for the age groups ≥60 years and <60 years. This assumption will be carried over to our hybrid model.

### Combination of granulopoiesis and erythropoiesis models

It is our major intention to keep the single lineage models as unchanged as possible. But a few assumptions are necessary to combine them. These assumptions refer to the regulation of stem cells, the interaction of granulopoiesis and erythropoiesis, and common chemotherapy effects.In the following, we describe the assumptions and necessary modifications of the single lineage models in detail. A schematic representation comprising all parts of the model is shown in Figure 1.

#### Common stem cell compartment

In the single lineage models, the concurrent influence of erythropoietic and myeloid cells on the regulation of the stem cell compartment, i.e. the regulation of self-renewal probability and proliferative fraction, could not be considered. This resulted in a number of simplifications explained in section ‘Stem cell compartment S’. When combining the single lineage models, these simplifications are no longer required. Hence, we rely on the extended regulatory functions regarding proliferative fraction and self-renewal (Equations 8, 9 and 10). The same regulatory principles were used for the proliferative fractions of the compartments BE and CG, i.e. these quantities now also depend on both, relative stem cell count ($C_{S}^{\text{rel}}$), relative size of bone marrow erythropoiesis ($C_{E}^{\text{rel}}$) and granulopoiesis ($C_{G}^{\text{rel}}$).

Furthermore, we implemented a splitting of the output of the stem cell compartment which feeds both lineages. We assume that 15% of the released cells ($C_{S}^{\text{out}}$, see Equation 7), differentiate into red blood cells (*α*_E_=0.15), and 80% into the white blood cell line (*α*_G_=0.8, see also [12], pp. 61, 62). However, these parameters are dummies having no impact on the behaviour of the model in its current form since only relative changes of compartment sizes are considered. They will become relevant later when implementing a model of lineage commitment [42]. Hence, the influx into the subsequent erythropoietic and granulopoietic progenitor compartments reads as follows:

$$\begin{array}{lll} C_{\text{BE}}^{\text{in}}= & \;0.15C_{S}^{\text{out}}\; & \;\\ C_{\text{CG}}^{\text{in}}= & \;0.8C_{S}^{\text{out}}\; & \; \end{array}$$

Note that 5% of stem cells are assumed to commit to thrombopoietic lineage.

#### Compartment CE and G-CSF effect on erythropoiesis

**Figure 2:**
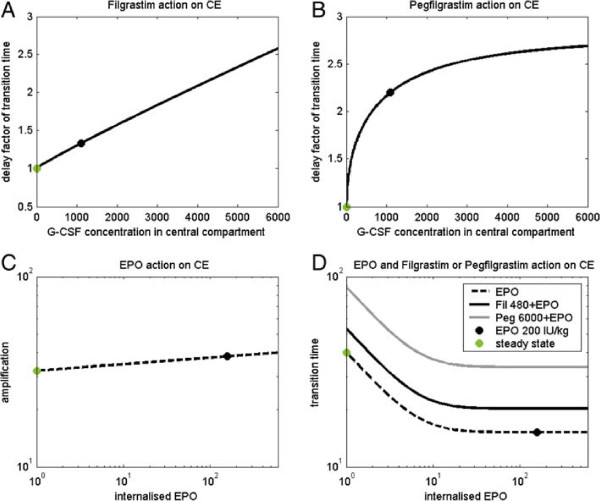
**Regulatory functions in compartment CE.** Regulatory functions describing the effect of Filgrastim, Pegfilgrastim, EPO and combinations of it on amplification rate and transition time in the compartment CE. While amplification is assumed to be unaffected by G-CSF, the transition time is delayed under G-CSF stimulation. **A**: Delay factor of transition time - dependence on endogenous G-CSF/Filgrastim concentration. We mark the values achieved at steady-state (green) and at maximum after a single s.c. application of 480 *μg* (black). **B**: Delay factor of transition time - dependence on Pegfilgrastim concentration. Values achieved at maximum after a single s.c. application of 6000 *μg* (black) and steady state (green) are marked. **C**: Amplification in CE - dependence on internalised EPO. No G-CSF effects are assumed here. **D**: Transition times in CE - dependence on internalised EPO and G-CSF concentration. We present the raw regulatory functions without considering G-CSF effects (dotted) and maximal deviations after either Filgrastim application of 480 *μg* (solid) or Pegfilgrastim application of 6000 *μg* (grey). Note that the regulatory functions presented in A and B are superimposed (Equation 16) under Pegfilgrastim or combined Pegfilgrastim/Filgrastim applications.

In our single lineage model of erythropoiesis, the amplification and transition time in compartment CE is only regulated by the growth factor EPO. Now we additionally assume an inhibiting influence of endogenous G-CSF, Filgrastim and Pegfilgrastim on this compartment. Evidence for interactions of Erythropoietin and G-CSF and mutual influence of the different lineages is described in [7,8,43].This interaction is modelled by additional regulatory functions of G-CSF multiplied to those of amplification and transition time of the compartment CE (see Equation 3 and Figure 2). More precisely, we have

(13) $$A_{\text{CE}}=Z_{A_{\text{CE}}}\left(C_{\text{EPO}}^{\text{int}}\right)\cdot F_{\text{A\_GCSF}}(t)$$

(14) $$T_{\text{CE}}=Z_{T_{\text{CE}}}\left(C_{\text{EPO}}^{\text{int}}\right)\cdot F_{\text{T\_GCSF}}(t).$$

with

(15) $$F_{\text{A\_GCSF}}(t)=w_{P}(t)\cdot Z_{\text{A\_Peg}}(t)+(1-w_{P}(t))\cdot Z_{\text{A\_Fil}}(t)$$

(16) $$F_{\text{T\_GCSF}}(t)=w_{P}(t)\cdot Z_{\text{T\_Peg}}(t)+(1-w_{P}(t))\cdot Z_{\text{T\_Fil}}(t)$$

where $Z_{A_{\text{CE}}}$, $Z_{T_{\text{CE}}}$ are the regulatory functions of amplification and transition time in compartment *CE*, $Z_{\text{T\_Fil}}(t)$, $Z_{\text{A\_Fil}}(t)$ and $Z_{\text{A\_Peg}}(t)$, $Z_{\text{T\_Peg}}(t)$, are regulatory functions of endogenous G-CSF respectively of Filgrastim and Pegfilgrastim, 0≤*w*_*P*_(*t*)≤1 is the weighting factor to model the superimposing effect of concurrent endogenous G-CSF / Filgrastim and Pegfilgrastim action (see [3]), and $C_{\text{EPO}}^{\text{int}}$ is the internalised EPO (see [10]). The fitting process performed later resulted in a rather small influence of Filgrastim and Pegfilgrastim on the amplification in CE. Therefore, we decided to set $F_{\text{A\_GCSF}}(t)=1$. A complete set of parameters regarding this regulation is listed in Table 1.

**Table 1:** Parameters for modelling G-CSF effects on compartment CE

**Parameter**	**Meaning**	**Filgrastim**		**Pegfilgrastim**	
$T_{{\text{CE}}_{F}}^{\text{min}},T_{{\text{CE}}_{P}}^{\text{min}}$	Factor of transition time under minimal stimulation	0.9995	Fitted	1	Set
$T_{{\text{CE}}_{F}}^{\text{nor}},T_{{\text{CE}}_{P}}^{\text{nor}}$	Factor of transition time under normal stimulation	1	Set	1.037	Fitted
$T_{{\text{CE}}_{F}}^{\text{max}},T_{{\text{CE}}_{P}}^{\text{max}}$	Factor of transition time under maximal stimulation	98.37	Fitted	2.787	Fitted
$T_{{\text{CE}}_{F}}^{b},T_{{\text{CE}}_{P}}^{b}$	Sensitivity of factor of transition time	0.930	Fitted	0.5660	Fitted

#### Common chemotherapy model

The same chemotherapy model was used in our single lineage models of granulopoiesis and erythropoiesis. However, combination of the models required some adjustments of toxicity parameters due to the interactions of the lineages not considered in the previous models.

### Numerical methods for simulation

Simulations were performed with MATLAB 7.5.0.342 (R2007b) using the SIMULINK toolbox (The MathWorks Inc., Natick, MA, USA). Numerical solutions of the equation system are obtained using the variable step solver from Adams and Bashford (ode113, SIMULINK toolbox).

### Data sets

We combined the data sets used to establish the single lineage models of erythropoiesis and granulopoiesis. These data sets comprise time courses of absolute neutrophil counts (ANC), white blood cell counts (WBC), hemoglobin (HB), hematocrit (HCT), serum concentration of EPO and G-CSF, red blood cell counts (RBC), reticulocyte counts (RET), or percentages of reticulocytes after EPO or G-CSF application in healthy volunteers and time series data of hemoglobin, ANC or WBC in patients treated with chemotherapy with or without G-CSF or EPO support. Data sets without access to raw data were taken from literature. The automated tool “ycasd” [44] was used to extract the data as precisely as possible. However, in literature often only data of one blood cell lineage per scenario were available.

We also have access to raw patients data of the German High Grade Non-Hodgkin’s-Lymphoma Study Group [45-48] and the German Hodgkin’s Lymphoma Study Group [49,50], and the German Breast Group [51]. These data comprise both, haemoglobin and leukocyte data for 11 different chemotherapeutic drug combinations. Considering different growth factor schedules, we analysed raw data of 15 different therapy scenarios. Patients HB data were censored after receiving erythrocyte concentrates. For some therapy schedules we split the data taking into account individual risk groups (young vs. elderly patients). Combined with the literature data, we modelled 15 different chemotherapeutic drugs or drug combinations. Taking into account differences in growth factor schedules results in a total of 33 different therapy schemes. An overview of all data sets used for modelling is presented in table A.10 in the Additional file 1.

Scenarios were either used to determine unknown parameters of the model or to validate model predictions.

### Estimation of parameters

It is our goal to keep the parameters of our single lineage models constant as far as possible to reduce the problem of over-fitting, but a few adaptations were necessary due to the changes required for model combination (see section ‘Combination of granulopoiesis and erythropoiesis models’). While the parameters of the cell kinetic models of granulopoiesis and erythropoiesis and the pharmacokinetics of EPO and G-CSF described in [3,4] and [10] were kept constant, we modified chemotherapy parameters and those involved in regulating amplification and transition time in the modified compartment CE (see tables of chemotherapy parameters in the Additional file 1 and Table 1).

To estimate toxicity parameters, data of at least one scenario are required in which the corresponding drug or drug combination was used. Hence, chemotherapy parameters were established using the EC-T and E-T-C scenarios with G-CSF and with or without EPO [51], CHOP 21 and CHOEP 21 for young and elderly patients [45,46], the BEACOPP 21 and BEACOPP 21 escalated data [49,50], the high CHOEP 21 data [47], the literature data with ESHAP and 12 × 5 *μ*g/kg Filgrastim or 100 *μ*g/kg Pegfilgrastim on day 6 [52] and the literature data with Platinum and Etoposide with and without Darbepoetin [53]. Toxicity parameters for the literature scenarios without HB data (Doxorubicin-Docetaxel [54-57] or Carboplatin-Paclitaxel [58]) were taken from the granulopoiesis model. Due to lack of data, toxicity parameters for erythropoiesis could not be established for these scenarios.

Parameters regarding Filgrastim effects on the compartment CE were determinated using the HB- and WBC-data of the NHL-B trial (CHOP 14, elderly patients, Filgrastim 480 *μ*g day 4–13), the data of E-T-C with EPO and G-CSF on day 3–10, and the data of the “Ricover” trial (elderly patients, CHOP 14, G-CSF Filgrastim 480 *μ*g day 6–12), since these scenarios have simple chemotherapy regimens but different G-CSF schedules informative for our purposes. Parameters regarding Pegfilgrastim effects on the compartment CE were determinated on the basis of the data of CHOP 14 with Pegfilgrastim on day 2 and day 4 [5].

For parameter fitting, (1+3)-evolutionary-strategies with self-adapting mutation step size [59,60] were used. This refers to a non-deterministic optimisation algorithm with one possibly immortal parent and three children per generation. We applied the fitness function described in [3,10] based on the area between simulation and data curve:

(17) $$\int_{t_{0}}^{t_{1}}\left|\log(f_{\text{model}}(t,\text{k}))-\log(f_{\text{data}}(t))\right|\text{dt}\to \underset{\text{k}}{\min}.$$

*f*_model_(*t*,**k**) is the simulation result at time *t* for the parameter set **k**=*k*_1_,…*k*_*n*_. *t*_0_≤*t*≤*t*_1_ describe the range of time points for which data are available, and *f*_data_(*t*) is the interpolated curve of data medians. Since we model relative sizes of cell compartments, model outputs are multiplied by normal values to allow comparisons with clinical data.

The fitness value is defined as the left hand side of Equation 17. It is specific for the clinical intervention (e.g. application of a certain chemotherapy), the outcome measure (e.g. granulocyte counts) and the clinical data set (e.g. median of patient data at certain time points) considered. Fitness values of different outcomes of the same clinical intervention are added to calculate an overall fitness value of the scenario. For simultaneous fitting of more than one scenario, we add corresponding fitness values.

We performed a sensitivity analysis of newly introduced cell kinetic parameters of our model by changing their values by 2.5% and calculating corresponding deteriorations of the fitness function. It revealed that minimum and normal values of the G-CSF regulations of compartment CE (see Table 1) have considerably higher precision than corresponding estimates of maximum values and b-parameters (details see Additional file 1: Figure S4).

### Validation of model

Most of the scenarios for which we have data were not used for parameter fitting but for validation purposes. This requires that necessary parameters for the simulation of a particular scenario were either determined by previous fitting steps or can be taken from the single lineage models.

Throughout, scenarios with growth-factor administration in healthy volunteers were used to validate our hybrid model (data sets 1–46, 61–84 in table A.10 of the Additional file 1), since the majority of required parameters were determined in the framework of the single lineage models.

The situation for scenarios including chemotherapy is more complicated: At least one scenario must be fitted to determine the toxicity parameters of an unknown drug combination, but often, toxicity parameters determined in the single lineage models performed also fine for the hybrid model. This applied for the scenarios 85–96 of table A.10 comprising literature data of Carboplatin and Paclitaxel or Doxorubicin and Docetaxel with injections of Filgrastim or Pegfilgrastim or CHOP-like therapies (therapies which are similar to the CHOP combination therapy consisting of the drugs Cyclophosphamide, Doxorubicin, Vincristin and Prednisone). Additionally, the data of high CHOEP 14 (data set 57), CHOEP 14 and CHOP 14 (data sets 53 and 55, subgroup of young patients) were also not included into the fitting procedure but used for validation. For the majority of chemotherapy scenarios, only WBC or ANC data are available. Hence, validation of the granulopoiesis sub-model relies on a considerably larger data base.

## Results

### Model behaviour

**Figure 3:**
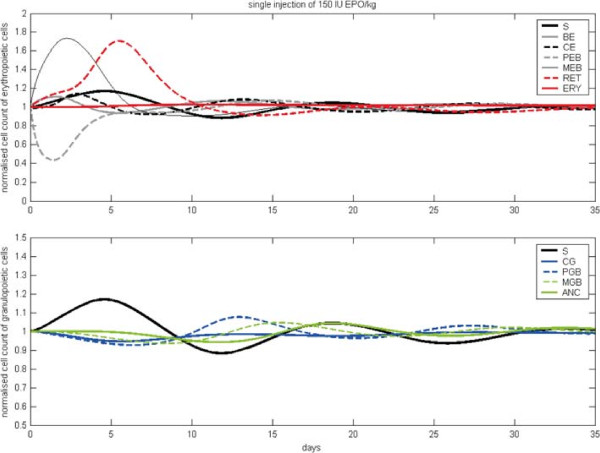
**Model behaviour after single injection of EPO.** We present cell counts normalised to steady state values after i.v. injection of 150 IU/kg EPO Alfa. After damped oscillations of compartment sizes the system converges to equilibrium state.

**Figure 4:**
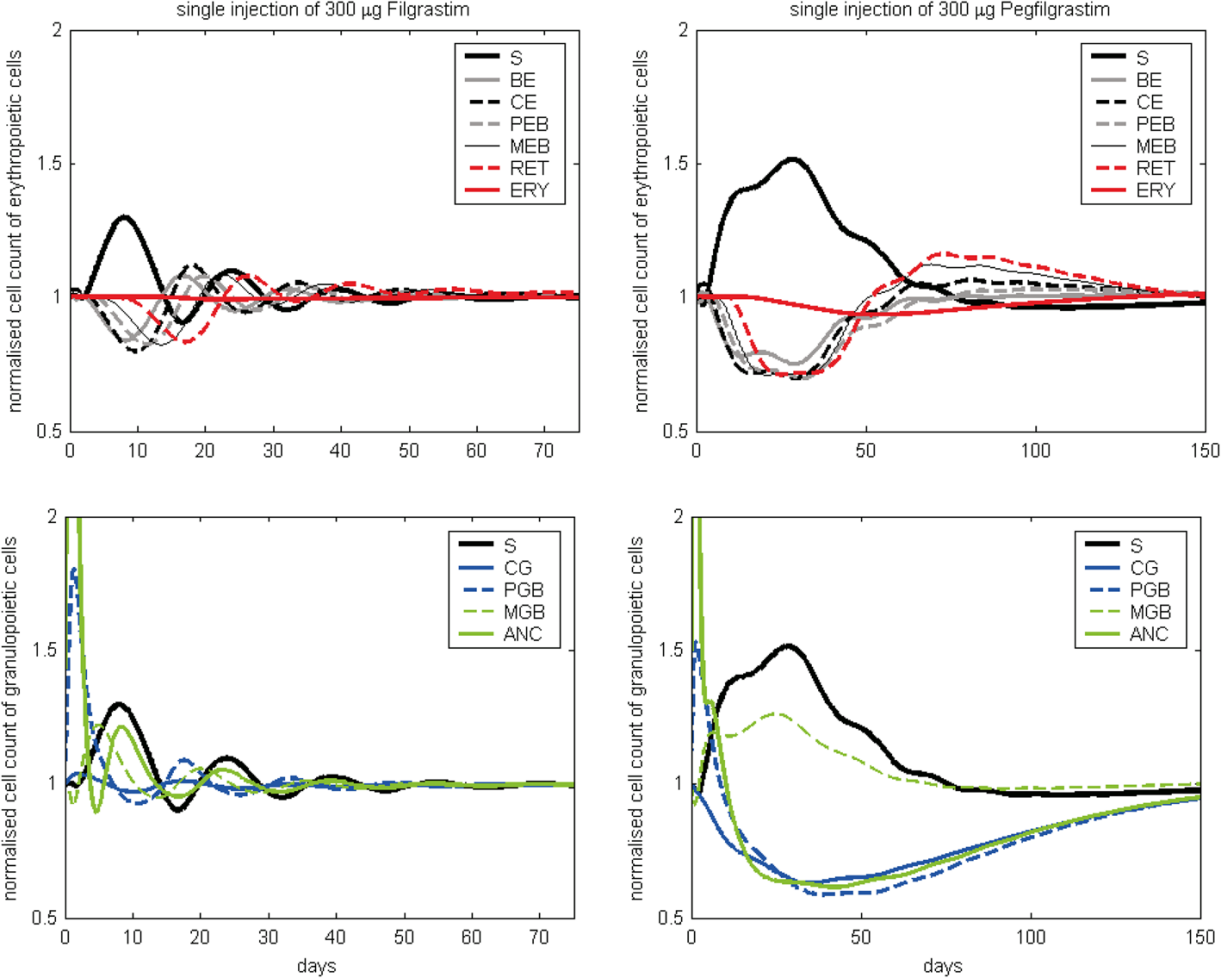
**Model behaviour after single injection of G-CSF.** Model behaviour after perturbation with respect to steady state values. We present cell counts normalised to steady state values after a single s.c. injection of 300 *μg* Filgrastim (left) or 300 *μg* Pegfilgrastim (right). A single Filgrastim injection results in damped oscillations of compartment sizes, which was not observed for Pegfilgrastim.

**Figure 5:**
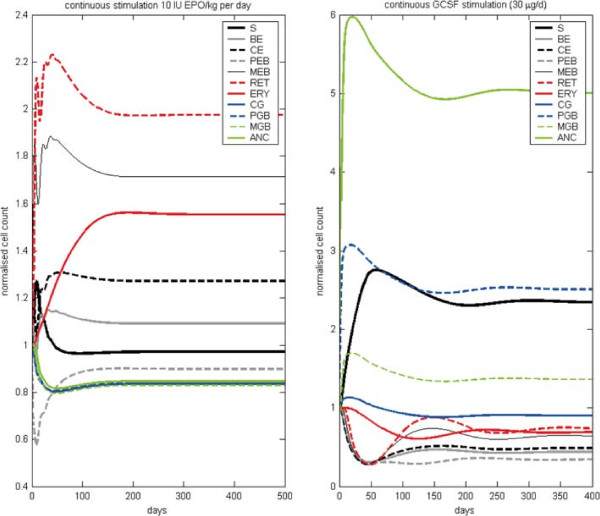
**Model behaviour during continuous stimulation with EPO or G-CSF.** Left: EPO, right: Filgrastim. New steady states are reached after a certain time.

After calibration, we study the qualitative behaviour of the model and compare it later with the results of the single lineage models. In our hybrid model, we have additional feedbacks due to the G-CSF effect on CE cells, the indirect effect of erythropoietic bone marrow cells on granulopoiesis and vice versa via regulation of the proliferative fractions of S, CE, BE and CG as well as the regulation of stem cell self-renewal probability. Feedbacks are negative throughout resulting in more or less damped oscillations of compartment sizes after disturbance of the system. This behaviour is robust against changes of model parameters except for those involved in stem cell feedback: If proliferative fraction and stem-cell self-renewal are allowed to respond more intensively to changes of bone marrow contents, disturbances of the system result in stable oscillations, a phenomenon which was formerly used to explain cyclic neutropenia in dogs ([61,62], see Figure S3 in the Additional file 1).We study model dynamics after single perturbation and compare them with the results of the single lineage models (see section ‘Comparison of the single lineage models and the hybrid model’). At first, we simulate a single i.v. injection of 150 IU/kg EPO Alfa at t =0 (see Figure 3): The MEB compartment of the hybrid model expands quickly, followed by a peak in reticulocytes. Since initially, amplification in BE and CE is stimulated by EPO, these compartments also show increasing cell counts. The compartment S also increases initially, caused by the up-regulation of the self-renewal probability due to increased bone marrow cell counts. In contrast, the compartment PEB is depleted by the shortened maturation time and thereby increased efflux into the compartment MEB. After a minimal decrease in the number of CG and PGB the granulopoietic cells follow the oscillation in S. Overall, damped oscillations of the compartment sizes can be observed. Next, we simulated a single s.c. injection of Filgrastim at t =0 (see Figure 4 left panels): ANC grows fast caused by the increased release of mature granulocytes from bone marrow to circulation. Thereafter, the GRA compartment is reduced rapidly caused by the fast degradation of Filgrastim and the short half life of granulocytes. Since the proliferation in PGB is increased by G-CSF, this compartment also grows initially. The number of stem cells increases (similarly to EPO application) due to up-regulation of self-renewal. The number of erythropoietic cells is decreased due to the G-CSF effect on CE. Again, all cell counts show damped oscillations, which is typical for short-term disturbances of the system. After Pegfilgrastim administration (see Figure 4 right panels), the cell numbers react similarly, but increments and reductions of cell counts remain for a longer time frame than after Filgrastim application. In contrast to Filgrastim application, no oscillations occur after Pegfilgastim application. The reason for this behaviour is the longer half-life of Pegfilgrastim compared to Filgrastim. Pegfilgrastim applications result in prolonged stimulations of the bone marrow, and with it, an expanded stem cell pool which is only gradually reduced after elimination of Pegfilgrastim.Now we study the effects of continuous stimulation with EPO and G-CSF (see Figure 5). For this purpose, we keep EPO and G-CSF serum concentrations constant at higher values. This is achieved by constant influxes into these compartments. EPO stimulation results in rapidly increasing numbers of MEB and RET. After oscillation, a new steady state of BE, CE, MEB and RET on a higher level compared to the unperturbed steady state is achieved. In contrast, the cell number in PEB reaches a slightly lower equilibrium after an initial strong decline. After an initial peak the stem cells stabilise on a slightly lower level. The numbers of CG, PGB, MGB and ANC decline to a lower steady state.

A continous administration of G-CSF results in an increased equilibrium of PGB, MGB, ANC and S. Compartments CG, BE, CE, PEB, MEB, RET and ERY stabilise on lower levels.

**Figure 6:**
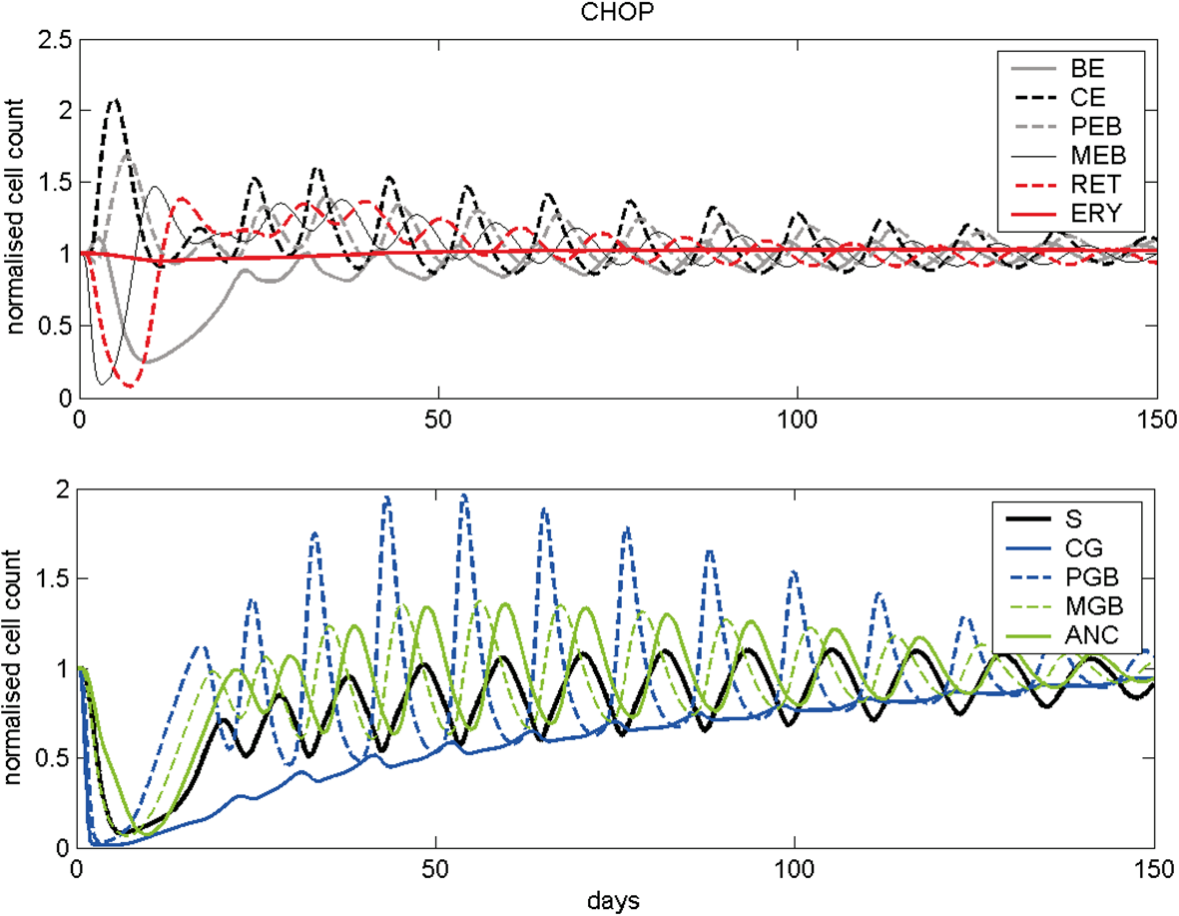
**Model behaviour after a single chemotherapy administration.** We present cell counts normalised to steady state values after a single administration of CHOP chemotherapy. Except for erythrocytes, all lineages show damped oscillations over a longer time period.

Finally, we want to study the system behaviour after a single CHOP chemotherapy administration at *t*=0. Figure 6 illustrates the reaction of the system to this damaging effect. After an initial growth of the numbers of CE and PEB, damped oscillations of both lineages appear over a longer time period. The numbers of BE, MEB and RET show a strong decline after chemotherapy administration. Later, they show damped oscillations as well while they slowly approach their normal values. Erythrocytes are slightly diminished after chemotherapy and later their number grows slowly without oscillations. The compartments S, CG, PGB, MGB and ANC were strongly depleted after chemotherapy application. Later their numbers oscillate and they slowly converge to their normal values.

### Comparison of the single lineage models and the hybrid model

**Figure 7:**
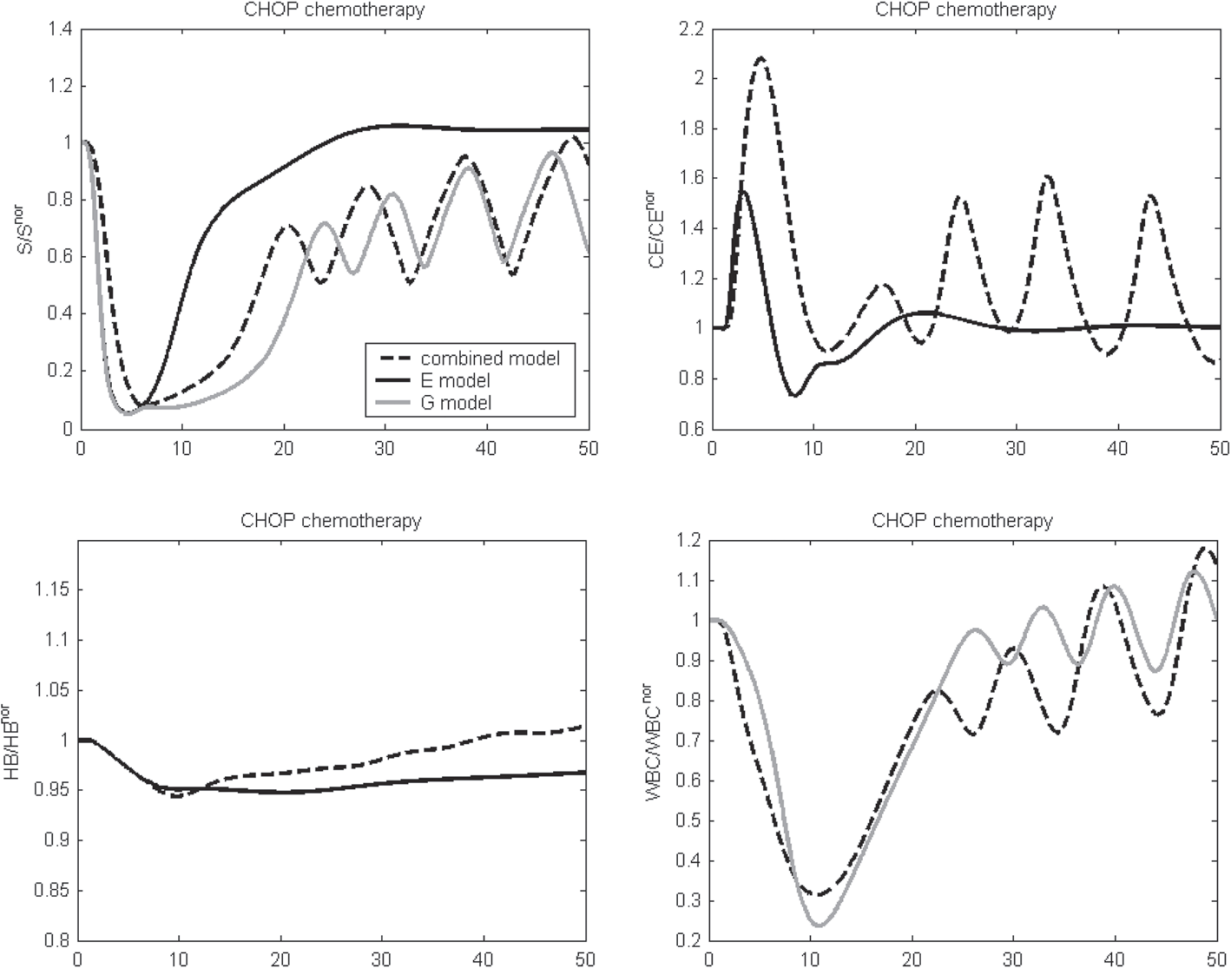
**Comparison of dynamics after CHOP chemotherapy.** Comparison of the behaviour of the combined and the single lineage models after perturbation with CHOP chemotherapy. We present stem cell dynamics, CE, HB and WBC normalised to steady state values.

In this section, we compare simulation results after perturbations of the hybrid model with corresponding results of the single lineage models. As in the previous sections, we simulate single injections of EPO, Filgrastim, Pegfilgrastim and CHOP chemotherapy (see Figure 7) respectively. Newly implemented interactions between erythropoiesis and granulopoiesis in the hybrid model resulted in some differences (for further details, see section “Model comparison” in A.9 of the Additional file 1). Generally, in the hybrid model, stem cell oscillations are less damped than in the single lineage models, especially after chemotherapy (CHOP). Interestingly, despite this observation, the simulation results for HB and WBC are comparable between the hybrid and the single cell lineage models, i.e. the lack of damping in S is compensated at later cell stages. Nevertheless, we predict continued oscillations of mature blood cells after chemotherapy, an issue which could be verified in patients.

### Chemotherapy scenarios

Chemotherapy toxicity parameters were re-estimated by fitting the predictions of the model to our data sets (see tables of chemotherapy parameters in the Additional file 1 and section ‘Estimation of parameters’).

**Figure 8:**
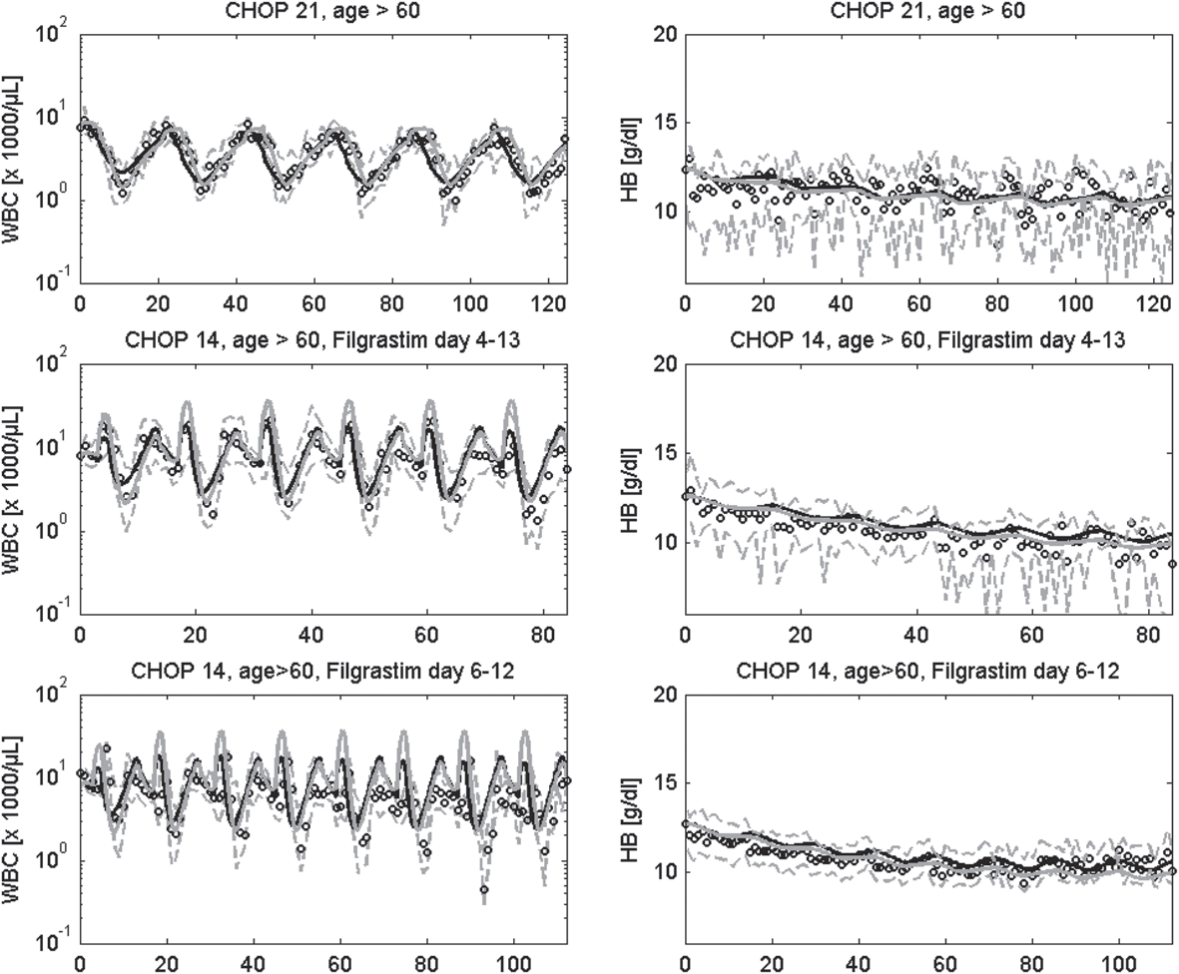
**Model simulations of different CHOP chemotherapies with or without Filgrastim support.** First row: Six cycles of CHOP with cycle duration of 21 days without Filgrastim, second row: Six cycles of CHOP with cycle duration of 14 days with Filgrastim at day 4–13 at each therapy cycle, third row: Eight cycles of CHOP with cycle duration of 14 days with Filgrastim at day 6–12 at each therapy cycle. We present time series of HB and WBC simulated with our combined model (black solid line) and compare it with corresponding simulation results of the single lineage models (grey solid line). We also compare model results with our clinical data (median: circles, first and third quartile: grey dashed line) taken from the trials published in [45,63].

**Figure 9:**
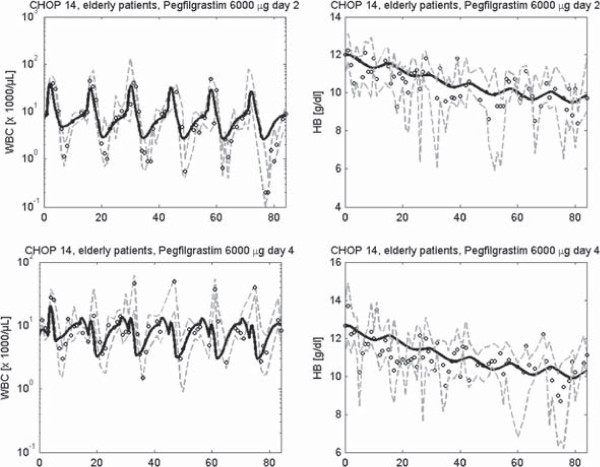
**Model simulations of six cycles of CHOP chemotherapy with cycle duration of 14 days and different Pegfilgrastim support.** We present time series of HB and WBC simulated with our combined model (black solid line). We compare model results with our clinical data (median: circles, first and third quartile: grey dashed line) taken from clinical trials [5].

**Figure 10:**
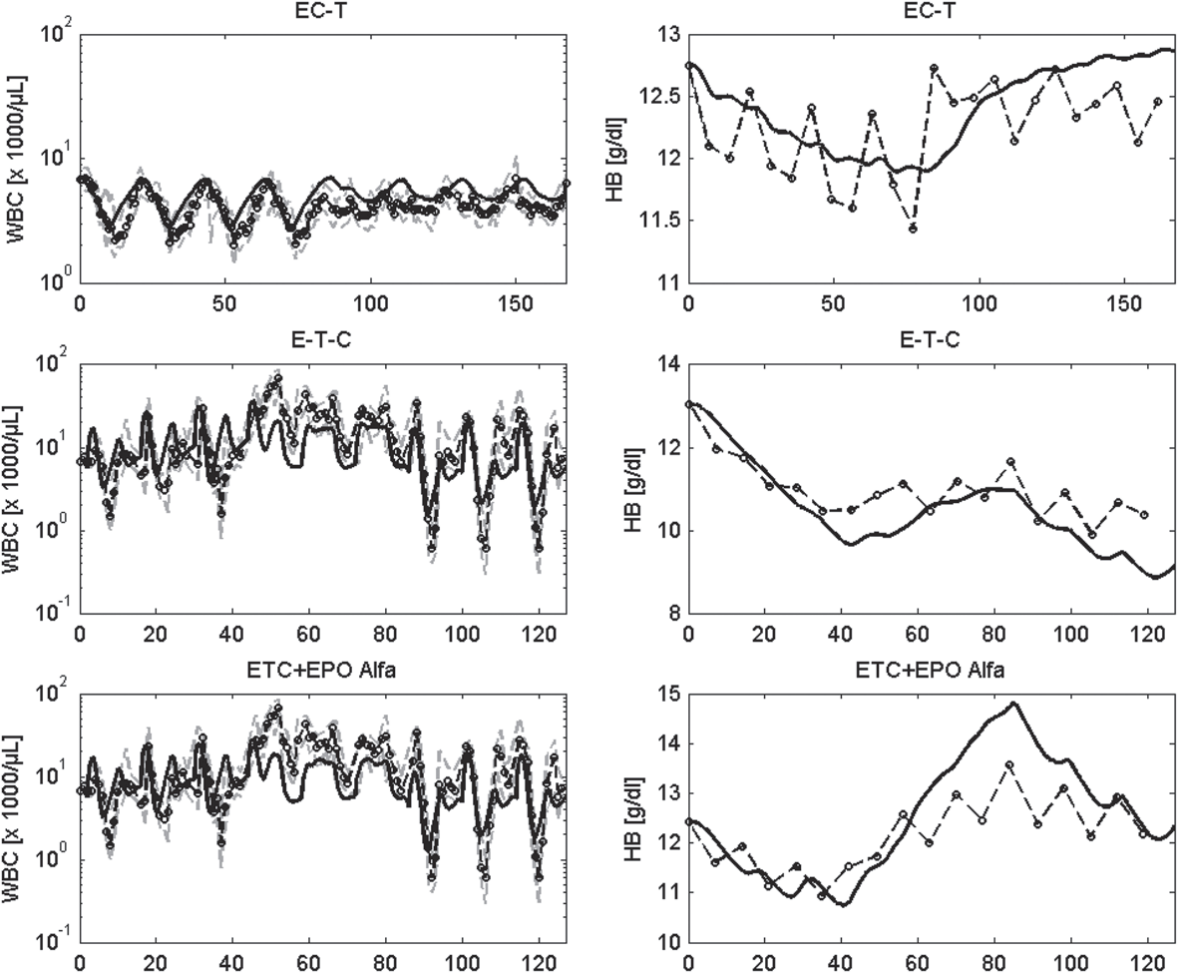
**Hybrid model simulations after chemotherapy, Filgrastim or EPO.** We present results of different breast cancer therapies. First row: four cycles of the drug combination epirubicine + cyclophosphamide followed by four cycles of paclitaxel with cycle duration of 21 days without G-CSF, second row: three single drug cycles of epirubicine, paclitaxel, cyclophosphamide applied consecutively with cycle duration of 14 days with Filgrastim on cycle days 3–10, third row: The same therapy as described in second row but with additional EPO Alfa. We show HB and WBC values of simulation (black line), medians (circle), 25th and 75th percentiles (grey dashed line) of patients data. While raw data are available for the first two scenarios, data of the third scenario are taken from [51].

In Figures 8, 9 and 10 we present a selection of chemotherapy scenarios for which WBC and HB data are available, i.e. which could not completely be described by our former single lineage models. Scenarios comprise CHOP chemotherapy for the treatment of aggressive non-Hodgkin’s lymphoma disease with different dosing and timing schedules of Filgrastim (Figure 8) or Pegfilgrastim (Figure 9) and breast cancer therapies supported by Filgrastim and EPO (Figure 10). Note that these are multi-cycle therapies in which a drug or drug combination is applied multiple times, usually with 14 days or 21 days intervals (see figure legends and table A.10 in the Additional file 1 for details). We obtained a good agreement of model and data for all scenarios. Simulation results of the single cell lineage models and the combined model are comparable throughout. In the Additional file 1 we present the results of further chemotherapy scenarios (Figures S27, S33–S40, compare with table A.10).

### Validation

**Figure 11:**
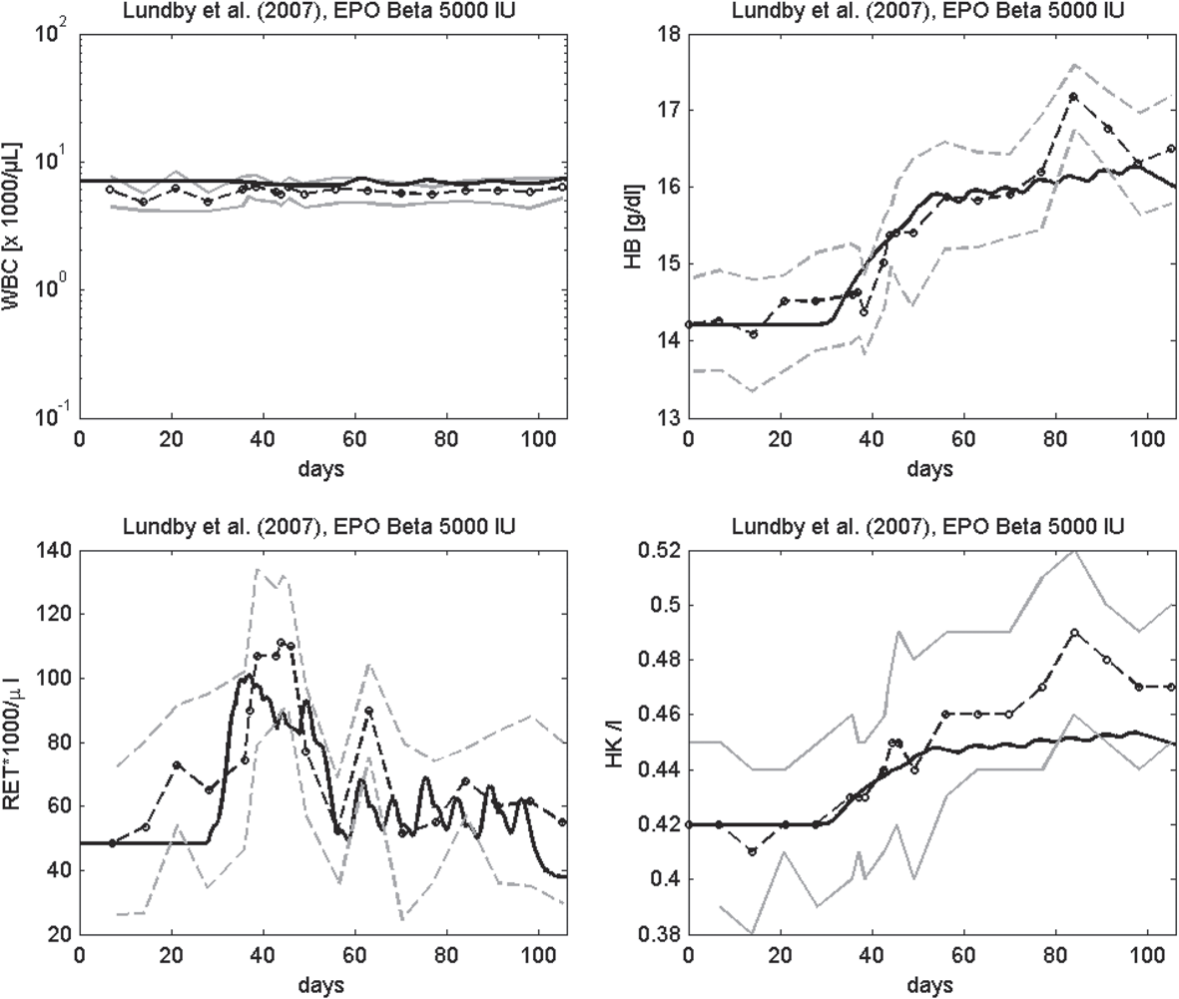
**Example validation scenario.** Data of Lundby et al. [64] after multiple injections of EPO Beta. Time series of WBC, HB, Reticulocytes and HK are presented. Solid black curve represents simulation results. Circles and grey line represent means and standard deviations respectively [64].

Scenarios not included into the fitting procedure are suitable for validation of the hybrid model. For example, in [64] time series data of reticulocytes, WBC, HB and HK after multiple injections of 5000 IU EPO beta into healthy volunteers are presented. This scenario was not included into the fitting procedure of both single lineage models as well as the hybrid model. For the latter one, we observed a good fit of the data of both cell lineages over a longer time period of 15 weeks (see Figure 11).

Results of the other validation scenarios can be found in the Additional file 1. We observed a reasonable fit for all of these scenarios demonstrating the validity of our model.

## Model predictions

**Figure 12:**
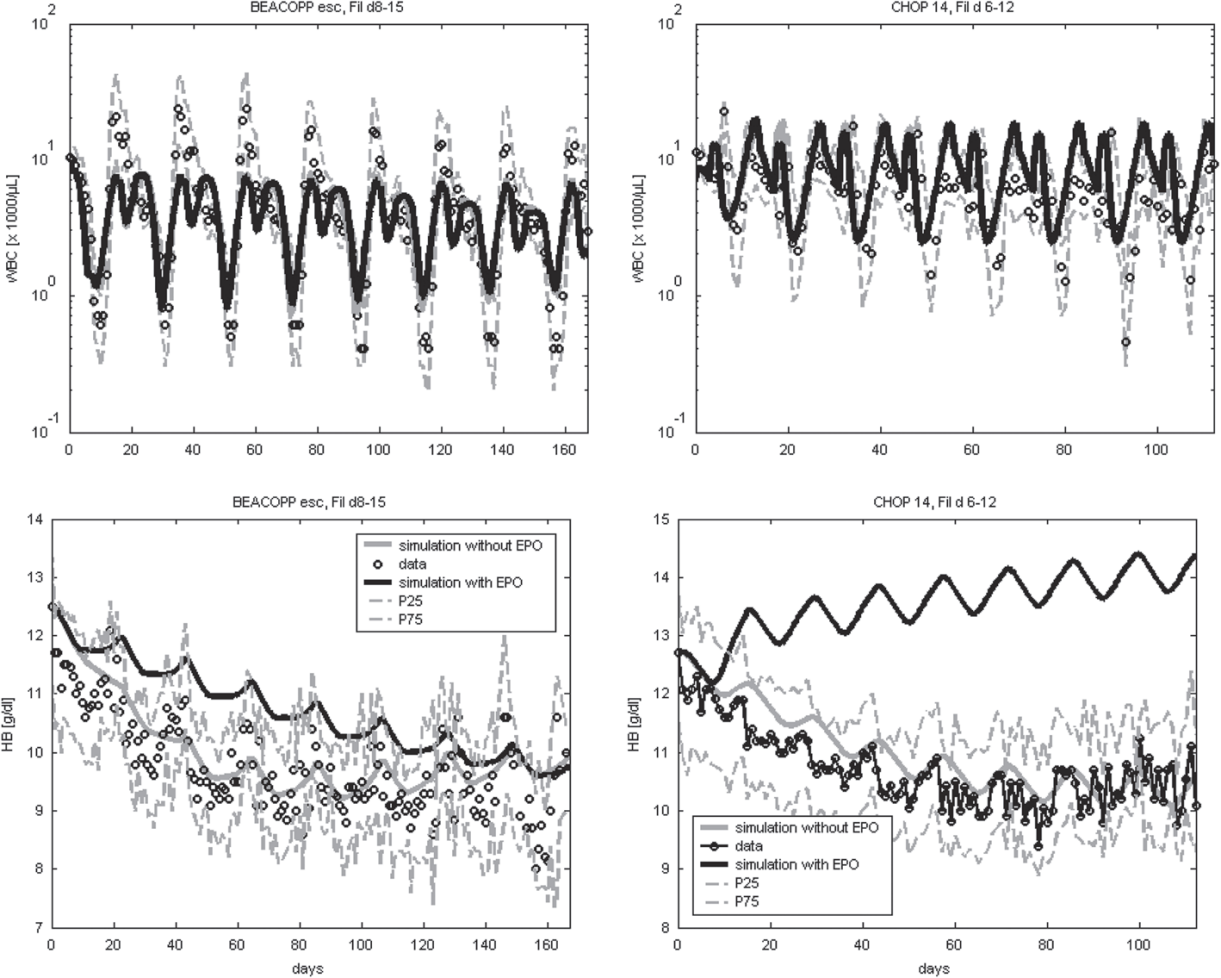
**Prediction scenarios.** We present predictions regarding BEACOPP-escalated and CHOP 14 (elderly) supported by Filgrastim and weekly Darbepoetin starting on day 0. Simulation results of WBC and HB are shown as black curve. Grey curves show predictions of dynamics without additional Darbepoetin applications. Median of data is represented by circles, and grey hatched line describe first and third quartile of the data [49,63].

BEACOPP escalated is an intense chemotherapy for the treatment of Hodgkin’s lymphoma resulting in high degrees of toxicity affecting all haematopoietic lineages [49,65]. G-CSF but not EPO support is mandatory. In consequence, anaemia due to cumulative toxicity to erythropoiesis is common at later cycles of the therapy. Here we simulate the effect of concomitant G-CSF and EPO applications during the course of the therapy. We simulate the application of eight cycles BEACOPP-21 escalated chemotherapy with Filgrastim on days 8–15 with or without weekly application of 300 IU/kg Darbepoetin (see Figure 12, left). While WBC dynamics are roughly the same, the decrease of HB is delayed by EPO possibly resulting in better quality of life of patients.

We also analysed the effect of weekly injections of 300 IU/kg Darbepoetin during eight cycles of CHOP-14 chemotherapy in elderly patients. Here, Filgrastim was applied on days 6–12 during each cycle of therapy. While dynamics of WBC are similar, HB stabilises at higher values during treatment with Darbepoetin.

## Discussion

Conventional cytotoxic chemotherapy plays a major role in cancer therapy. Development of intensified regimen improved the outcome of several diseases [45,46,49,66,67] but is limited by toxic side effects. A major dose-limiting side effect is general haematotoxicity which is routinely treated with growth factors EPO and G-CSF. Different pharmaceutical derivatives of these factors are available, which differ greatly in pharmacokinetic and -dynamic properties. Furthermore, outcome of growth factor treatment depends on many factors such as chemotherapy drugs used, drug doses, growth-factor derivatives and individual risk factors [68,69]. Due to this variety of variable therapy parameters, identification of optimal growth-factor schedules cannot be performed solely on the basis of clinical trials. We showed in the past that mathematical models of haematopoiesis under chemotherapy can facilitate the development of optimised and individualised growth-factor schedules [6].

Efforts to model haematopoiesis under chemotherapy and growth-factor applications are considerable [2,4,11-13,16,23,31,32,70-84]. All these attempts are based on mechanistic or semi-mechanistic parameterical models. In view of the complexity of the haematopoietic system, it might be worthwhile to consider model-free forecasts of haematopoietic outcomes (see discussion in [85,86]). However, to our knowledge, such an approach has never been proposed.

There are only a few attempts to develop multilineage models or hybrids of existing model: In [87] a compartmental model of granulopoiesis and thrombopoiesis including G-CSF and high dose chemotherapy application is proposed. Feedback mechanisms from thrombopoietic, granulopoietic and stem cells on stem cells and an interaction between the G-CSF compartment and the white blood cells are included. Erythropoesis was not considered. A stochastic multilineage model only considering haematopoietic stem cells and progenitor cells based on the idea of lineage commitment can be found in [42]. In [88] an ordinary differential equations model of granulopoiesis under chemotherapy and Filgrastim injections was combined with a model of stem cell plasticity. G-CSF and chemotherapy effects are modelled but erythropoiesis was not included. In the recent version of our erythropoeisis model, we combined a cell kinetic model with EPO injection models, with an EPO pharmacokinetic model and with a chemotherapy model developed for granulopoiesis. But granulopoiesis was not considered. In [89,90] several earlier models of haematopoietic stem stell dynamics and models for the regulation of neutrophils, platelets and erythrocytes were combined to a model of the regulation of the hematopoietic system describing periodic chronic myelogenous leukemia and cyclical neutropenia taking G-CSF effects into account. Chemotherapy was not considered in these models.

Here, we propose a comprehensive combined model of human erythropoiesis and granulopoiesis under chemotherapy, G-CSF and EPO applications for the first time. For this purpose, we combined a former cell kinetic model of erythropoiesis under chemotherapy and EPO applications developed by our group [10-13,16] with a cell kinetic model of granulopoiesis under chemotherapy and G-CSF applications [3,4,12,23]. These models were chosen for their clinically relevant applications and parameter settings established on the basis of large clinical datasets. Models are based on an ordinary differential equations system describing major cell stages in bone marrow and blood as well as dynamics of key cytokines. Rather than constructing a combined model from scratch we rely on these established modeling works and adopted their assumptions, equations and parameter settings as far as possible. Therefore, this approach can be considered as a second level of modeling by the combination of established models in order to build more comprehensive ones.

Accordingly, only a few additional assumptions and only a few new parameters were introduced in order to combine the model. The most challenging issue was to combine the models at stem cell level since regulation of stem cells dynamics depend on mature cell stages which was modelled differently in our single lineage models. This was solved by substituting the stem cell regulation by a model originally developed by Loeffler and Wichmann [12]. The model includes elements of lineage competition with respect to stem cell differentiation. Evidences of interactions between cell lineages regarding stem cell committment are summarised in [7]. Since model behaviour depends heavily on stem cell dynamics, it was by far not clear whether this approach would be successful. Indeed, this adaptation later required re-estimations of toxicity parameters of chemotherapy since stem cell toxicities are highly sensitive with respect to model outcome.

A second assumption was made with respect to suppression of erythropoiesis by G-CSF: Previous experiments have shown, that G-CSF administration leads to a reduction of CFU-E in the femur of mice [8,91-93]. Bungart et al. measured a decline of bone marrow CFU-E in mice to about % of the control value [91]. Bensinger et al. detected a greater decrease of hematocrit values in human blood donors receiving rhG-CSF than in donors without G-CSF [9]. However, the groups with or without G-CSF were not comparable. In view of these observations, we assumed that G-CSF decreases proliferation and prolonges transition time in CE [43]. However, our model simulations show that assuming a delay of the transition time in dependance on G-CSF dose and derivative is sufficient to describe the phenomena observed in human studies. Generally, suppression of erythropoiesis by G-CSF becomes apparent only under high serum levels of G-CSF over a longer time scale such as after Pegfilgrastim injections.

In summary, the major achievement of our present modelling efforts is that we successfully combined our single lineage models of erythropoiesis and granulopoiesis under chemotherapy by introducing a common stem cell model and an interaction of the lineages mediated by G-CSF. The model is mechanistic in the sense that it directly describes amplification and maturation processes of cell stages, relevant cell fluxes and PK/PD of EPO and G-CSF derivatives. However, we also have to acknowledge that our model is semi-mechanistic with respect to stem cell feedback and chemotherapy action which both are only phenomenologically described. This constitutes possible starting points for future model improvements.

While we rely on the parameter estimates of the single lineage models in general, newly introduced parameters based on assumptions necessary to combine the model were determined by fitting the predictions of the model to clinical data. Additionally, as mentioned earlier, it was also necessary to re-estimate toxicity parameters of chemotherapeutic drugs due to the changes required for stem cell modelling. It was our objective to construct a model predicting medians of patients, i.e. patients heterogeneity was not considered so far. We considered virtually all available time series data of mature blood parameters and cytokine levels after application of G-CSF, EPO, chemotherapy and combinations of it in order to parametrise the model or to validate its results. Data were retrieved either from the literature or from our own clinical study groups for which we have access to raw data of patients. This results in more than 100 scenarios comprising 15 different chemotherapeutic regimens.

In order to compare the results of our hybrid model with those of our single lineage models, we performed a number of qualitative simulations, namely single applications of G-CSF, EPO or chemotherapy. We observed some differences with respect to the dynamics of stem cells and earlier bone marrow compartments. In general, corresponding oscillations in the hybrid model are stronger and less damped compared to the single lineage models. However, this effect is only predicted for earlier cell stages for which no data are available. Due to additional regulations by G-CSF, the dynamics at later cell stages are almost identical to those of the former version of the model showing less pronounced oscillations at stem cell level. A similar observation was made by combining a two-dimensional difference equation model of stem cell regulation with our granulopoiesis model [88]. Thus, one can conclude that dynamics of blood cells are less informative regarding stem cell dynamics, i.e. appropriateness of different stem cell models can hardly be decided on the basis of available clinical data.

With the hybrid model we simulated more than 100 scenarios including growth factor applications in healthy volunteers, chemotherapy without growth factor application and chemotherapy with supportive G-CSF, EPO or both. Although there is a considerable inhomogeneity between patient groups considered (different age groups, diseases, distributions of risk factors) a good agreement between model simulation and data was achieved for almost all scenarios using a single parameter set (except for different toxicity parameters used for young and elderly patients). However, one has to acknowledge that the dynamics of white blood cells during chemotherapy is richer, and thus, more informative regarding appropriateness of model behaviour than the dynamics of the erythropoietic lineage. Reticulocyte data are scarcely available while erythrocytes, haematocrit or haemoglobin show more or less a constant decline in the time course of the therapy.

The majority of available data sets were used for model validation rather than parameter estimation. This especially applies for data after growth factor application into healthy volunteers for which no additional toxicity parameters are required. Additionally, a number of chemotherapy scenarios for which toxicity parameters were available from earlier modelling steps, were also used for model validation. The good agreement of model and data for almost all validation scenarios shows that the model covers a wide range of scenarios. Unfortunately, often data of only one of the lineages are available from the literature. Data received from our clinical trials are most detailed resulting in close meshed time series at a daily scale supported by dozens of single measurements. However, only data of leukocyte and haemoglobin are available to support our modelling. It would greatly benefit from more detailed blood parameters measured in parallel such as erythrocytes, hematocrit, hemoglobin, reticulocytes, iron status, and lymphocytes, granulocytes rather than leukocytes.

Finally, we demonstrated how the model could be used to make clinically relevant predictions regarding the outcome of different growth-factor schedules after chemotherapy. This requires that the toxcity parameters of the therapy are available from previous fitting steps. Then, the model can be used to simulate and compare alternative growth-factor schedules. We predict for example that both, patients treated with CHOP 14 or BEACOPP escalated therapy, would benefit from weekly Darbepoetin treatment without reducing the effectiveness of concomittant G-CSF treatment. Clinical trials are required to validate these model predictions.

However, we have to acknowledge that the present model only allows median predictions while critical time-courses are clinically more relevant and therapy-limiting. Although this aspect is not yet covered, there is a clear perspective towards modelling individual data either by fitting parameter sets for patient risk groups or by assuming distributions of model parameters. Accordingly, we plan to extend our model in the near future and apply it in order to support improvement and individualisation of growth factor therapies.

## Conclusions

We successfully combined established bio-mathematical models of granulopiesis and erythropoiesis under chemotherapy and growth-factor applications by introducing a common stem cell compartment and lineage interactions. The model explains data of about 100 clinical scenarios including 15 different chemotherapies and six growth-factor derivatives. We demonstrated how the model can be used to make clinically relevant predictions regarding combined G-CSF and EPO treatment during chemotherapy.

## Competing interests

The authors declare that they have no competing interests.

## Authors’ contributions

Model development: SS, MS Parameter estimation and model simulations: SS Paper writing: SS, MS All authors contributed to the discussion and the paper writing. All authors read and approved the final manuscript.

## Supplementary Material

Additional file 1 **A combined model of human erythropoiesis and granulopoiesis under growth factor and chemotherapy treatment: Supplement material.** The file ERYGRAsupp.pdf contains major model variables and mechanisms of the cell kinetic model. We provide all equations and parameters necessary to run the model. Additional simulation results are also included. Click here for file
