# Supplementary material for: A combined model of human erythropoiesis and granulopoiesis under growth factor and chemotherapy treatment
Source: Theor Biol Med Model. 2014 May 26;11:24. doi: 10.1186/1742-4682-11-24 (PMC4046020; doi:10.1186/1742-4682-11-24)
Supplement: Additional file 1 — A combined model of human erythropoiesis and granulopoiesis under growth factor and chemotherapy treatment: Supplement material. The file ERYGRAsupp.pdf contains major model variables and mechanisms of the cell kinetic model. We provide all equations and parameters necessary to run the model. Additional simulation results are also included. [file 1742-4682-11-24-S1.pdf]

A combined model of human erythropoiesis and granulopoiesis under growth factor and chemotherapy treatment: Supplement material

Sibylle Schirm[1,2] , Christoph Engel[1] , Markus Loeffler[1] and Markus Scholz*[1,2]

[1]Institute for Medical Informatics, Statistics and Epidemiology, University of Leipzig, Leipzig, Germany
[2]LIFE Research Center of Civilization Diseases, University of Leipzig, Leipzig, Germany

Email: sibylle.schirm@imise.uni-leipzig.de; christoph.engel@imise.uni-leipzig.de; markus.loeffler@imise.uni-leipzig.de; markus.scholz@imise.uni-leipzig.de;

*Corresponding author

A Model Equations

Here we describe all major model variables, mechanisms and equations of the cell kinetic model. An overview of model variables and parameters is shown in table A.1. At first, we describe major model principles and corresponding equations.

A.1 Amplification Splitting

Influx and efflux of cells at one compartment are amplified, so that the product is the over-all amplification ($A_X^{\mathrm{in}}(t) \cdot A_X^{\mathrm{out}}(t) = A_X(t)$),

$$A_X^{\mathrm{in}}(t) = \begin{cases} \frac{A_X - 1}{\mathrm{ld} A_X} & \text{for} \quad A_X \neq 0, A_X \neq 1 \\ \ln 2 \quad \text{for} & A_X \neq 1 \\ 0 \quad \text{for} & A_X = 0 \end{cases} \tag{A.1}$$

$$A_X^{\mathrm{out}}(t) = \begin{cases} \frac{A_X}{A_X^{\mathrm{in}}} & \text{for} \quad A_X \neq 0 \\ 0 \quad \text{for} & A_X = 0 \end{cases} . \tag{A.2}$$

The effect is a delayed reaction of efflux and compartment size to changes in amplification rates. Amplification splitting is modelled for all compartments with amplification.

Table A.1: variables

quantity	meaning	type/calculation
C_X	content of compartment X function of time t	
C_X^{nor}	content of compartment X in steady state (normal value)	parameter, in general we set $C_X(0) = C_X^{\text{nor}}$
C_X^{rel}	content of compartments X relative to normal value	$C_X^{\text{rel}}(t) = \frac{C_X(t)}{C_X^{\text{nor}}}$
C_X^{in}	influx in compartment X	function of time
$C_X^{\text{in\_nor}}$	normal influx	parameter, see above
C_X^{out}	efflux from compartment X	function of time
$C_X^{\text{out\_nor}}$	normal efflux	parameter, see above
a_X	proliferative fraction in cell compartment X	function of state, sometimes constant
A_X	amplification in cell compartment X	"
A_X^{in}	amplification of influx	"
A_X^{in}	amplification of effflux	"
n_X	average number of mitoses in cell compartment X	$n_X = ldA_X$
p	self-renewal probability of stem cells	function of state
τ_X	average duration of cell cycle in compartment X	function of time, sometimes constant (not regulated)
T_X	average transit time of active cells in cell compartment X	$T_X = n_X \tau_X$
T_X^t	total transit time	$T_X^t = \frac{n_X \tau_X}{a_X}$
k	transition, degradation or toxicity coefficients	functions of time or parameter
$Y^{\min}$	quantity Y under minimum stimulation	parameter to determine the regulatory function of Y
Y^{nor}	quantity Y in steady state	"
Y^{int}	quantity Y under intensified stimulation	"
$Y^{\max}$	quantify Y under maximum stimulation	"
b_Y	sensitivity of Y under stimulation	"

A.2 Regulatory Function

Amplification A and transition time T are regulated between a minimum $Y^{\min}$ and a maximum $Y^{\max}$ by the growth factors EPO or G-CSF according to the following *regulatory function*:

$$Y = Z_Y\big(C_{\text{Cyto}}(t); Y^{\min}, Y^{\text{nor}}, Y^{\max}, b_Y\big) \tag{A.3}$$

$$Z_Y\big(C_{\text{Cyto}}\big) = \begin{cases} Y^{\max} - (Y^{\max} - Y^{\min}) e^{-\ln\left(\frac{Y^{\max} - Y^{\min}}{Y^{\max} - Y^{\text{nor}}}\right)\left(C_{\text{Cyto}}\right)^{b_Y}} \\ \text{for} \quad Y^{\min} < Y^{\text{nor}} < Y^{\max} \text{ or } Y^{\max} < Y^{\text{nor}} < Y^{\min} \\ Y^{\text{nor}} \quad \text{for} \quad Y^{\min} < Y^{\text{nor}} < Y^{\max}, \end{cases} \tag{A.4}$$

where Y^{nor} is the steady state value of amplification or transition time, $C_{\text{Cyto}} \in \{C_{\text{EPO}}^{\text{int}}, C_{\text{GCSF}}^{\text{rel}}\}$ and b_Y is the sensitivity of Y under stimulation (see figure A.2 and [1], p. 69).

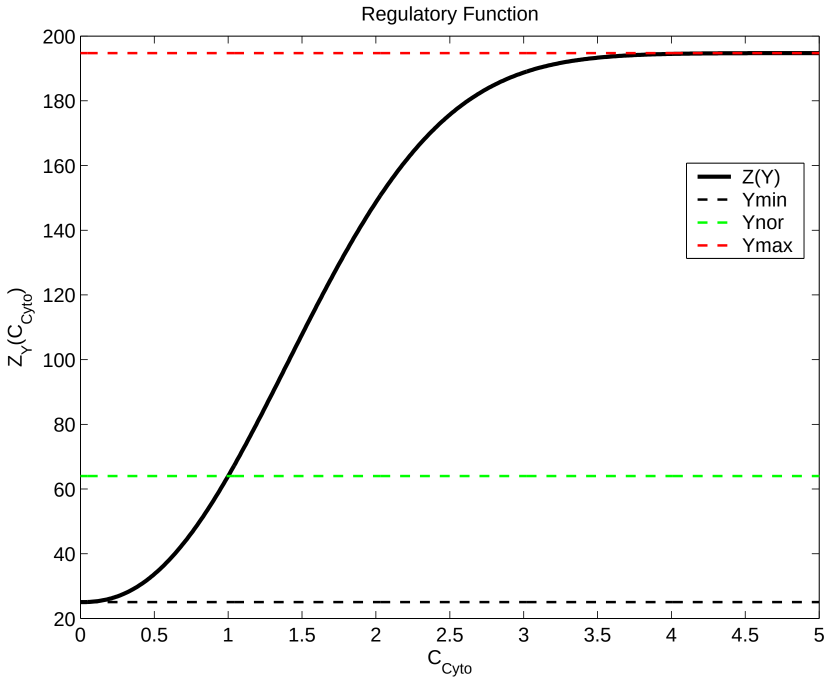

Figure 1: **Regulatory function.** As an example we plot the regulatory function of the amplification in compartment BE, C_{Cyto} = EPO concentration $C_{\text{EPO}}^{\text{int}}$.

A.3 Modelling of Delays

Several delays are included into the model such as delayed action of growth factors or chemotherapy. According to [2,3], these delays are modelled by a set of concatenated compartments with first order transitions

$$\frac{d}{dt}C_X^1 = C_X - D_X C_X^1$$
$$\frac{d}{dt}C_X^i = D_X \left(C_X^{i-1} - C_X^i\right) \quad i = 2, \ldots, N_X$$

where C_X is a quantity to be delayed, D_X is the delay parameter and N_X is the number of delay compartments. The delayed quantity is now defined by

$$C_X^{\text{del}} = D_X C_X^{N_X}$$

i.e. as the efflux from the last delay compartment.

As explained in [2,3] this modelling of delays is in between a random, age-independent transition ($N_X = 1$) and a strict "first-in-first-out" kinetic ($N_X \to \infty$). By our modelling, one can mimic both, an expectation and a variance of individual delay times. This is achieved by setting $D_X = T_X/N_X$ where T_X is the desired expectation of the transition time and N_X determines its variance (see [2] for details).

A.4 Chemotherapy

The infusion of chemotherapeutic drugs is modelled by Heaviside functions

$$\mathrm{CHEMO}^{\mathrm{drug}}(t) = \sum_{i=1}^{N^{\mathrm{cycle}}} \left(\mathrm{Hv}(t-\tilde{t_i}) - \mathrm{Hv}(t-\tilde{t_i}-t_{\mathrm{inf}}^{\mathrm{CHEMO}})\right), \tag{A.5}$$

where N^{cycle} is the number of chemotherapy cycles, $\tilde{t_i}$ are the time points with application of a specific drug, and $t_{\mathrm{inf}}^{\mathrm{CHEMO}}$ is the duration of chemotherapy application. The delayed effect of the drugs is modelled by four compartments

$$\frac{d\Psi_{\mathrm{drug}}^{(i)}(t)}{dt} = \Psi_{\mathrm{drug\_out}}^{(i-1)}(t) - k_{\mathrm{Delay}}^{\mathrm{drug}} \cdot \Psi_{\mathrm{drug}}^{(i)}(t), \quad i = 1, \ldots, 4, \tag{A.6}$$

with

$$\Psi_{\mathrm{drug}}^{(0)}(t) = \mathrm{CHEMO}^{\mathrm{drug}}(t),$$
$$\Psi_{\mathrm{drug\_out}}^{(i)}(t) = k_{\mathrm{Delay}}^{\mathrm{drug}} \cdot \Psi_{\mathrm{drug}}^{(i)}(t).$$

The output-function $\Psi_{\mathrm{drug\_out}}^{(4)}(t)$ is multiplied by the toxicity parameters of the single compartments $k_{\mathrm{S}}^{\mathrm{drug}}$, $k_{\mathrm{CG}}^{\mathrm{drug}}$, $k_{\mathrm{PGB}}^{\mathrm{drug}}$, $k_{\mathrm{MGB}}^{\mathrm{drug}}$, $k_{\mathrm{BE}}^{\mathrm{drug}}$, $k_{\mathrm{CE}}^{\mathrm{drug}}$, $k_{\mathrm{PEB}}^{\mathrm{drug}}$, $k_{\mathrm{MEB}}^{\mathrm{drug}}$, and $k_{\mathrm{RET}}^{\mathrm{drug}}$ respectively. If muliple cytotoxic drugs are applied, corresponding toxicity functions are added resulting in an overall toxic effect Ψ_X which is cell stage and chemotherapy specific. In complete analogy to our former work [3,4], the effect of chemotherapy is introduced to the balance equations of the bone marrow cell compartments by a first-order loss term. Hence, the schematic compartment equation has the form

$$\frac{d}{dt}C_X = A^{\mathrm{in}} \cdot C_X^{\mathrm{in}} - \frac{C_X}{T} - \Psi_X \cdot C_X.$$

In clinical practice often only leukocytes are available. We calculate the leukocyte count as the sum of lymphocytes and granulocytes. To avoid a full model of lymphopoiesis, we modelled the reduced lymphocyte count under chemotherapy by an exponential function of the corresponding toxicity function.

$$C_{\mathrm{WBC}}(t) \quad \approx \quad c_{\mathrm{LY}} \exp\left(-\Psi^{\mathrm{LY}}(t)\right) + c_{\mathrm{GRA}} \frac{C_{\mathrm{GRA}}(t)}{C_{\mathrm{GRA}}^{\mathrm{nor}}} \tag{A.7}$$

where $c_{\mathrm{LY}} = 3000$ cells per μl and $c_{\mathrm{GRA}} = 4000$ cells per μl are the normal concentrations of lymphocytes and granulocytes respectively. Ψ_{LY} is the toxicity function for lymphocytes defined in analogy to the toxicity functions of bone marrow cell stages (see above).

A.5 Stem cell compartment S

Cells differentiating into granulopoietic or erythropoietic lineages originate from the same stem cell compartment. We adopted the corresponding stem cell model of *Loeffler & Wichmann* [1]. The output of the stem cell compartment is splitted into red or white cell lines under the assumption that 15% of the released cells differentiate into red blood cells ($\alpha_E = 0.15$), and 80% into the white blood cell line ($\alpha_G = 0.8$). The stem cell compartment S has self-renewal capability. Under steady state conditions, 50% of the stem cells remain in this compartment, and 50% differentiate into red or white blood cell lineages. Hence, the stem cell compartment equation is

$$\frac{d}{dt}C_S = (2p-1)C_S\frac{a_S}{\tau_S} \tag{A.8}$$

$$C_S^{\text{out}} = 2(1-p)C_S\frac{a_S}{\tau_S}, \tag{A.9}$$

where a_S is the proliferative fraction, τ_S is the average duration of a cell cycle, and p the self-renewal probability of stem cells.

The self renewal probability p is regulated by a competition of the stem cells ($C_S^{\text{rel}}(t)$), the granulopoietic ($C_G^{\text{rel}}(t)$), and the erythropoietic ($C_E^{\text{rel}}(t)$) bone marrow cells:

$$p = p\big(C_S^{\text{rel}}(t), C_E^{\text{rel}}(t), C_G^{\text{rel}}(t), p_\delta, \vartheta_E, \vartheta_G, \vartheta_S(t)\big),$$

where the parameters $\vartheta_E = -2, \vartheta_G = -8$, and $\vartheta_S(t)$ are hypothetical weighting factors representing the strength of the influence of the bone marrow cells $C_S^{\text{rel}}(t) = \frac{C_S(t)}{C_S^{\text{nor}}}$, $C_E^{\text{rel}}(t) = \frac{C_{\text{BE}}(t)+C_{\text{CE}}(t)+C_{\text{PEB}}(t)+C_{\text{MEB}}(t)}{C_{\text{BE}}^{\text{nor}}+C_{\text{CE}}^{\text{nor}}+C_{\text{PEB}}^{\text{nor}}+C_{\text{BE}}^{\text{nor}}}$, and $C_G^{\text{rel}}(t) = \frac{C_{\text{CG}}(t)+C_{\text{PGB}}(t)+C_{\text{MGB}}}{C_{\text{CG}}^{\text{nor}}+C_{\text{PGB}}^{\text{nor}}+C_{\text{MGB}}^{\text{nor}}}$. It is assumed that

$$p_\delta = p^{\text{nor}} - p^{\text{min}} = p^{\text{max}} - p^{\text{nor}}$$

$$\vartheta_S(t) = \begin{cases} \frac{2}{C_S^{\text{rel}}(t)^{0.6}} & \text{for } C_S^{\text{rel}}(t) \le 1 \\ 2 & \text{for } C_S^{\text{rel}}(t) > 1 \end{cases}$$

$$p = p_\delta \tanh\Big(-\vartheta_S(t)\big(C_S^{\text{rel}}(t)-1\big) - \vartheta_E\big(C_E^{\text{rel}}(t)-1\big) - \vartheta_G\big(C_G^{\text{rel}}(t)-1\big)\Big) + 0.5,$$

where the steady state value $p^{\text{nor}} = \frac{1}{2}$. Thus, for the initial conditions it holds that

$$C_S(0) = C_S^{\text{nor}} = 1 \tag{A.10}$$

$$C_S^{\text{out}}(0) = C_S^{\text{out\_nor}} = 2(1-p^{\text{nor}})C_S^{\text{nor}}\frac{a_S^{\text{nor}}}{\tau_S}. \tag{A.11}$$

The proliferative fraction a_S can be interpreted as the percentage of cells which are currently in cell cycle. The proliferative fractions a_X of the compartments S, BE and CG are regulated by the haematopoietic bone

marrow system $C_S^{\mathrm{rel}}(t)$ and $C_G^{\mathrm{rel}}(t)$ or $C_E^{\mathrm{rel}}(t)$:

$$a_X = a_X\big(C_S^{\mathrm{rel}}(t), C_E^{\mathrm{rel}}(t), C_G^{\mathrm{rel}}(t), a_X^{\min}, a_X^{\mathrm{nor}}, a_X^{\mathrm{int}}, a_X^{\max}, \omega_E, \omega_G, \omega_S\big).$$

The parameters $\omega_E = 0.3, \omega_G = 0.1$ and $\omega_S = 1$ are weighting factors. They represent the strengths of the influence of stem cells, erythropoietic and granulopoietic cells on the proliferative fraction of BE, CG and S. With

$$\begin{aligned} x =& \, \omega_E \ln C_E^{\mathrm{rel}}(t) + \omega_G \ln C_G^{\mathrm{rel}}(t) \\ &+ \omega_S \begin{cases} \ln C_S^{\mathrm{rel}}(t), & \text{for} \quad C_S^{\mathrm{rel}} \leq 1 \\ C_S^{\mathrm{rel}}(t) - 1, & \text{for} \quad C_S^{\mathrm{rel}} > 1 \end{cases} \\ y =& -\frac{1}{2\ln 2}\left(\ln\left(\frac{a_X^{\mathrm{int}} - a_X^{\max}}{a_X^{\min} - a_X^{\mathrm{int}}}\right) - \ln\left(\frac{a_X^{\mathrm{nor}} - a_X^{\max}}{a_X^{\min} - a_X^{\mathrm{nor}}}\right)\right) x \\ &+ \frac{1}{2}\ln\left(\frac{a_X^{\mathrm{nor}} - a_X^{\max}}{a_X^{\min} - a_X^{\mathrm{nor}}}\right), \end{aligned} \tag{A.12}$$

the proliferative fraction is given by

$$a_X = \begin{cases} \frac{a_X^{\max} e^{-y} + a_X^{\min} e^{y}}{e^{-y} + e^{y}} & \text{for} \quad a_X^{\min} < a_X^{\mathrm{nor}} < a_X^{\mathrm{int}} < a_X^{\max} \\ a_X^{\mathrm{nor}} & \text{for} \quad a_X^{\min} = a_X^{\mathrm{nor}} = a_X^{\mathrm{int}} = a_X^{\max} \end{cases},$$

where X is one of the compartments S, BE, or CG. It is a monotone function with ranges between $a_X^{\min}$ and $a_X^{\max}$. Low cell numbers in the bone marrow compartments cause a higher demand of proliferating cells and therefore a larger proliferative fraction a_X. The value of y defines the actual point on the regulatory curve. The variable

x is a measure of the total bone marrow content. It is calculated as a weighted sum of the logarithms of the relative counts of stem cells, erythropoietic cells and granulopoietic cells. If any cell counts tend to zero, x tends to minus infinity, and with it, a becomes maximal. Parameter values a^{int} corresponds to $x = -\ln 2$ and a^{nor} corresponds to $x = 0$ (see figure 2).

A.6 Erythropoiesis

Compartment BE

The model equations of BE are similar to [4], but now the additional influence of the granulopoietic cell lineage on the proliferative fraction in BE is taken into account.

$$\begin{aligned} \frac{d}{dt} C_{\mathrm{BE}} &= \alpha_E C_S^{\mathrm{out}} A_{\mathrm{BE}}^{\mathrm{in}} - C_{\mathrm{BE}} \frac{a_{\mathrm{BE}}}{T_{\mathrm{BE}}} - \Psi_{\mathrm{BE}} \cdot C_{\mathrm{BE}} \\ C_{\mathrm{BE}}^{\mathrm{out}} &= C_{\mathrm{BE}} A_{\mathrm{BE}}^{\mathrm{out}} \frac{a_{\mathrm{BE}}}{T_{\mathrm{BE}}} \end{aligned}$$

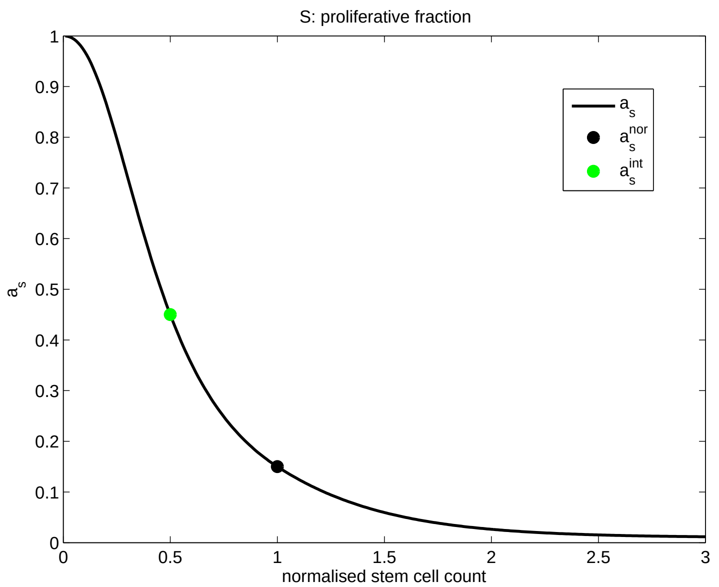

Figure 2: **Regulation of the proliferative fraction - dependence on normalised stem cell count.** Cell stages C_E and C_G were set to normal values. The graph has the same shape if these quantities are varied instead of C_S. Parameter settings: $a_S^{\min} = 0.01, a_S^{\text{nor}} = 0.15, a_S^{\text{int}} = 0.45, a_S^{\max} = 1$.

with the initial values

$$C_{\text{BE}}(0) = C_{\text{BE}}^{\text{nor}} = \alpha_E C_S^{\text{out\_nor}} A_{\text{BE}}^{\text{in\_nor}} \frac{T_{\text{BE}}^{\text{nor}}}{a_{\text{BE}}^{\text{nor}}}$$
$$C_{\text{BE}}^{\text{out\_nor}} = C_{\text{BE}}^{\text{nor}} A_{\text{BE}}^{\text{out\_nor}} \frac{a_{\text{BE}}^{\text{nor}}}{T_{\text{BE}}^{\text{nor}}} = \alpha_E C_S^{\text{out\_nor}} A_{\text{BE}}^{\text{nor}},$$

where $\alpha_E = 0.15$ is the proportion of cells differentiating into erythropoietic cell lineage [1].

Compartment CE

$$A_{\text{CE}} = Z(C_{\text{EPO}}^{\text{int}})$$
$$\frac{d}{dt} C_{\text{CE}} = C_{\text{BE}}^{\text{out}} A_{\text{CE}}^{\text{in}} - \frac{C_{\text{CE}}}{T_{\text{CE}}} - \Psi_{\text{CE}} \cdot C_{\text{CE}}$$
$$C_{\text{CE}}^{\text{out}} = C_{\text{CE}} A_{\text{CE}}^{\text{out}} \frac{a_{\text{CE}}}{T_{\text{CE}}}$$

The initial values are

$$C_{\text{CE}}(0) = C_{\text{CE}}^{\text{nor}} = C_{\text{BE}}^{\text{out\_nor}} A_{\text{CE}}^{\text{in\_nor}} T_{\text{CE}}^{\text{nor}}$$
$$C_{\text{CE}}^{\text{out}}(0) = C_{\text{CE}}^{\text{out\_nor}} = C_{\text{BE}}^{\text{out\_nor}} A_{\text{CE}}^{\text{nor}}.$$

The amplification and transit time in the compartment CE are regulated by the growth factors EPO and G-CSF. This is modelled by regulatory functions acting on proliferation rate and transition time in the

compartment CE. The regulatory function of amplification is regulated by the internalised EPO $Z_{A_{\text{CE}}}\big(C_{\text{EPO}}^{\text{int}}\big)$. The regulatory function of the transition time is also regulated by internalised EPO, $Z_{T_{\text{CE}}}\big(C_{\text{EPO}}^{\text{int}}\big)$. But, it is additionally multiplied by a regulatory function regulated by G-CSF ($F_{\text{GCSF}}(t)$), mimicking the G-CSF effect on CE. In summary, amplification and transition time in CE are calculated by

$$A_{\text{CE}} = Z_{A_{\text{CE}}}\big(C_{\text{EPO}}^{\text{int}}\big) \tag{A.13}$$

$$T_{\text{CE}} = Z_{T_{\text{CE}}}\big(C_{\text{EPO}}^{\text{int}}\big) \cdot F_{\text{T\_GCSF}}(t). \tag{A.14}$$

with

$$F_{\text{T\_GCSF}}(t) = \omega_P(t) \cdot Z_{\text{T\_Peg}}(t) + (1 - \omega_P(t)) \cdot Z_{\text{T\_Fil}}(t), \tag{A.15}$$

where $Z_{\text{T\_Peg}}(t)$ and $Z_{\text{T\_Fil}}(t)$ are the regulation functions of the growth factors endogenous G-CSF and Filgrastim on one hand and Pegfilgrastim on the other hand (equation A.4), $0 \leq \omega_P(t) \leq 1$ is the weighting factor to model the superimposing effect of concurrent Filgrastim or endogenous G-CSF, and Pegfilgrastim (see [3]), and $C_{\text{EPO}}^{\text{int}}$ is the internalised EPO (see [4]):

$$\omega_P = Z_{\omega_P}\left(C_{\text{GCSF}}^{\text{cent\_rel\_del\_peg}} / C_{\text{GCSF}}^{\text{cent\_rel\_del\_fil}}\right) \quad \text{with} \quad \omega_P^{\min} = 0 \quad \text{and} \quad \omega_P^{\max} = 1 \tag{A.16}$$

Compartment PEB

$$A_{\text{PEB}} = Z(C_{\text{EPO}}^{\text{int}})$$
$$\frac{d}{dt}C_{\text{PEB}} = C_{\text{CE}}^{\text{out}} A_{\text{PEB}}^{\text{in}} - \frac{C_{\text{PEB}}}{T_{\text{PEB}}} - \Psi_{\text{PEB}} \cdot C_{\text{PEB}}$$
$$C_{\text{PEB}}^{\text{out}} = C_{\text{PEB}} \frac{A_{\text{PEB}}^{\text{out}}}{T_{\text{PEB}}}$$

The initial values are

$$C_{\text{PEB}}(0) = C_{\text{PEB}}^{\text{nor}} = C_{\text{CE}}^{\text{out\_nor}} A_{\text{PEB}}^{\text{in\_nor}} T_{\text{PEB}}^{\text{nor}}$$
$$C_{\text{PEB}}^{\text{out}}(0) = C_{\text{PEB}}^{\text{out\_nor}} = C_{\text{CE}}^{\text{out\_nor}} A_{\text{PEB}}^{\text{nor}}.$$

Compartment MEB

The maturation is modelled by splitting MEB into $N_{\mathrm{MEB}} = 15$ subcompartments, without amplification.

$$
\begin{aligned}
T_{\mathrm{MEB}} &= Z(C_{\mathrm{EPO}}^{\mathrm{int}}) \\
C_{\mathrm{MEB}} &= \sum_{i=1}^{N_{\mathrm{MEB}}} C_{\mathrm{MEB}_i} \\
\frac{d}{dt} C_{\mathrm{MEB}_1} &= C_{\mathrm{PEB}}^{\mathrm{out}} - C_{\mathrm{MEB}_1} \frac{N_{\mathrm{MEB}}}{T_{\mathrm{MEB}}} - \Psi_{\mathrm{MEB}} \cdot C_{\mathrm{MEB}_1} \\
\frac{d}{dt} C_{\mathrm{MEB}_i} &= C_{\mathrm{MEB}_{i-1}}^{\mathrm{out}} - C_{\mathrm{MEB}_i} \frac{N_{\mathrm{MEB}}}{T_{\mathrm{MEB}}} - \Psi_{\mathrm{MEB}} \cdot C_{\mathrm{MEB}_i}, \quad i = 2, \ldots, N_{\mathrm{MEB}} \\
C_{\mathrm{MEB}_i}^{\mathrm{out}} &= C_{\mathrm{MEB}_i} \frac{N_{\mathrm{MEB}}}{T_{\mathrm{MEB}}}, \quad i = 1, \ldots, N_{\mathrm{MEB}} \\
C_{\mathrm{MEB}}^{\mathrm{out}} &= C_{\mathrm{MEB}_{N_{\mathrm{MEB}}}}^{\mathrm{out}},
\end{aligned}
$$

The initial values are

$$
\begin{aligned}
C_{\mathrm{MEB}}(0) &= C_{\mathrm{MEB}}^{\mathrm{nor}} = C_{\mathrm{PEB}}^{\mathrm{out\_nor}} T_{\mathrm{MEB}}^{\mathrm{nor}} \\
C_{\mathrm{MEB}_i}(0) &= C_{\mathrm{MEB}_i}^{\mathrm{nor}} = C_{\mathrm{PEB}}^{\mathrm{out\_nor}} \frac{T_{\mathrm{MEB}}^{\mathrm{nor}}}{N_{\mathrm{MEB}}}, \quad i = 1, \ldots, N_{\mathrm{MEB}} \\
C_{\mathrm{MEB}_i}^{\mathrm{out}}(0) &= C_{\mathrm{MEB}_i}^{\mathrm{out\_nor}} = C_{\mathrm{MEB}_i}^{\mathrm{nor}} \frac{N_{\mathrm{MEB}}}{T_{\mathrm{MEB}}^{\mathrm{nor}}} = C_{\mathrm{PEB}}^{\mathrm{out\_nor}}, \quad i = 1, \ldots, N_{\mathrm{MEB}} \\
C_{\mathrm{MEB}}^{\mathrm{out}}(0) &= C_{\mathrm{MEB}}^{\mathrm{out\_nor}} = C_{\mathrm{MEB}_{N_{\mathrm{MEB}}}}^{\mathrm{out\_nor}} = C_{\mathrm{PEB}}^{\mathrm{out\_nor}}.
\end{aligned}
$$

Compartment RET

$$
\begin{aligned}
T_{\mathrm{RET}} &= T_{\mathrm{MEB}}^{\mathrm{nor}} + T_{\mathrm{RET}}^{\mathrm{nor}} - T_{\mathrm{MEB}} \\
\frac{d}{dt} C_{\mathrm{RET}} &= C_{\mathrm{MEB}}^{\mathrm{out}} - \frac{C_{\mathrm{RET}}}{T_{\mathrm{RET}}} - \Psi_{\mathrm{RET}} \cdot C_{\mathrm{RET}} \\
C_{\mathrm{RET}}^{\mathrm{out}} &= \frac{C_{\mathrm{RET}}}{T_{\mathrm{RET}}} \\
C_{\mathrm{RET}}(0) &= C_{\mathrm{RET}}^{\mathrm{nor}} = C_{\mathrm{ERY}}^{\mathrm{nor}} \frac{q_{\mathrm{RET}}}{1 - q_{\mathrm{RET}}} \\
C_{\mathrm{RET}}^{\mathrm{out}}(0) &= C_{\mathrm{RET}}^{\mathrm{out\_nor}} = C_{\mathrm{MEB}}^{\mathrm{out\_nor}} \\
T_{\mathrm{RET}}^{\mathrm{nor}} &= \frac{C_{\mathrm{RET}}^{\mathrm{nor}}}{C_{\mathrm{RET}}^{\mathrm{out\_nor}}} = \frac{q_{\mathrm{RET}}}{1 - q_{\mathrm{RET}}} \big((1 - s_{\mathrm{ERY}}^{\mathrm{nor}}) T_{\mathrm{ERY\_rnd}} + s_{\mathrm{ERY}}^{\mathrm{nor}} T_{\mathrm{ERY\_age}} \big).
\end{aligned}
$$

q_{RET} is the proportion of reticulocytes to the total number of red blood cells in steady state. $s_{\mathrm{ERY}}^{\mathrm{nor}}$, $T_{\mathrm{ERY\_rnd}}$, and $T_{\mathrm{ERY\_age}}$ were explained in the next section.

Compartment ERY

The compartment ERY is split into the compartments "RANDOM" and "AGE". In steady state, most erythrocytes die dependent on age. The age dependent reduction is modelled by divisions into subcompart-

ments.

Under stimulation, the depletion is more randomly (i.e. exponential decay, see [5]). Hence, the influxes into the compartments "RANDOM" and "AGE" are regulated by the factor s_{ERY}, which depends on the bone marrow output of the reticulocytes. $T_{\text{ERY\_rnd}}$, and $T_{\text{ERY\_age}}$ are transition times in the subcompartments "RANDOM" and "AGE". (See [1, 2, 5, 6].)

$$
\begin{aligned}
s_{\text{ERY}} &= \exp\left(\left(\frac{C_{\text{RET}}^{\text{out}}}{C_{\text{RET}}^{\text{out\_nor}}}\right)^2 \ln s_{\text{ERY}}^{\text{nor}}\right) \\
C_{\text{ERY}} &= C_{\text{ERY\_age}} + C_{\text{ERY\_rnd}} \\
C_{\text{ERY\_age}} &= \sum_{i=1}^{N_{\text{ERY}}} C_{\text{ERY\_age\_}i} \\
\frac{d}{dt} C_{\text{ERY\_age\_1}} &= s_{\text{ERY}} C_{\text{RET}}^{\text{out}} - C_{\text{ERY\_age\_1}} \frac{N_{\text{ERY}}}{T_{\text{ERY\_age}}}
\end{aligned}
$$

$$
\begin{aligned}
\frac{d}{dt} C_{\text{ERY\_age\_}i} &= C_{\text{ERY\_age\_}i-1}^{\text{out}} - C_{\text{ERY\_age\_}i}^{\text{out}}, \quad i = 2, \ldots, N_{\text{ERY}} \\
C_{\text{ERY\_age\_}i}^{\text{out}} &= C_{\text{ERY\_age\_}i} \frac{N_{\text{ERY}}}{T_{\text{ERY\_age}}} \\
\frac{d}{dt} C_{\text{ERY\_rnd}} &= (1 - s_{\text{ERY}}) C_{\text{RET}}^{\text{out}} - C_{\text{ERY\_rnd}} \frac{1}{T_{\text{ERY\_rnd}}},
\end{aligned}
$$

with initial conditions

$$
\begin{aligned}
C_{\text{ERY}}(0) &= C_{\text{ERY}}^{\text{nor}} = C_{\text{ERY\_age}}^{\text{nor}} + C_{\text{ERY\_rnd}}^{\text{nor}} \\
C_{\text{ERY\_age}}(0) &= C_{\text{ERY\_age}}^{\text{nor}} = \sum_{i=1}^{N_{\text{ERY}}} C_{\text{ERY\_age\_}i}^{\text{nor}} = s_{\text{ERY}}^{\text{nor}} C_{\text{RET}}^{\text{out\_nor}} T_{\text{ERY\_age}} \\
C_{\text{ERY\_age\_1}}(0) &= C_{\text{ERY\_age\_1}}^{\text{nor}} = s_{\text{ERY}}^{\text{nor}} C_{\text{RET}}^{\text{out\_nor}} \frac{T_{\text{ERY\_age}}}{N_{\text{ERY}}} \\
C_{\text{ERY\_age\_}i}(0) &= C_{\text{ERY\_age\_}i}^{\text{nor}} = C_{\text{ERY\_age\_}i-1}^{\text{out\_nor}} \frac{T_{\text{ERY\_age}}}{N_{\text{ERY}}}, \quad i = 2, \ldots, N_{\text{ERY}} \\
&= s_{\text{ERY}}^{\text{nor}} C_{\text{RET}}^{\text{out\_nor}} \frac{T_{\text{ERY\_age}}}{N_{\text{ERY}}}, \quad i = 1, \ldots, N_{\text{ERY}} \\
C_{\text{ERY\_age\_}i}^{\text{out}}(0) &= C_{\text{ERY\_age\_}i}^{\text{out\_nor}} = C_{\text{ERY\_age\_}i}^{\text{nor}} \frac{N_{\text{ERY}}}{T_{\text{ERY\_age}}} = s_{\text{ERY}}^{\text{nor}} C_{\text{RET}}^{\text{out\_nor}} \\
C_{\text{ERY}_{\text{rnd}}}(0) &= C_{\text{ERY\_rnd}}^{\text{nor}} = (1 - s_{\text{ERY}}^{\text{nor}}) C_{\text{RET}}^{\text{out\_nor}} T_{\text{ERY}_{\text{rnd}}}
\end{aligned}
$$

Endogenous production of EPO

According to [6, 7], the endogenous production of EPO (EPO_{prod}) is assumed to depend on the oxygen partial pressure in the kidneys and the number of circulating red blood cells

$$
\begin{aligned}
\text{EPO}_{\text{prod}} &= P_{max}^{\text{endo}} \cdot e^{-b_{\text{EPO}} \cdot f}, \quad \text{where} \quad \text{EPO}_{\text{prod}}(0) = 1 \quad \text{and} \quad f = \frac{P_{O_2}^t}{P_{O_2}^{t\_\text{nor}}} \\
P_{O_2}^t &= P_{50} \cdot \left(\frac{S_{O_2}^t}{100 - S_{O_2}^t} \right)^{\frac{1}{\gamma}} \text{(Hill equation)} \\
S_{O_2}^t &= \frac{100}{\left(\frac{P_{50}}{P_{O_2}^{A\_\text{nor}}} \right)^{\gamma} + 1} - \Delta SO_2 \cdot \frac{RET^{\text{nor}} + ERY^{\text{nor}}}{C_{\text{RET}} + C_{\text{ERY}}} \\
S_{O_2}^{t\_\text{nor}} &= \frac{100}{\left(\frac{P_{50}}{P_{O_2}^{A\_\text{nor}}} \right)^{\gamma} + 1} - \Delta SO_2 \\
P_{O_2}^{t\_\text{nor}} &= P_{50} \cdot \left(\frac{S_{O_2}^{t\_\text{nor}}}{100 - S_{O_2}^{t\_\text{nor}}} \right)^{\frac{1}{\gamma}}
\end{aligned}
$$

The variables are explained in Table A.2. For further explanation and justification, see [4, 6, 7].

Table A.2: Variables for endogenous EPO production

quantity	meaning	type/calculation	
$P_{O_2}^t$	kidney tissue oxygen tension	function of time	[6, 7]
$P_{O_2}^{t\_\text{nor}}$	kidney tissue oxygen pressure, normal value		[6, 7]
$S_{O_2}^t$	percent saturation of hemoglobin	function of time	[6, 7]
$S_{O_2}^{t\_\text{nor}}$	normal value of percent saturation of hemoglobin	constant	[6, 7]
P_{50}	partial oxygen pressure corresponding to $S_{O_2}^t = 50\%$	26.5 mm Hg	[6, 7]
$P_{O_2}^{A\_\text{nor}}$	arterial oxygen pressure, normal value	97 mm Hg	[6, 7]
ΔSO_2	desaturation of HB (arteriovenous difference), normal value	20 %	[6, 7]
γ	Hills coefficient, describes the slope of the curve	2.65	[6, 7]
P_{max}^{endo}	maximum production	200 (set)	[6, 7]
b_{EPO}	sensitivity of EPO_{prod} to changes in $P_{O_2}^t$	ln 200 (set)	[6, 7]

Exogenous EPO Application

In our model, we use simple pulse functions EPO^{inj} for intravenous injections. Regarding subcutaneous injections, the model adapted from [8] includes direct absorption from the subcutaneous tissue into the bloodstream, or indirect through the lymphatic system. In both processes, a time delay is assumed. A loss of EPO is included at the injection site (k_e^{F}) and in the lymphatic system (k_e^L). The structure of this

injection model is described in detail in [4, 8]. The model equations read as follows: The general EPO injection function $\mathrm{EPO}^{\mathrm{inj}}(t)$ is modelled by a sum of pulse functions

$$\mathrm{EPO}^{\mathrm{inj}}(t) = \frac{\mathrm{EPO}^{\mathrm{dose}}}{\mathrm{EPO}_{\mathrm{t\_inf}}} \cdot \sum_{i=1}^{N} \big(\mathrm{Hv}(t-\tilde{t_i}) - \mathrm{Hv}(t-\tilde{t_i}-\mathrm{EPO}_{\mathrm{t\_inf}})\big), \quad \text{where} \tag{A.17}$$

$$\mathrm{Hv}(t) = \begin{cases} 0 : t \leq 0 \\ 1 : t > 0 \end{cases} . \tag{A.18}$$

is the Heaviside-function, $\tilde{t_i}$ are time points at which EPO at dose $\mathrm{EPO}^{\mathrm{dose}}$ is administered. The injection time $\mathrm{EPO}_{\mathrm{t\_inf}}$ is set to five minutes, and $\mathrm{EPO}^{\mathrm{dose}}$ is the administered dose in IU/kg. The dynamics of the EPO concentration in the subcutaneous tissue $C^{\mathrm{SC}}_{\mathrm{EPO}}(t)$ is described by:

$$\begin{aligned} \frac{d}{dt} C^{\mathrm{SC}}_{\mathrm{EPO}}(t) &= \mathrm{EPO}^{\mathrm{inj}}(t) - C^{\mathrm{SC}}_{\mathrm{EPO}}(t) \cdot \big(k^{\mathrm{F}}_a + k_{\mathrm{FL}} + k^{\mathrm{F}}_e\big), \\ C^{\mathrm{SC}}_{\mathrm{EPO}}(0) &= 0. \end{aligned} \tag{A.19}$$

where k^{F}_a is the absorption constant for the direct influx into the central compartment, k^{F}_e is a loss term at injection site, and k_{FL} is the absorption constant of the lymphatic system. The efflux from the subcutaneous tissue into the peripheral blood is delayed by four delay compartments, i.e.

$$\frac{dC^{(i)}_{\mathrm{EPO\_F}}(t)}{dt} = C^{(i-1)}_{\mathrm{EPO\_F\_out}}(t) - k^{\mathrm{F}}_{\mathrm{Delay}} \cdot C^{(i)}_{\mathrm{EPO\_F}}(t), \quad i = 1, \ldots, 4, \tag{A.20}$$

with the settings

$$\begin{aligned} C^{(0)}_{\mathrm{EPO\_F\_out}}(t) &= C^{\mathrm{SC}}_{\mathrm{EPO}}(t) \cdot k^{\mathrm{F}}_a, \qquad \text{and} \\ C^{(i)}_{\mathrm{EPO\_F\_out}}(t) &= k^{\mathrm{F}}_{\mathrm{Delay}} \cdot C^{(i)}_{\mathrm{EPO\_F}}(t) \quad i = 1, \ldots, 4. \end{aligned}$$

$C^{(4)}_{\mathrm{EPO\_F\_out}}(t)$ enters the central EPO compartment. Analogously, the lymphatic absorption is modelled by a delay function with four compartments:

$$\begin{aligned} \frac{dC^{(i)}_{\mathrm{EPO\_L}}(t)}{dt} &= C^{(i-1)}_{\mathrm{EPO\_L\_out}}(t) - k^{\mathrm{L}}_{\mathrm{Delay}} \cdot C^{(i)}_{\mathrm{EPO\_L}}(t), \quad i = 1, \ldots, 4, \\ C^{(0)}_{\mathrm{EPO\_L\_out}}(t) &= C^{\mathrm{SC}}_{\mathrm{EPO}}(t) \cdot k_{\mathrm{FL}}, \\ C^{(i)}_{\mathrm{EPO\_L\_out}}(t) &= k^{\mathrm{L}}_{\mathrm{Delay}} \cdot C^{(i)}_{\mathrm{EPO\_L}}(t) \quad i = 1, \ldots, 4, \end{aligned} \tag{A.21}$$

where $C^{(4)}_{\mathrm{EPO\_L\_out}}(t)$ enters the central compartment. Thus, EPO dynamics in the lymphatic compartment $C^{\mathrm{L}}_{\mathrm{EPO}}$ are given by

$$\begin{aligned} \frac{d}{dt} C^{\mathrm{L}}_{\mathrm{EPO}}(t) &= C^{(4)}_{\mathrm{EPO\_L\_out}}(t) - C^{\mathrm{L}}_{\mathrm{EPO}}(t) \cdot (k^{\mathrm{L}}_a + k^{\mathrm{L}}_e), \\ C^{\mathrm{L}}_{\mathrm{EPO}}(0) &= 0, \end{aligned} \tag{A.22}$$

where k_a^{L} is the absorption constant of the transition between the lymphatic and the central compartment and k_e^{L} is a loss term. Summarising the effluxes of the direct and the lymphatic way of absorption yields

$$\mathrm{EPO}^{\mathrm{exogen}}(t) = C_{\mathrm{EPO}}^{\mathrm{L}}(t) \cdot k_a^L + C_{\mathrm{EPO\_F\_out}}^{(4)}(t), \tag{A.23}$$

for subcutaneously administered EPO and

$$\mathrm{EPO}^{\mathrm{exogen}}(t) = \mathrm{EPO}^{\mathrm{inj}}(t). \tag{A.24}$$

for intravenous injections.

Central EPO Compartment

Dynamics of Erythropoietin in central compartment (circulation) is modelled in the following way (see [1,4,9]):

$$\begin{aligned}\frac{dC_{\mathrm{EPO}}^{\mathrm{cent}}}{dt} &= P_{\mathrm{EPO}}^{\mathrm{endo}}(t) - k_{\mathrm{on}} \cdot R(t) \cdot C_{\mathrm{EPO}}^{\mathrm{cent}}(t) + k_{\mathrm{off}} \cdot C_{\mathrm{EPO}}^{\mathrm{rb}}(t) - k_{\mathrm{el}} \cdot C_{\mathrm{EPO}}^{\mathrm{cent}}(t) \\ &\quad - k_{12} \cdot C_{\mathrm{EPO}}^{\mathrm{cent}}(t) + k_{21} \cdot C_{\mathrm{EPO}}^{\mathrm{peri}}(t) + \mathrm{EPO}^{\mathrm{exogen}}(t),\end{aligned} \tag{A.25}$$

with

$$P_{\mathrm{EPO}}^{\mathrm{endo}}(t) = \left(C_{\mathrm{EPO}}^{\mathrm{cent}}(0) \cdot k_{\mathrm{el}} - C_{\mathrm{EPO}}^{\mathrm{rb}}(0) \cdot k_{\mathrm{off}} + k_{\mathrm{on}} \cdot R(0) \cdot C_{\mathrm{EPO}}^{\mathrm{cent}}(0)\right) \cdot \mathrm{EPO}_{\mathrm{prod}}, \tag{A.26}$$

where $\mathrm{EPO}_{\mathrm{prod}}$ equals one in steady state, so that equation A.25 equals zero.

The peripheral compartment and the EPO-receptor complex are described by

$$\begin{aligned}\frac{dC_{\mathrm{EPO}}^{\mathrm{peri}}}{dt} &= k_{12} \cdot C_{\mathrm{EPO}}^{\mathrm{cent}}(t) - k_{21} \cdot C_{\mathrm{EPO}}^{\mathrm{peri}}(t) \\ \frac{dC_{\mathrm{EPO}}^{\mathrm{rb}}}{dt} &= k_{\mathrm{on}} \cdot R(t) \cdot C_{\mathrm{EPO}}^{\mathrm{cent}}(t) - (k_{\mathrm{off}} + k_{\mathrm{int}}) \cdot C_{\mathrm{EPO}}^{\mathrm{rb}}(t).\end{aligned}$$

The dynamics of the EPO receptors R is determined on the basis of the bone marrow content of the cell kinetic model, namely the compartments C_{BE}, C_{CE}, C_{PEB}, C_{MEB}, and C_{RET} [4].

To account for different receptor densities of erythropoietic bone marrow cells we introduced weighting factors w_{RET}, w_{MEB}, w_{PEB}, w_{CE}, and w_{BE}. CFU-E have the highest weighting factor due to the highest number of EPO receptors observed [10]: $w_{\mathrm{BE}} \leq w_{\mathrm{CE}}$. The receptor density declines with further maturation: $w_{\mathrm{RET}} \leq w_{\mathrm{MEB}} \leq w_{\mathrm{PEB}} \leq w_{\mathrm{CE}}$. Hence

$$\frac{dR}{dt} = k_{\mathrm{off}} \cdot C_{\mathrm{EPO}}^{\mathrm{rb}}(t) - k_{\mathrm{on}} \cdot R(t) \cdot C_{\mathrm{EPO}}^{\mathrm{cent}}(t) - k_{\mathrm{deg}} \cdot R(t) + k_{\mathrm{syn}} \cdot R^{\mathrm{rel}}(t) \tag{A.27}$$

where $R^{\text{rel}}(t) = \frac{w_{\text{RET}} \cdot C_{\text{RET}} + w_{\text{MEB}} \cdot C_{\text{MEB}} + w_{\text{PEB}} \cdot C_{\text{PEB}} + w_{\text{CE}} \cdot C_{\text{CE}} + w_{\text{BE}} \cdot C_{\text{BE}}}{w_{\text{RET}} \cdot C^{\text{nor}}_{\text{RET}} + w_{\text{MEB}} \cdot C^{\text{nor}}_{\text{MEB}} + w_{\text{PEB}} \cdot C^{\text{nor}}_{\text{PEB}} + w_{\text{CE}} \cdot C^{\text{nor}}_{\text{CE}} + w_{\text{BE}} \cdot C^{\text{nor}}_{\text{BE}}}$ is the number of EPO receptors relative to steady-state [4].

The initial values are derived from steady-state conditions

$$
\begin{aligned}
C^{\text{rb}}_{\text{EPO}}(0) &= \frac{k_{\text{on}}}{k_{\text{off}} + k_{\text{int}}} \cdot R(0) \cdot C^{\text{cent}}_{\text{EPO}}(0) \\
P^{\text{endo}}_{\text{EPO}}(0) &= C^{\text{cent}}_{\text{EPO}}(0) \cdot k_{\text{el}} - C^{\text{rb}}_{\text{EPO}}(0) \cdot k_{\text{off}} + k_{\text{on}} \cdot R(0) \cdot C^{\text{cent}}_{\text{EPO}}(0) \\
C^{\text{peri}}_{\text{EPO}}(0) &= \frac{k_{12}}{k_{21}} \cdot C^{\text{cent}}_{\text{EPO}}(0)
\end{aligned}
$$

where $C^{\text{cent}}_{\text{EPO}}(0) = \text{EPO}_{\text{serum}} \cdot \text{EPO}_{\text{Vc}}$, $\text{EPO}_{\text{serum}} = 15$ IU/l is the basic level of endogenous EPO, and EPO_{Vc} denotes the distribution volume of EPO [9]. According to [9], we set $R(0) = 64.31$ and from equation A.27 it follows that $k_{\text{syn}} = k_{\text{deg}} \cdot R(0) + k_{\text{int}} \cdot C^{\text{rb}}_{\text{EPO}}(0)$. The relative internalised EPO ($C^{\text{rel}}_{\text{EPO\_int}}$) is used as argument of the regulatory functions regulated by EPO:

$$
\begin{aligned}
C_{\text{EPO\_int}}(t) &= C^{\text{rb}}_{\text{EPO}}(t) \cdot k_{\text{int}} \\
C^{\text{rel}}_{\text{EPO\_int}}(t) &= \frac{C_{\text{EPO\_int}}(t)}{C_{\text{EPO\_int}}(0)}.
\end{aligned}
$$

A.7 Granulopoiesis

Here we present model equations of our granulopoiesis model (see also [3]). Initial conditions are derived again from steady-state conditions.

Compartment CG

The model equations of CG are similar to [3]. The influence of red blood cell line on the proliferative fraction is modelled in analogy to [1].

$$
\begin{aligned}
A_{\text{CG}} &= Z_{A_{\text{CG}}}(C^{\text{cent\_rel}}_{\text{GCSF}}) \\
T_{\text{CG}} &= Z_{T_{\text{CG}}}(C^{\text{cent\_rel}}_{\text{GCSF}}) \\
\frac{d}{dt} C_{\text{CG}} &= \alpha_G C^{\text{out}}_S A^{\text{in}}_{\text{CG}} - C_{\text{CG}} \frac{a_{\text{CG}}}{T_{\text{CG}}} - k_{\text{CG}} \Psi_{\text{CG}} C_{\text{CG}} \qquad \text{(A.28)} \\
C^{\text{out}}_{\text{CG}} &= C_{\text{CG}} A^{\text{out}}_{\text{CG}} \frac{a_{\text{CG}}}{T_{\text{CG}}},
\end{aligned}
$$

where a_{CG} is the proliferative fraction and $\alpha_G = 0.8$ is the part of cells differentiating into the white blood cell line [1]. The amplification A_{CG} and the transit time T_{CG} are regulated by the concentration of G-CSF in the central compartment.

Compartment PGB

$$A_{\text{PGB}} = Z_{A_{\text{PGB}}}\left(C_{\text{GCSF}}^{\text{cent\_rel\_del}}\right) \tag{A.29}$$

$$T_{\text{PGB}} = Z_{T_{\text{PGB}}}\left(C_{\text{GCSF}}^{\text{cent\_rel\_del}}\right) \tag{A.30}$$

$$\frac{d}{dt}C_{\text{PGB}} = C_{\text{CG}}^{\text{out}}A_{\text{PGB}}^{\text{in}} - \frac{C_{\text{PGB}}}{T_{\text{PGB}}} - k_{\text{PGB}}\Psi_{\text{PGB}}C_{\text{PGB}} \tag{A.31}$$

$$C_{\text{PGB}}^{\text{out}} = A_{\text{PGB}}^{\text{out}}\frac{C_{\text{PGB}}}{T_{\text{PGB}}} \tag{A.32}$$

Compartment MGB

This compartment is divided into three compartments denoted as $G4$, $G5$ and $G6$. The compartments are again divided into N_X subcompartments to model the maturation process by a delay. In these subcompartments, the effect of postmitotic apoptosis is also implemented [2, 11] by introducing a postmitotic amplification detoned again as A. It holds that $A \leq 1$ for all subcompartments.

$$C_{\text{MGB}} = C_{G4} + C_{G5} + C_{G6} \tag{A.33}$$

$$C_{\text{MGB}}^{\text{out}} = C_{C6}^{\text{out}} \tag{A.34}$$

$$A_{G4} = Z_{A_{G4}}\left(C_{\text{GCSF}}^{\text{cent\_rel\_del}}\right) \tag{A.35}$$

$$T_{G4} = Z_{T_{G4}}\left(C_{\text{GCSF}}^{\text{cent\_rel\_del}}\right) \tag{A.36}$$

$$C_{G4} = \sum_{i=1}^{N_{G4}} C_{G4\_i} \tag{A.37}$$

$$\frac{d}{dt}C_{G4\_1} = C_{\text{PGB}}^{\text{out}} - C_{G4\_1}\frac{N_{G4}}{T_{G4}} - k_{\text{MGB}}\Psi_{\text{MGB}}C_{G4\_1} \tag{A.38}$$

$$\frac{d}{dt}C_{G4\_i} = C_{G4\_(i-1)}^{\text{out}} - C_{G4\_i}\frac{N_{G4}}{T_{G4}} - k_{\text{MGB}}\Psi_{\text{MGB}}C_{G4\_i}, \quad i = 2, \ldots, N_{G4} \tag{A.39}$$

$$C_{G4\_i}^{\text{out}} = A_{G4\_i}C_{G4\_i}\frac{N_{G4}}{T_{G4}}, \quad i = 1, \ldots, N_{G4} \tag{A.40}$$

$$C_{G4}^{\text{out}} = C_{G4\_N_{G4}}^{\text{out}} \tag{A.41}$$

The total postmitotic amplification A_{G4} is equally distributed over all subcompartments in which there is postmitotic amplification, e.g. if there is postmitotic amplification in all subcompartments, it holds that $A_{G4\_i} = {A_{G4}}^{1/N_{G4}}$.

For $G5$ and G6 the equations are completely analogous to (A.35)–(A.41) if one replaces PGB by G4 and $G5$ respectively. In the present form of our model, postmitotic amplification is restricted to G6.

Compartment GRA

$$\frac{d}{dt}C_{\mathrm{GRA}} = C^{\mathrm{out}}_{\mathrm{MGB}} - C_{\mathrm{GRA}}\frac{1}{T_{\mathrm{GRA}}} \tag{A.42}$$

$$T_{\mathrm{GRA}} = T^{\mathrm{nor}}_{\mathrm{GRA}}\left(1 + T^{\mathrm{Pred}}_{\mathrm{GRA}}\Psi_{\mathrm{Pred}}\right) \tag{A.43}$$

where Ψ_{Pred} is the characteristic function of Prednisone applications modelled as a step-function which is equal to one for the duration of one day after Prednisone application.

Granulopoietic cells

$$C_G = C_{\mathrm{CG}} + C_{\mathrm{PGB}} + C_{\mathrm{MGB}} \tag{A.44}$$

G-CSF

According to [2], the relative G-CSF production $P^{\mathrm{endo}}_{\mathrm{GCSF}}$ is a regulatory function of the relative content of segmented granulocytes in bone marrow and granulocytes in circulation.

$$P^{\mathrm{endo}}_{\mathrm{GCSF}} = Z\left(\frac{\omega_{G6}C_{G6} + \omega_{\mathrm{GRA}}C_{\mathrm{GRA}}}{\omega_{G6}C^{\mathrm{nor}}_{G6} + \omega_{\mathrm{GRA}}C^{\mathrm{nor}}_{\mathrm{GRA}}}\right), \tag{A.45}$$

where ω_{G6} and ω_{GRA} are weighting parameters and $P^{\mathrm{endo\_nor}}_{\mathrm{GCSF}} = 1$.

The G-CSF injection function reads as follows:

$$P^{\mathrm{exo}}_{\mathrm{GCSF}} = \sum_{i=1}^{L} d_{\mathrm{GCSF}}\left(t_i\right)\frac{\mathrm{Hv}\left(t-t_i\right) - \mathrm{Hv}\left(t-t_i-t_{\mathrm{inf}}\right)}{t_{\mathrm{inf}}} \tag{A.46}$$

where $\mathrm{Hv}(t)$ is the Heaviside-function (equation A.18), $t_i \geq 0$ $(i = 1, \ldots, L)$ are the time points of G-CSF injections and $d_{\mathrm{GCSF}}\left(t_i\right)$ are the corresponding doses (in μg). The injection time t_{inf} is set to 5s. The injection function is specific for each G-CSF derivative (details see [3]).

The subcutaneous compartment is divided into two subcompartments sc_1 and sc_2 where the efflux of the first compartment is the influx to the second compartment. G-CSF is applied to the first subcompartment (second term of A.47). In the first subcompartment there is a dose-dependent loss of G-CSF modelled by a Michaelis-Menten kinetic (third term of A.47). For Filgrastim injections it holds that

$$\frac{d}{dt}C^{\mathrm{sc\_1}}_{\mathrm{GCSF}} = P^{\mathrm{exo\_sc\_fil}}_{\mathrm{GCSF}} - k^{\mathrm{F}}_{sc}C^{sc\_1}_{\mathrm{GCSF}} - \frac{v^{\mathrm{F}}_{\mathrm{max}}C^{\mathrm{sc\_1}}_{\mathrm{GCSF}}}{k^{\mathrm{F}}_{m} + C^{\mathrm{sc\_1}}_{\mathrm{GCSF}}} \tag{A.47}$$

$$\frac{d}{dt}C^{\mathrm{sc\_2}}_{\mathrm{GCSF}} = k^{\mathrm{F}}_{\mathrm{sc}}\left(C^{\mathrm{sc\_1}}_{\mathrm{GCSF}} - C^{\mathrm{sc\_2}}_{\mathrm{GCSF}}\right) \tag{A.48}$$

with the initial values $C^{\mathrm{sc\_1}}_{\mathrm{GCSF}}(0) = C^{\mathrm{sc\_2}}_{\mathrm{GCSF}}(0) = 0$. For Pegfilgrastim injections the first term of the right-hand side of A.47 is substituted by $P^{\mathrm{exo\_peg\_sc}}_{\mathrm{GCSF}}$. Likewise, the Filgrastim parameters $k^{\mathrm{F}}_{\mathrm{sc}}$, $v^{\mathrm{F}}_{\mathrm{max}}$ and k^{F}_{m} are substituted by corresponding Pegfilgrastim parameters.

For Filgrastim injections it holds that

$$\begin{aligned}\frac{d}{dt}C_{\mathrm{GCSF}}^{\mathrm{cent}} &= P_{\mathrm{GCSF}}^{\mathrm{ref}}P_{\mathrm{GCSF}}^{\mathrm{endo}} + P_{\mathrm{GCSF}}^{\mathrm{exo\_fil\_iv}} + k_{\mathrm{sc}}^{\mathrm{F}}C_{\mathrm{GCSF}}^{\mathrm{sc\_2}} \\ &-k_u^{\mathrm{F}}C_{\mathrm{GCSF}}^{\mathrm{cent}} - k_{\mathrm{cp}}^{\mathrm{F}}C_{\mathrm{GCSF}}^{\mathrm{cent}} + k_{\mathrm{pc}}^{\mathrm{F}}C_{\mathrm{GCSF}}^{\mathrm{per}} - \frac{v_{\mathrm{max}}^{\mathrm{GRA\_F}}C_{\mathrm{GCSF}}^{\mathrm{cent}}}{k_m^{\mathrm{GRA\_F}} + C_{\mathrm{GCSF}}^{\mathrm{cent}}}C_{\mathrm{GRA}}^{\mathrm{rel}}.\end{aligned} \tag{A.49}$$

The balance equation A.49 contains the following terms: $P_{\mathrm{GCSF}}^{\mathrm{ref}}P_{\mathrm{GCSF}}^{\mathrm{endo}}$ is the endogenous production, $P_{\mathrm{GCSF}}^{\mathrm{exo\_fil\_iv}}$ is the intravenous injection, $k_{\mathrm{sc}}^{\mathrm{F}}C_{\mathrm{GCSF}}^{\mathrm{sc\_2}}$ is the influx from the subcutaneous compartment, $k_u^{\mathrm{F}}C_{\mathrm{GCSF}}^{\mathrm{cent}}$ is the unspecific elimination, $k_{\mathrm{cp}}^{\mathrm{F}}C_{\mathrm{GCSF}}^{\mathrm{cent}}$ is the efflux to the peripheral compartment, $k_{\mathrm{pc}}^{\mathrm{F}}C_{\mathrm{GCSF}}^{\mathrm{per}}$ is the influx from the peripheral compartment and $\frac{v_{\mathrm{max}}^{\mathrm{GRA\_F}}C_{\mathrm{GCSF}}^{\mathrm{cent}}}{k_m^{\mathrm{GRA\_F}}+C_{\mathrm{GCSF}}^{\mathrm{cent}}}C_{\mathrm{GRA}}^{\mathrm{rel}}$ is the specific elimination. The corresponding equation for Pegfilgrastim is the same except for the endogenous production, which is zero, the intravenous injection function which is substituted by $P_{\mathrm{GCSF}}^{\mathrm{exo\_peg\_iv}}$, the parameters and the initial value which is again zero.

With $C_{\mathrm{GCSF}}^{\mathrm{cent}}(0) = C_{\mathrm{GCSF}}^{\mathrm{cent\_nor}} = V_D^{\mathrm{F}}C_{\mathrm{GCSF}}^{\mathrm{cent\_ref}}$, the parameter $P_{\mathrm{GCSF}}^{\mathrm{ref}}$ can be calculated from the steady-state conditions $P_{\mathrm{GCSF}}^{\mathrm{endo}}(0) = 1$, $P_{\mathrm{GCSF}}^{\mathrm{exo\_fil\_iv}}(0) = 0$ and $-k_{\mathrm{cp}}^{\mathrm{F}}C_{\mathrm{GCSF}}^{\mathrm{cent}}(0) + k_{\mathrm{pc}}^{\mathrm{F}}C_{\mathrm{GCSF}}^{\mathrm{per}}(0) = 0$:

$$P_{\mathrm{GCSF}}^{\mathrm{ref}} = V_D^{\mathrm{F}}C_{\mathrm{GCSF}}^{\mathrm{cent\_ref}}\left(k_u^{\mathrm{F}} + \frac{v_{\mathrm{max}}^{\mathrm{GRA\_F}}}{k_m^{\mathrm{GRA\_F}} + V_D^{\mathrm{F}}C_{\mathrm{GCSF}}^{\mathrm{cent\_ref}}}\right), \tag{A.50}$$

where V_D^{F} is the distribution volume and $C_{\mathrm{GCSF}}^{\mathrm{cent\_ref}}$ is the reference G-CSF serum concentration.

For both G-CSF derivatives, we have transitions between central and peripheral compartment:

$$\frac{d}{dt}C_{\mathrm{GCSF}}^{\mathrm{per}} = k_{\mathrm{cp}}C_{\mathrm{GCSF}}^{\mathrm{cent}} - k_{\mathrm{pc}}C_{\mathrm{GCSF}}^{\mathrm{per}} \tag{A.51}$$

$$C_{\mathrm{GCSF}}^{\mathrm{per}}(0) = C_{\mathrm{GCSF}}^{\mathrm{per\_nor}} = V_D\frac{k_{\mathrm{cp}}}{k_{\mathrm{pc}}}C_{\mathrm{GCSF}}^{\mathrm{cent\_ref}} \tag{A.52}$$

where the parameters k_{cp}, k_{pc} and V_D are specific for Filgrastim and Pegfilgrastim respectively. To model the competition of Pegfilgrastim and endogenous G-CSF with respect to receptor binding, the regulatory functions of Pegfilgrastim and Filgrastim were again combined using the weighting factor ω_P defined in equation A.16:

$$Z_Y = \omega_P \cdot Z_Y\left(C_{\mathrm{GCSF}}^{\mathrm{cent\_rel\_del\_peg}}\right) + (1-\omega_P)\cdot Z_Y\left(C_{\mathrm{GCSF}}^{\mathrm{cent\_rel\_del\_fil}}\right)$$

where Y is an arbitrary quantity regulated by G-CSF such as transition times or amplifications. For all these quantities we assumed the same regulatory function of the weighting parameter ω_P.

A.8 Parameters

Here, we present all parameters of the model, their values, and how they were determined. In table A.3 we present general parameters of the cell kinetic model of granulopoiesis.

Table A.3: G-CSF PK/PD parameters

parameter	meaning	value	
α_G	percentage of stem cells differentiating to myeloid cells	0.8	
α_E	percentage of stem cells differentiating to erythroid cells	0.15	
S^{nor}	normal value of stem cells	1	set
τ_S	duration of cell cycle	8	[1], p. 70
p_δ	self-renewal probability	0.1	[1], p. 70
a_S^{min}	proliferative fraction under minimal stimulation	0.01	[1], p. 70
a_S^{nor}	proliferative fraction under normal stimulation	0.15	[1], p. 70
a_S^{int}	proliferative fraction under intensified stimulation	0.45	[1], p. 70
a_S^{max}	proliferative fraction under maximal stimulation	1	[1], p. 70
w_E	weighting parameter E for regulation of a	0.3	[1], p. 70
w_G	weighting parameter G for regulation of a	0.1	[1], p. 70
w_S	weighting parameter S for regulation of a	1	[1], p. 70
ϑ_E	weighting parameter E for regulation of p	-2	[1], p. 70
ϑ_G	weighting parameter G for regulation of p	-8	[1], p. 70
N_{G4}	number of subcompartments in $G4$	5	set
N_{G5}	number of subcompartments in $G5$	5	set
N_{G6}	number of subcompartments in $G6$	5	set
$a_{\mathrm{CG}}^{\mathrm{min}}$	proliferative fraction under minimal stimulation	0.1205	fitted
$a_{\mathrm{CG}}^{\mathrm{nor}}$	proliferative fraction under normal stimulation	0.1252	fitted
$a_{\mathrm{CG}}^{\mathrm{max}}$	proliferative fraction under intensified stimulation	1	[1], p. 71
$a_{\mathrm{CG}}^{\mathrm{int}}$	proliferative fraction under maximal stimulation	0.8340	fitted
w_{GRA}	influence of GRA on G-CSF production	1	set [12]
w_{G6}	influence of $G6$ on G-CSF production	0.2	set [12]
$P_{\mathrm{GCSF}}^{\mathrm{endo\_max}}$	maximal G-CSF production	257.5	fitted
$P_{\mathrm{GCSF}}^{\mathrm{endo\_nor}}$	normal G-CSF production	1	set
$P_{\mathrm{GCSF}}^{\mathrm{endo\_min}}$	minimal G-CSF production	0.3179	fitted
$P_{\mathrm{GCSF}}^{\mathrm{endo\_b}}$	sensitivity parameter of G-CSF production	0.0220	fitted
$T_{\mathrm{GRA}}^{\mathrm{nor}}$	transition time of granulocytes	5.576	fitted [13]
$T_{\mathrm{GRA}}^{\mathrm{pred}}$	prolongation of T_{GRA} under Prednisone	0.4659	fitted

In table A.4 we present parameters of the granulopoiesis model which are assumed to be different for Filgrastim and Pegfilgrastim respectively. Note that for endogenous G-CSF, we assumed the same parameter setting as for Filgrastim.

Table A.4: G-CSF pharmacokinetic and -dynamic parameters

parameter	meaning	value	
Filgrastim			
$k_{\mathrm{sc}}^{\mathrm{F}}$	subcutaneous absorption	0.1613	fitted
k_m^{F}	Michaelis-Menten constant of subcutaneous elimination	34.68	fitted
$v_{\mathrm{max}}^{\mathrm{F}}$	Maximum of subcutaneous elimination	67.27	fitted
k_u^{F}	unspecific elimination	0.4408	fitted
k_{12}^{F}	transition central to peripheral	0.0001	fitted
k_{21}^{F}	transition peripheral to central	0.3564	indeterminable
VD_F	distribution volume	1.156	fitted
$v_{\mathrm{max}}^{\mathrm{GRA}_F}$	Maximum of specific elimination	4.769	fitted

$k_m^{\mathrm{GRA}_F}$	Michaelis-Menten constant of specific elimination	22.38	fitted
$T_{\mathrm{CG}_F}^{\min}$	transition time in CG under minimal stimulation	47.26	fitted
$T_{\mathrm{CG}_F}^{\mathrm{nor}}$	transition time in CG under normal stimulation	78.19	fitted
$T_{\mathrm{CG}_F}^{\max}$	transition time in CG under maximal stimulation	286.3	fitted
$T_{\mathrm{CG}_F}^{\mathrm{b}}$	sensitivity of transition time in CG	0.5901	fitted
$A_{\mathrm{CG}_F}^{\min}$	amplification in CG under minimal stimulation	0.9101	fitted
$A_{\mathrm{CG}_F}^{\mathrm{nor}}$	amplification in CG under normal stimulation	104.6	fitted
$A_{\mathrm{CG}_F}^{\max}$	amplification in CG under maximal stimulation	206.2	fitted
$A_{\mathrm{CG}_F}^{\mathrm{b}}$	sensitivity of amplification in CG	0.0241	fitted
$A_{\mathrm{PGB}_F}^{\min}$	amplification in PGB under minimal stimulation	1.307	fitted
$A_{\mathrm{PGB}_F}^{\mathrm{nor}}$	amplification in PGB under normal stimulation	61.20	fitted
$A_{\mathrm{PGB}_F}^{\max}$	amplification in PGB under maximal stimulation	814.7	fitted
$A_{\mathrm{PGB}_F}^{\mathrm{b}}$	sensitivity of amplification in PGB	0.7212	fitted
$T_{\mathrm{PGB}_F}^{\min}$	transition time in PGB under minimal stimulation	4.640	fitted
$T_{\mathrm{PGB}_F}^{\mathrm{nor}}$	transition time in PGB under normal stimulation	40.89	fitted
$T_{\mathrm{PGB}_F}^{\max}$	transition time in PGB under maximal stimulation	217.1	fitted
$T_{\mathrm{PGB}_F}^{\mathrm{b}}$	sensitivity of transition time in PGB	0.1041	fitted
$A_{G4_F}^{\mathrm{nor}}$	postmitotic amplification in $G4$	1	set
$T_{G4_F}^{\min}$	transition time in $G4$ under minimal stimulation	119.2	fitted
$T_{G4_F}^{\mathrm{nor}}$	transition time in $G4$ under normal stimulation	11.37	fitted
$T_{G4_F}^{\max}$	transition time in $G4$ under maximal stimulation	3.93	fitted
$T_{G4_F}^{\mathrm{b}}$	sensitivity of transition time in $G4$	0.3661	fitted
$A_{G5_F}^{\mathrm{nor}}$	postmitotic amplification in $G5$	1	set
$T_{G5_F}^{\min}$	transition time in $G5$ under minimal stimulation	48.3	fitted
$T_{G5_F}^{\mathrm{nor}}$	transition time in $G5$ under normal stimulation	37.00	fitted
$T_{G5_F}^{\max}$	transition time in $G5$ under maximal stimulation	4.64	fitted
$T_{G5_F}^{\mathrm{b}}$	sensitivity of transition time in $G5$	0.4588	fitted
$A_{G6_F}^{\min}$	postmitotic amplification in $G6$ under minimal stimulation	0.2005	fitted
$A_{G6_F}^{\mathrm{nor}}$	postmitotic amplification in $G6$ under normal stimulation	0.2488	fitted
$A_{G6_F}^{\max}$	postmitotic amplification in $G6$ under maximal stimulation	0.8495	fitted
$A_{G6_F}^{\mathrm{b}}$	sensitivity of postmitotic amplification in $G6$	0.5034	fitted
$T_{G6_F}^{\min}$	transition time in $G6$ under minimal stimulation	140.9	fitted
$T_{G6_F}^{\mathrm{nor}}$	transition time in $G6$ under normal stimulation	82.02	fitted
$T_{G6_F}^{\max}$	transition time in $G6$ under maximal stimulation	41.38	fitted
$T_{G6_F}^{\mathrm{b}}$	sensitivity of transition time in $G6$	0.5260	fitted
Pegfilgrastim			
k_{sc}^P	subcutaneous absorption	0.1071	fitted
k_m^P	Michaelis-Menten constant of subcutaneous elimination	5.456	fitted
$v_{\max}^{\mathrm{P}}$	Maximum of subcutaneous elimination	16.50	fitted
k_u^P	unspecific elimination	0.0874	fitted
$v_{\max}^{\mathrm{GRA}_P}$	Maximum of specific elimination	5.159	fitted
$k_m^{\mathrm{GRA}_P}$	Michaelis-Menten constant of specific elimination	30.82	fitted
VD_P	distribution volume	4.091	fitted
k_{12}^P	transition central to peripheral	0.0746	fitted
k_{21}^P	transition peripheral to central	0.5475	fitted
$\omega_P^{\min}$	minimum of weighting function	0	set
ω_P^{nor}	value of weighting function for $1\mu g$ Pegfilgrastim	0.4994	fitted
$\omega_P^{\max}$	maximum of weighting function	1	set
ω_P^{b}	sensitivity parameter of weighting function	0.0680	fitted
$A_{\mathrm{CG}_P}^{\min}$	transition time in CG under minimal stimulation	4.210	fitted

$A_{\mathrm{CG}_P}^{\mathrm{nor}}$	transition time in CG under normal stimulation	356.7	fitted
$A_{\mathrm{CG}_P}^{\mathrm{max}}$	transition time in CG under maximal stimulation	668.7	fitted
$A_{\mathrm{CG}_P}^{\mathrm{b}}$	sensitivity of transition time in CG	0.1026	fitted
$T_{\mathrm{CG}_P}^{\mathrm{min}}$	amplification in CG under minimal stimulation	0.8480	fitted
$T_{\mathrm{CG}_P}^{\mathrm{nor}}$	amplification in CG under normal stimulation	1.687	fitted
$T_{\mathrm{CG}_P}^{\mathrm{max}}$	amplification in CG under maximal stimulation	57.60	fitted
$T_{\mathrm{CG}_P}^{\mathrm{b}}$	sensitivity of amplification in CG	0.0396	fitted
$A_{\mathrm{PGB}_P}^{\mathrm{min}}$	amplification in PGB under minimal stimulation	0.0669	fitted
$A_{\mathrm{PGB}_P}^{\mathrm{nor}}$	amplification in PGB under normal stimulation	24.54	fitted
$A_{\mathrm{PGB}_P}^{\mathrm{max}}$	amplification in PGB under maximal stimulation	47.00	fitted
$A_{\mathrm{PGB}_P}^{\mathrm{b}}$	sensitivity of amplification in PGB	0.3266	fitted
$T_{\mathrm{PGB}_P}^{\mathrm{min}}$	transition time in PGB under minimal stimulation	0.7558	fitted
$T_{\mathrm{PGB}_P}^{\mathrm{nor}}$	transition time in PGB under normal stimulation	24.66	fitted
$T_{\mathrm{PGB}_P}^{\mathrm{max}}$	transition time in PGB under maximal stimulation	24.69	fitted
$T_{\mathrm{PGB}_P}^{\mathrm{b}}$	sensitivity of transition time in PGB	0.1180	fitted
$A_{G4_P}^{\mathrm{nor}}$	postmitotic amplification in $G4$	1	set
$T_{G4_P}^{\mathrm{min}}$	transition time in $G4$ under minimal stimulation	152.8	fitted
$T_{G4_P}^{\mathrm{nor}}$	transition time in $G4$ under normal stimulation	14.19	fitted
$T_{G4_P}^{\mathrm{max}}$	transition time in $G4$ under maximal stimulation	4.168	fitted
$T_{G4_P}^{\mathrm{b}}$	sensitivity of transition time in $G4$	0.2246	fitted
$A_{G5_P}^{\mathrm{nor}}$	postmitotic amplification in $G5$	1	set
$T_{G5_P}^{\mathrm{min}}$	transition time in $G5$ under minimal stimulation	152.8	fitted
$T_{G5_P}^{\mathrm{nor}}$	transition time in $G5$ under normal stimulation	14.19	fitted
$T_{G5_P}^{\mathrm{max}}$	transition time in $G5$ under maximal stimulation	4.168	fitted
$T_{G5_P}^{\mathrm{b}}$	sensitivity of transition time in $G5$	0.2246	fitted
$A_{G6_P}^{\mathrm{min}}$	postmitotic amplification in $G6$ under minimal stimulation	0.1387	fitted
$A_{G6_P}^{\mathrm{nor}}$	postmitotic amplification in $G6$ under normal stimulation	0.3677	fitted
$A_{G6_P}^{\mathrm{max}}$	postmitotic amplification in $G6$ under maximal stimulation	1	set
$A_{G6_P}^{\mathrm{b}}$	sensitivity of postmitotic amplification in $G6$	0.3213	fitted
$T_{G6_P}^{\mathrm{min}}$	transition time in $G6$ under minimal stimulation	152.8	fitted
$T_{G6_P}^{\mathrm{nor}}$	transition time in $G6$ under normal stimulation	14.19	fitted
$T_{G6_P}^{\mathrm{max}}$	transition time in $G6$ under maximal stimulation	4.168	fitted
$T_{G6_P}^{\mathrm{b}}$	sensitivity of transition time in $G6$	0.2246	fitted

Next we present parameters of the cell kinetic model of erythropoiesis not presented in table A.3 and which do not depend on the kind of EPO derivative or their mode of application (table A.5).

A number of parameters depending on EPO derivative but not on mode of application is presented in table A.6.

Table A.5: Cell kinetic parameters of the erythropoiesis model

parameter	meaning	value		
q_{RET}	proportion of RET/RBC in steady state	0.016		set
$T_{\mathrm{ERY\_rnd}}$	transition time in compartment "RANDOM"	1020.4	[14]	set
$T_{\mathrm{ERY\_age}}$	transition time in compartment "AGE"	3061.2	[14]	set
$s_{\mathrm{ERY}}^{\mathrm{nor}}$	influx regulation factor of RANDOM and AGE	0.9	[6], p. 40	set
N_{ERY}	number uf subcompartments	10	[6], p. 41	set
$\mathrm{HK}^{\mathrm{nor}}$	normal value of HK	0.43		set
$\mathrm{ERY}^{\mathrm{nor}}$	normal value of ERY	4.5		set
$\mathrm{RET}^{\mathrm{nor}}$	normal value of RET	100		set
$\mathrm{RET\%}^{\mathrm{nor}}$	normal value of RET%	9.5		set
$\mathrm{HB}^{\mathrm{nor}}$	normal value of HB	13.5		set
HB_per_ERY		0.000031		set
P_{max}^{endo}	maximal endogenous production	200	[6]	set
$\mathrm{EPO}_{\mathrm{Vc}}$	volume of distribution	0.032	[9]	set
$\mathrm{EPO}_{\mathrm{serum}}$	serum concentration	15	[9]	set
VB	blood volume	5.5	[15]	set
a_{BE}^{min}	proliferative fraction under minimal stimulation	0.3	[1], p. 71	set
a_{BE}^{nor}	proliferative fraction under normal stimulation	0.33	[1], p. 71	set
a_{BE}^{int}	proliferative fraction under maximal stimulation	0.66	[1], p. 71	set
a_{BE}^{max}	proliferative fraction under intensified stimulation	1	[1], p. 71	set

Table A.6: EPO pharmacokinetic and -dynamic parameters

parameter	meaning	Alfa, Beta, Delta, endog. EPO			Darbepoetin Alfa	
k_{el}	unspecific elimination from central compartment	0.102	fitted		0.062	fitted
k_{12}	transition central to peripheral	0.079	fitted		0.294	fitted
k_{21}	transition peripheral to central	0.084	fitted		0.291	fitted
k_{on}	receptor binding rate	0.070	fitted		0.043	fitted
k_{off}	receptor dissociation rate	14.27	fitted		9.62	fitted
$R(0)$	EPO receptors (normal value)	64.31	set	[9]	64.31	set
k_{int}	internalisation rate	2	set	[9]	1.14	fitted
k_{deg}	receptor degradation rate	0.101	fitted		0.116	fitted
w_{RET}	weighting factor for receptor density (RET)	0.05	set		0.05	set
w_{MEB}	weighting factor for receptor density (MEB)	0.087	fitted		0.125	fitted
w_{PEB}	weighting factor for receptor density (PEB)	0.293	fitted		0.509	fitted
w_{CE}	weighting factor for receptor density (CE)	3.84	fitted		2.69	fitted
w_{BE}	weighting factor for receptor density (BE)	0.0881	fitted		0.111	fitted
$T^{\mathrm{min}}_{\mathrm{BE}}$	transition time in BE under minimal stimulation	155.7	fitted		124.6	fitted
$T^{\mathrm{nor}}_{\mathrm{BE}}$	transition time in BE under normal stimulation	40	set	[14]	40	set
$T^{\mathrm{max}}_{\mathrm{BE}}$	transition time in BE under maximal stimulation	28.60	fitted		34.22	fitted
$T^{\mathrm{b}}_{\mathrm{BE}}$	sensitivity of transition time in BE	1.134	fitted		3.559	fitted
$A^{\mathrm{min}}_{\mathrm{BE}}$	amplification in BE under minimal stimulation	25.04	fitted		47.10	fitted
$A^{\mathrm{nor}}_{\mathrm{BE}}$	amplification in BE under normal stimulation	64	set	[14]	64	set
$A^{\mathrm{max}}_{\mathrm{BE}}$	amplification in BE under maximal stimulation	194.7	fitted		115.5	fitted
$A^{\mathrm{b}}_{\mathrm{BE}}$	sensitivity of amplification in BE	2.321	fitted		0.1659	fitted
$A^{\mathrm{min}}_{\mathrm{CE}}$	amplification in CE under minimal stimulation	0.9645	fitted		0.5717	fitted
$A^{\mathrm{nor}}_{\mathrm{CE}}$	amplification in CE under normal stimulation	32	set	[14]	32	set
$A^{\mathrm{max}}_{\mathrm{CE}}$	amplification in CE under maximal stimulation	104.7	fitted		127.8	fitted
$A^{\mathrm{b}}_{\mathrm{CE}}$	sensitivity of amplification in CE	0.0438	fitted		0.3956	fitted
$T^{\mathrm{min}}_{\mathrm{CE}}$	transition time in CE under minimal stimulation	186.7	fitted		387.3	fitted
$T^{\mathrm{nor}}_{\mathrm{CE}}$	transition time in CE under normal stimulation	40	set	[14]	40	set
$T^{\mathrm{max}}_{\mathrm{CE}}$	transition time in CE under maximal stimulation	15.25	fitted		36.23	fitted
$T^{\mathrm{b}}_{\mathrm{CE}}$	sensitivity of transition time in CE	0.3920	fitted		0.1305	fitted
$T^{\mathrm{min}}_{\mathrm{PEB}}$	transition time in BE under minimal stimulation	99.25	fitted		178.4	fitted
$T^{\mathrm{nor}}_{\mathrm{PEB}}$	transition time in PEB under normal stimulation	48	set		48	set
$T^{\mathrm{max}}_{\mathrm{PEB}}$	transition time in PEB under maximal stimulation	8.809	fitted		15.74	fitted
$T^{\mathrm{b}}_{\mathrm{PEB}}$	sensitivity of transition time in PEB	1.301	fitted		1.847	fitted
$A^{\mathrm{min}}_{\mathrm{PEB}}$	amplification in PEB under minimal stimulation	0.6862	fitted		0.8078	fitted
$A^{\mathrm{nor}}_{\mathrm{PEB}}$	amplification in PEB under normal stimulation	64	set	[14]	64	set
$A^{\mathrm{max}}_{\mathrm{PEB}}$	amplification in PEB under maximal stimulation	75.38	fitted		139.6	fitted
$A^{\mathrm{b}}_{\mathrm{PEB}}$	sensitivity of amplification in PEB	0.4135	fitted		0.1331	fitted
$T^{\mathrm{min}}_{\mathrm{MEB}}$	transition time in MEB under minimal stimulation	144.8	fitted		186.7	fitted
$T^{\mathrm{nor}}_{\mathrm{MEB}}$	transition time in MEB under normal stimulation	100.2	fitted		100.2	fitted
$T^{\mathrm{max}}_{\mathrm{MEB}}$	transition time in MEB under maximal stimulation	90.17	fitted		26.98	fitted
$T^{\mathrm{b}}_{\mathrm{MEB}}$	sensitivity of transition time in MEB	0.5395	fitted		0.1965	fitted

Finally, we present parameters which depend on the mode of application, i.e. parameters refering to absorption kinetics (table A.7).

Chemotherapy parameters are specific for cell stage, drug, drug doses, and sometimes, risk group of patients such as young or elderly patients. An overview of estimated parameters can be found in tables A.8 (toxicity to stem cells and granulopoiesis) and A.9 (toxicity to erythropoiesis and lymphopoiesis).

Table A.7: Parameters of EPO absorption kinetics in dependance on mode of application

		Alfa, abdomen, upper arm	Alfa, fore-arm	Alfa, shoulder	Alfa, thigh	Beta, abdomen	Beta, thigh	Beta, fore-arm	Delta	Darb-epoetin Alfa
k_a^F	transition from subcut. comp. to central comp.	0.5320	0.7786	0.7119	0.7374	0.3747	0.4528	0.4077	0.6657	3.0148
k_e^F	elimination from subcutaneous compartment	0.2713	0.1337	0.1263	0.3295	0.1062	0.0656	0.1873	0.1517	0.1938
k_{Delay}^L	delay in lymphatic system	0.0390	0.0510	0.1460	0.0298	0.0699	0.0410	0.0476	0.2367	0.0241
k_a^L	transition from lymph. comp. to central comp.	0.1172	0.0901	0.1326	0.1588	0.2309	0.2024	0.4687	0.1065	1.1192
k_e^L	elimination from lymphatic compartment	0.4334	0.4904	0.4066	0.4343	0.0950	0.0557	0.1587	0.3321	0.1769
k_{Delay}^F	delay in subcutaneous compartment	0.3275	0.3259	0.6029	0.1626	0.3705	0.2078	0.2819	0.7508	0.1161
k_{FL}	transition from subcut. comp. to lymph. comp.	1.0074	3.6252	3.9596	0.7213	1.2138	1.0453	1.1987	3.4461	5.5404

Table A.8: Chemotherapy parameters 1: We present toxicity parameters regarding stem cells and granulopoiesis lineage. Values in brackets could not be determined with sufficient accuracy due to lack of data and are irrelevant for the scenarios considered. Numbers after drug correspond to dose in mg/m^2.

		FC	Delay	S	CG	PGB	MGB
Cyclophos phamid 750 Doxorubicin 50	CHOP, age≥60	1.038	0.061	0.023	0.915	0.334	0.040
Etoposid 100	CHOEP, age≥60	8.425	0.116	0.000	0.008	0.500	0.002
Cyclophos phamid 750 Doxorubicin 50	CHOP, age<60	1.038	0.061	0.135	0.653	0.013	0.001
Etoposid 100	CHOEP, age<60	8.425	0.116	0.000	0.008	0.071	0.002
Procarbazine 100	BEACOPP, BEACOPP escalated	1.042	0.019	0.000	0.010	0.010	0.057
Cyclophos phamid 650 Doxorubicin 25	BEACOPP, BEACOPP escalated	1.038	0.061	0.087	0.591	0.013	0.000
Bleomycin 10	BEACOPP, BEACOPP escalated	1.355	0.002	0.002	0.064	0.028	0.000
Cyclophos phamid 1250, Doxorubicin 35	BEACOPP escalated	1.038	0.061	0.135	7.273	0.043	0.001
Etoposid 200	BEACOPP escalated	8.425	0.116	0.000	0.008	0.051	0.001
Cyclophos phamid 1400 Doxorubicin 32.5	high CHOEP	1.038	0.061	0.135	7.708	0.376	0.001
Etoposid 175	high CHOEP	8.425	0.116	0.000	0.009	0.478	0.002
Carboplatin, Paclitaxel 225		1.000	0.077	0.001	60.00	60.00	0.000
Doxorubicin 60 Docetaxel 75		2.014	0.072	0.014	0.228	4.215	0.000
Paclitaxel 225	ETC	1.277	0.018	0.301	0.891	3.495	0.001
Paclitaxel 175	EC-T	1.277	0.018	0.000	0.891	3.495	0.001
Cyclophos phamid 600	EC-T	1.121	0.064	0.136	0.733	0.093	0.000
Cyclophos phamid 2500	ETC	1.121	0.064	0.136	0.742	0.130	0.000
Epirubicin 90	EC-T	1.308	0.050	0.000	0.054	0.189	0.001
Epirubicin 150	ETC	1.308	0.050	0.003	1.277	13.297	0.008
cytarabine 2	ESHAP	1.000	0.090	0.016	0.007	0.005	0.003
cisplatin 25	ESHAP	1.000	0.071	0.060	0.119	0.044	0.001
etoposide 40	ESHAP	3.341	0.116	0.000	0.037	0.012	0.000

Table A.9: Chemotherapy parameters 2: We present toxicity parameters regarding erythropoiesis and lymphopoiesis. Values in brackets could not be determined with sufficient accuracy due to lack of data and are irrelevant for the scenarios considered. Numbers after drug correspond to dose in mg/m^2.

		Delay LY	LY	BE	CE	PEB	MEB	RET
Cyclophos phamid 750 Doxorubicin 50	CHOP, age≥60	0.008	14.49	0.001	0.011	0.019	0.319	0.000
Etoposid 100	CHOEP, age≥60	0.106	0.042	0.000	0.000	0.034	0.028	0.000
Cyclophos phamid 750 Doxorubicin 50	CHOP, age<60	0.008	17.68	0.001	0.011	0.016	0.319	0.000
Etoposid 100	CHOP, age<60	0.106	0.042	0.000	0.000	0.034	0.028	0.000
Procarbazine 100	BEACOPP, BEACOPP escalated	0.000	0.000	0.000	0.000	0.000	0.000	0.000
Cyclophos phamid 650 Doxorubicin 25	BEACOPP, BEACOPP escalated	0.008	20.00	0.000	0.001	0.000	0.003	0.000
Bleomycin 10	BEACOPP, BEACOPP escalated	0.000	0.000	0.005	0.044	0.000	0.092	0.000
Cyclophos phamid 1250, Doxorubicin 35	BEACOPP escalated	0.008	40.00	0.001	0.012	0.019	0.363	0.000
Etoposid 200	BEACOPP escalated	0.106	3.602	0.000	0.000	0.009	0.019	0.000
Cyclophos phamid 1400 Doxorubicin 32.5	high CHOEP	0.008	35.85	0.003	0.016	0.024	0.407	0.000
Etoposid 175	high CHOEP	0.106	50.00	0.003	0.002	0.034	0.050	0.002
Carboplatin, Paclitaxel 225		(0.000)	(0.000)	(1.615)	(1.292)	(4.103)	(0.235)	(0.000)
Doxorubicin 60 Docetaxel 75		(0.000)	(0.000)	(0.377)	(0.377)	(0.599)	(0.000)	(0.000)
Paclitaxel 225	ETC	0.140	0.350	0.001	1.797	0.984	0.153	0.001
Paclitaxel 175	EC-T	0.140	0.334	0.000	1.423	0.046	0.010	0.000
Cyclophos phamid 600	EC-T	0.028	4.747	0.001	0.003	0.035	0.018	0.001
Cyclophos phamid 2500	ETC	0.028	12.59	0.017	0.010	0.035	0.749	0.007
Epirubicin 90	EC-T	0.030	2.697	0.000	0.023	0.170	0.023	0.001
Epirubicin 150	ETC	0.030	42.26	1.549	9.670	1.595	0.249	0.085
cytarabine 2	ESHAP	1.000	1.000	0.000	0.000	0.034	0.028	0.000
cisplatin 25	ESHAP	0.000	0.000	0.197	0.018	0.457	1.691	0.000
etoposide 40	ESHAP	0.048	1.275	0.000	0.000	0.034	0.028	0.000

A summary of data used to identify model parameters and to validate our model can be found in table A.10.

Table A.10: **Available data sets.** Studies with access to raw data are indicated with an asterisk.

	EPO	G-CSF	chemotherapy	disease	
1	Alfa 300 IU/kg, sc (tigh)	none	none	none	[16]
2	Alfa 450 IU/kg, sc (tigh)	none	none	none	[16]
3	Alfa 600 IU/kg, sc (tigh)	none	none	none	[16]
4	Alfa 900 IU/kg, sc (tigh)	none	none	none	[16]
5	Alfa 1200 IU/kg, sc (tigh)	none	none	none	[16]
6	Alfa 1350 IU/kg, sc (tigh)	none	none	none	[16]
7	Alfa 1800 IU/kg, sc (tigh)	none	none	none	[16]
8	Alfa 2400 IU/kg, sc (tigh)	none	none	none	[16]
9	Alfa 300 IU/kg, sc	none	none	none	[17]
10	Alfa 400 IU/kg, sc	none	none	none	[17]
11	Alfa 600 IU/kg, sc	none	none	none	[17]
12	Alfa 750 IU, sc (upper arm)	none	none	none	[18]
13	Alfa 6000 IU, sc (upper arm)	none	none	none	[18]
14	Alfa 150 IU/kg, sc	none	none	none	[19]
15	Alfa 300 IU/kg, sc	none	none	none	[19]
16	Alfa 100 IU/kg, iv	none	none	none	[20]
17	Alfa 200 IU/kg, iv	none	none	none	[20]
18	Alfa 100 IU/kg, sc (shoulder)	none	none	none	[20]
19	Alfa 200 IU/kg, sc (shoulder)	none	none	none	[20]
20	Alfa 50 IU/kg, sc (upper arm)	none	none	none	[21]
21	Alfa 100 IU/kg, sc (abdomen)	none	none	none	[22]
22	Alfa 40000 IU, sc (forearm)	none	none	none	[15]
23	Alfa 150 IU/kg, sc (forearm)	none	none	none	[15]
24	beta 10 IU/kg, iv	none	none	none	[23]
25	beta 50 IU/kg, iv	none	none	none	[23]
26	beta 150 IU/kg, iv	none	none	none	[23]
27	beta 500 IU/kg, iv	none	none	none	[23]
28	beta 1000 IU/kg, iv	none	none	none	[23, 24]
29	beta 5000 IU	none	none	none	[25]
30	beta 1500 IU, sc	none	none	none	[26]
31	beta 3000 IU, sc (forearm)	none	none	none	[26]
32	beta 100 IU/kg, iv	none	none	none	[27]
33	beta 100 IU/kg, sc (tigh)	none	none	none	[27]
34	beta 100 IU/kg, sc (abdomen)	none	none	none	[27]
36	delta 15 IU/kg, iv	none	none	none	[28]
37	delta 40 IU/kg, iv	none	none	none	[28]
38	delta 75 IU/kg, iv	none	none	none	[28]
39	delta 75 IU/kg, sc	none	none	none	[28]
40	delta 100 IU/kg, iv	none	none	none	[28]
41	Darbepoetin alfa 120 IU/kg, sc	none	none	none	[29]
42	Darbepoetin alfa 140 IU/kg, sc	none	none	none	[29]
43	Darbepoetin alfa 240 IU/kg, sc	none	none	none	[29]
44	Darbepoetin alfa 360 IU/kg, sc	none	none	none	[29]
45	Darbepoetin alfa 780 IU/kg, sc	none	none	none	[29]

46	Darbepoetin alfa 875 IU/kg, sc	none	none	none	[29]
47	Alfa	Fil., 480 μg, d 3-10	E-T-C*	breast cancer	[30]
48	Darbepoetin alfa 300 μg		Platinum, Etoposide-21	Small-Cell Lung Cancer	[31]
49	none		Platinum, Etoposide-21	Small-Cell Lung Cancer	[31]
50	none	none	CHOP-21*	NHL	[32, 33]
51	none	none	CHOEP-21*	NHL	[32, 33]
52	none	none	BEACOPP-21*	HD	[34]
53	none	Fil. 480 μg, d 4-13	CHOP-14*	NHL	[32, 33]
54	none	Fil. 480 μg, d 6-12	CHOP-14*	NHL	[35]
55	none	Fil. 480 μg, d 4-13	CHOEP-14*	NHL	[32, 33]
56	none	Fil. 480 μg, d 6-13	high-CHOEP-21*	NHL	[36]
57	none	Fil. 480 μg, d 6-13	high-CHOEP-14*	NHL	[37]
58	none	Fil. 480 μg, d 8-15	BEACOPP -21 escalated*	HD	[34]
59	none	Fil., 480 μg, d 3-10	E-T-C*	breast cancer	[30]
60	none	none	EC-T*	breast cancer	[30]
61	none	Fil. 5 x 5 μg/kg	none	NSCLC	[38]
62	none	Fil. 1 x 3 μg/kg	none	none	[39]
63	none	Fil. 1 x 5 μg/kg	none	none	[39]
64	none	Fil. 1 x 10 μg/kg	none	none	[39]
65	none	Fil. 1 x 5 μg/kg	none	none	[40]
66	none	Fil. 1 x 10 μg/kg	none	none	[40]
67	none	Fil. 10 x 75 μg	none	none	[41]
68	none	Fil. 10 x 150 μg	none	none	[41]
69	none	Fil. 10 x 300 μg	none	none	[41]
70	none	Fil. 10 x 600 μg	none	none	[41]
71	none	Fil. 14 x 30 μg	none	none	[42]
72	none	Fil. 14 x 300 μg	none	none	[42]
73	none	Fil. 4 μg/kg	none	none	[43]
74	none	Fil. 8 μg/kg	none	none	[43]
75	none	Fil. 5 μg/kg , d 1-13	none	none	[44]
76	none	Fil. 375 μg	none	none	[45]
77	none	Fil. 750 μg	none	none	[45]
78	none	Peg. 30 μg/kg	none	none	[46]
79	none	Peg. 60 μg/kg	none	none	[46]
80	none	Peg. 100 μg/kg	none	none	[46]
81	none	Peg. 300 μg/kg	none	none	[46]
82	none	Peg. 30 μg/kg	none	NSCLC	[38]
83	none	Peg. 100 μg/kg	none	NSCLC	[38]
84	none	Peg. 300 μg/kg	none	NSCLC	[38]
85	none	Fil. 5 μg/kg, d 2-13	TA	breast cancer	[47]
86	none	Peg. 30 μg/kg, d 2	TA	breast cancer	[47]
87	none	Peg. 60 μg/kg, d 2	TA	breast cancer	[47]
88	none	Peg. 100 μg/kg, d 2	TA	breast cancer	[47]
89	none	Peg. 6000 μg, d 2	TA	breast cancer	[48, 49]
90	none	Peg. 6000 μg, d 2	TA	breast cancer	[50]
91	none	Peg. 30 μg/kg, d 2	CP	NSCLC	[38]

92	none	Peg. 100 μg/kg, d 2	CP	NSCLC	[38]
93	none	Peg. 300 μg/kg, d 2	CP	NSCLC	[38]
94	none	Peg. 6000 μg, d 2	CHOP-14	NHL	[51]
95	none	Peg. 6000 μg, d 2	R CHOP-14	DLBCL	[52]
96	none	Peg. 6000 μg, d 2	R CHOP-14	DLBCL	[53]
97	none	Fil. 12x5 μg/kg	ESHAP	HD or NHL	[54]
98	none	Peg., 6000 μg, d 2	CHOP-14*	NHL	[55]
99	none	Peg., 6000 μg, d 4	CHOP-14*	NHL	[55]
100	none	Peg., 100 μg/kg, d 6	ESHAP	HD or NHL	[54]
101	none	Fil. 2.5 μg/kg	none	none	[56]
102	none	Fil. 5 μg/kg	none	none	[56]
103	none	Fil. 10 μg/kg	none	none	[56]
104	none	Fil. 5 μg/kg iv	none	none	[56]

Stability analysis

Using the above mentioned model parameters results in a stable steady state of the system. However, modifying certain parameters can result in stable oscillations. This especially applies for parameters of the long range stem cell feedback (see figure 3).

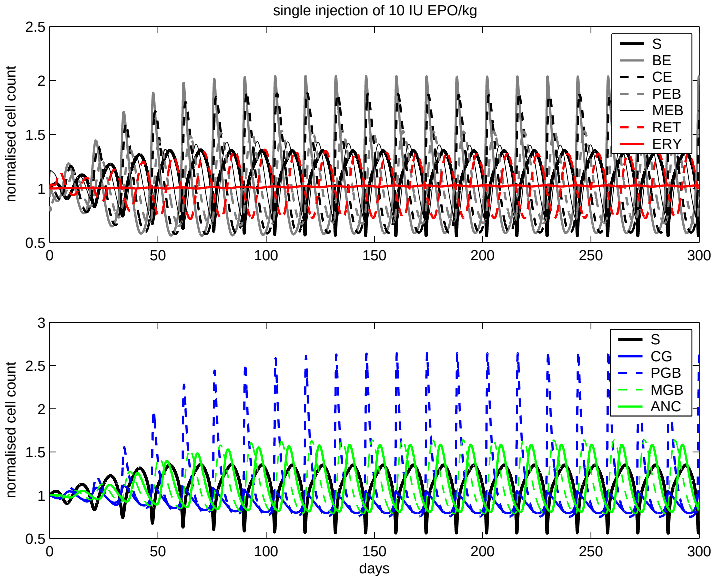

Figure 3: **Oscillatory behaviour of the model for changed parameter settings of the stem cell feedback:** A small perturbation (a single injection of 10 IU EPO at $t = 0$) leads to stable oscillations in compartment sizes. Modified parameter settings: $a_S^{\text{min}} = 0.01, a_S^{\text{nor}} = 0.05105, a_S^{\text{int}} = 0.71047, a_S^{\text{max}} = 1, p_\delta = 0.40858$ We present cell dynamics normalised to steady state values.

Sensitivity analysis

We analysed the sensitivity of newly introduced parameters by changing their values by 2.5% and calculating the corresponding deterioration of the fitness function. Results are shown in figure 4. Minimum and normal values of the G-CSF regulations of compartment CE have considerably higher precision than corresponding estimates of maximum values and b-parameters.

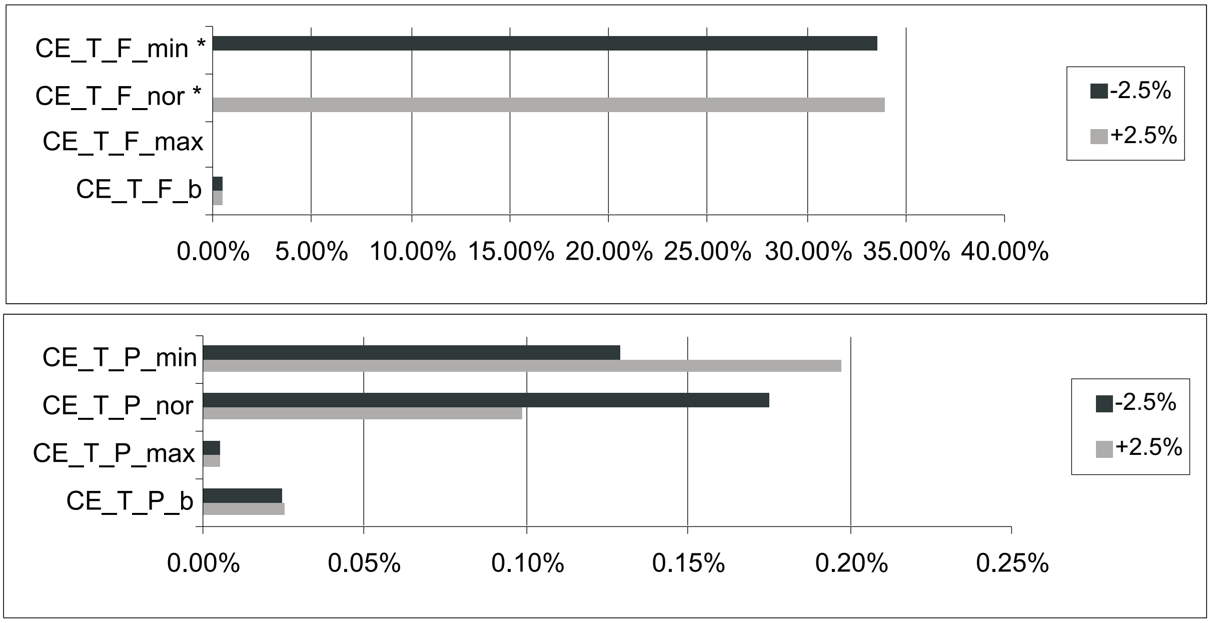

Figure 4: **Sensitivity of the parameters describing the regulation in compartment CE.** Parameters denoted with "*" could not be changed by 2.5 % without violating constraints. Parameters were modified by ±2.5%. Relative change of the fitness function is shown as length of corresponding bars.

A.9 Simulation Results

In this section, we present all simulation results of our hybrid model and compare it with available data. We also present extensive comparisons of our hybrid model with the single lineage models of erythropoiesis and granulopoiesis.

Comparison of hybrid model and single lineage models

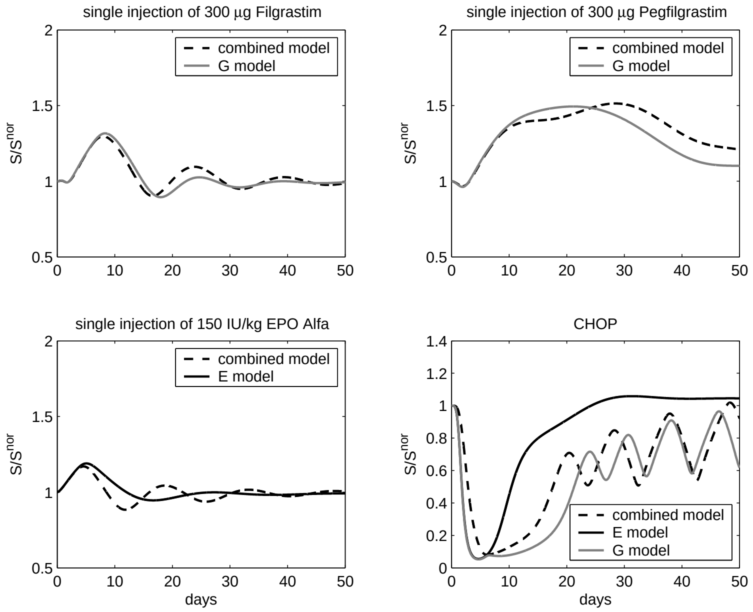

Figure 5: **Comparison of stem cell dynamics.** Comparison of the behaviour of the combined and the single lineage models after perturbation with EPO, Filgrastim, Pegfilgrastim and CHOP injections. We present stem cell dynamics normalised to steady state values.

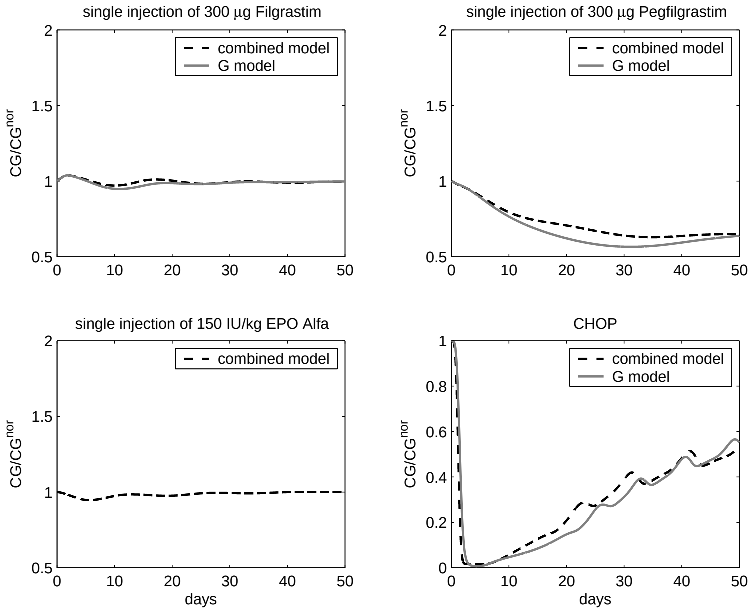

Figure 6: **Comparison of CG dynamics.** Comparison of the behaviour of the combined and the single lineage models after perturbation with EPO, Filgrastim, Pegfilgrastim and CHOP injections. We present CG dynamics normalised to steady state values.

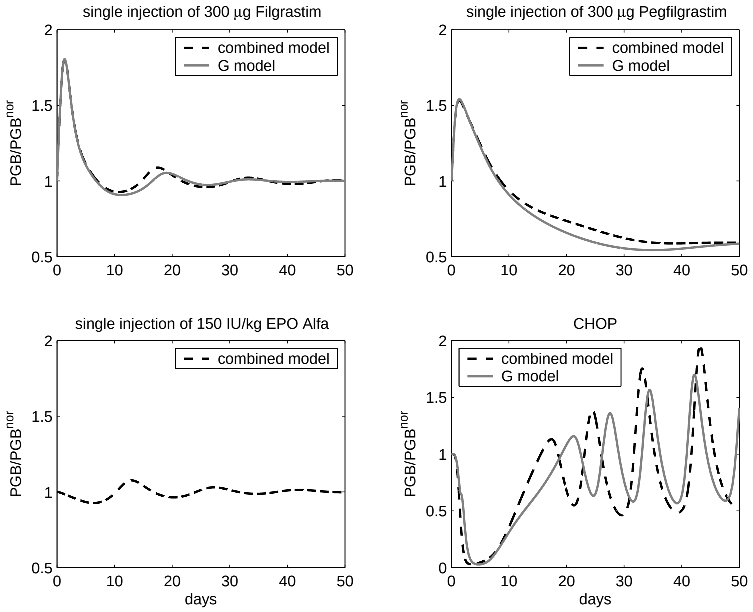

Figure 7: **Comparison of PGB dynamics.** Comparison of the behaviour of the combined and the single lineage models after perturbation with EPO, Filgrastim, Pegfilgrastim and CHOP injections. We present PGB dynamics normalised to steady state values.

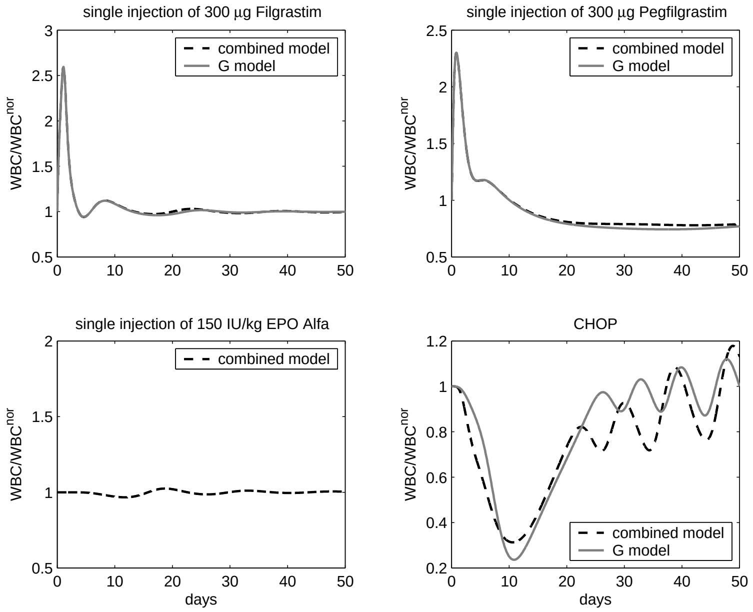

Figure 8: **Comparison of WBC dynamics.** Comparison of the behaviour of the combined and the single lineage models relative to steady state after perturbation with EPO, Filgrastim, Pegfilgrastim and CHOP injections (WBC).

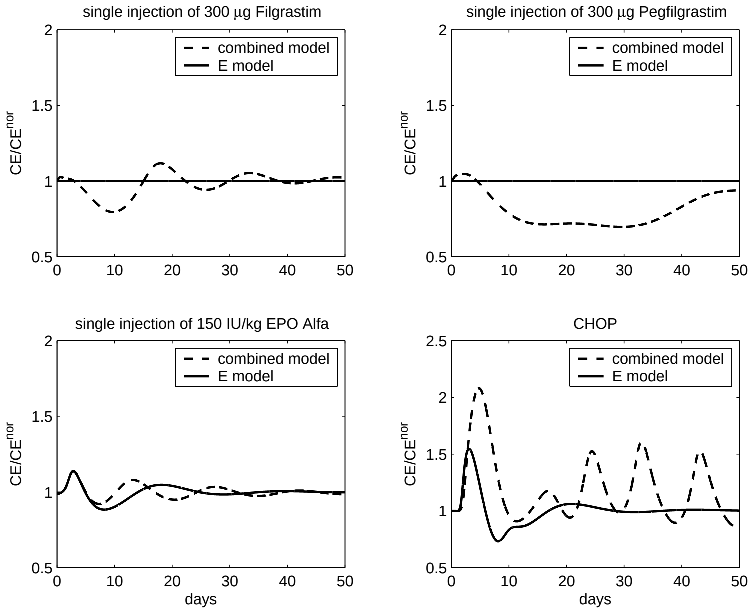

Figure 9: **Comparison of CE dynamics.** Comparison of the behaviour of the combined and the single lineage models relative to steady state after perturbation with EPO, Filgrastim, Pegfilgrastim and CHOP injections. We present CE dynamics normalised to steady state values.

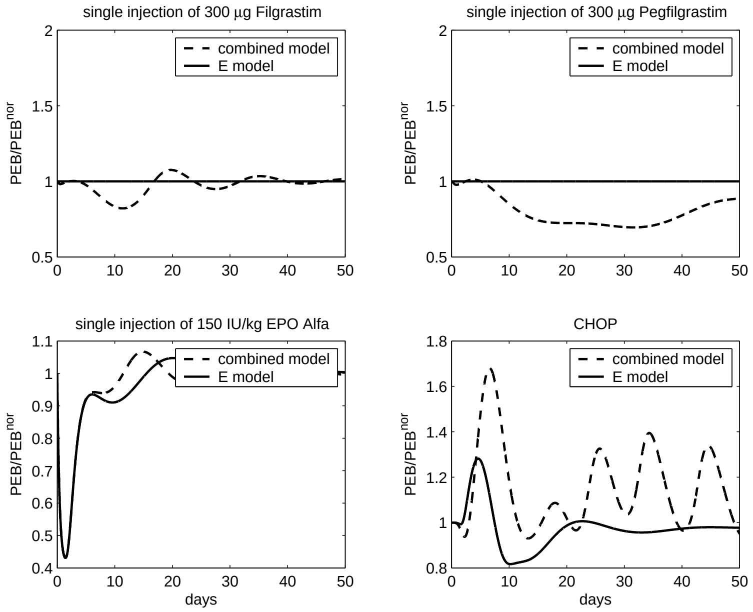

Figure 10: **Comparison of PEB dynamics.** Comparison of the behaviour of the combined and the single lineage models after perturbation with EPO, Filgrastim, Pegfilgrastim and CHOP injections. We present PEB dynamics normalised to steady state values.

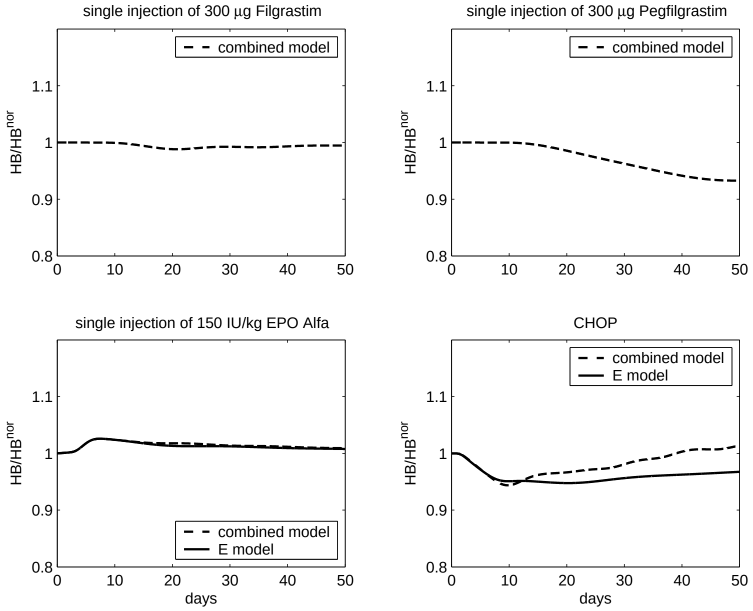

Figure 11: **Comparison of HB dynamics.** Comparison of the behaviour of the combined and the single lineage models relative to steady state after perturbation with EPO, Filgrastim, Pegfilgrastim and CHOP injections (HB).

A.9.1 Simulation Results of EPO applications into healthy volunteers

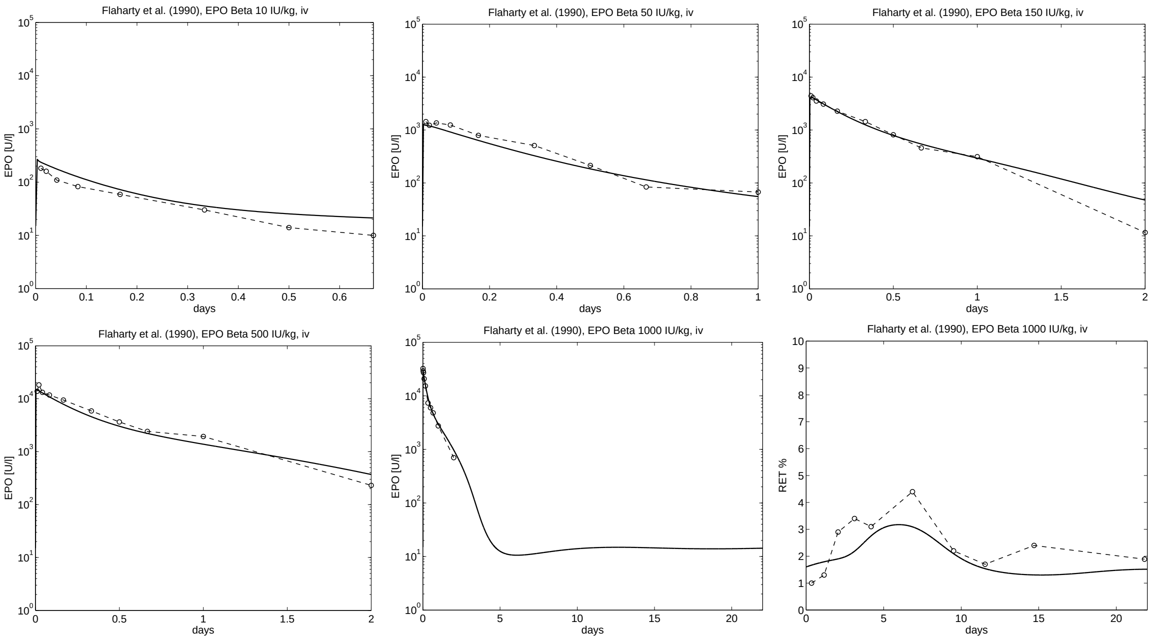

Figure 12: Serum concentration of erythropoietin, percentage of reticulocytes, simulation (black line) and data (circle), data: [23, 24]

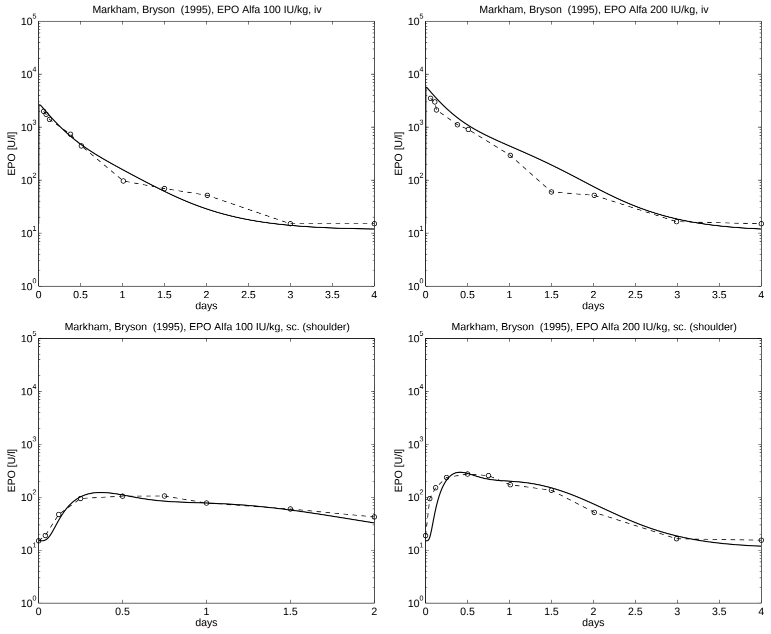

Figure 13: Serum concentration of erythropoietin (simulation and data), data: [20]

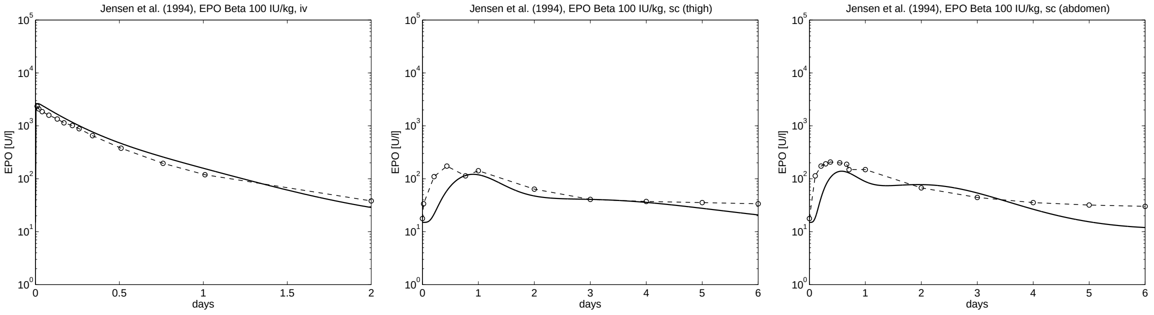

Figure 14: Serum concentration of erythropoietin, simulation (black line) and data (circle), data: [27]

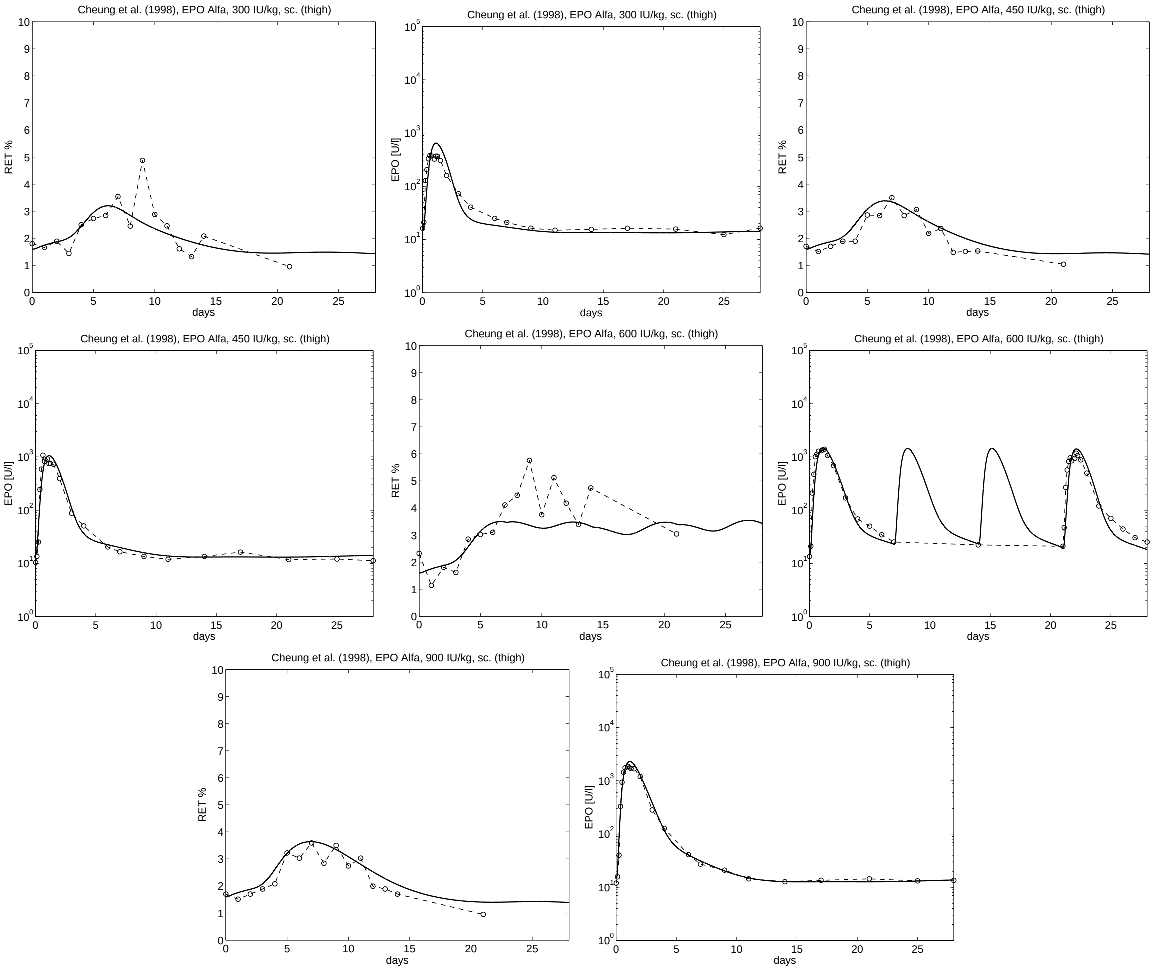

Figure 15: Serum concentration of erythropoietin and reticulocytes %, simulation (black line) and data (circle), data: [16]

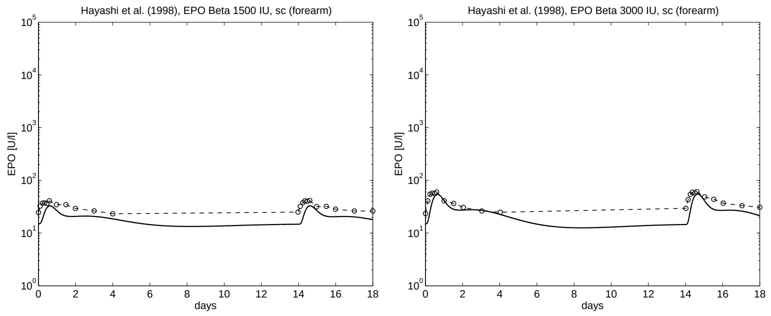

Figure 16: Serum concentration of erythropoietin (simulation and data), data: [26]

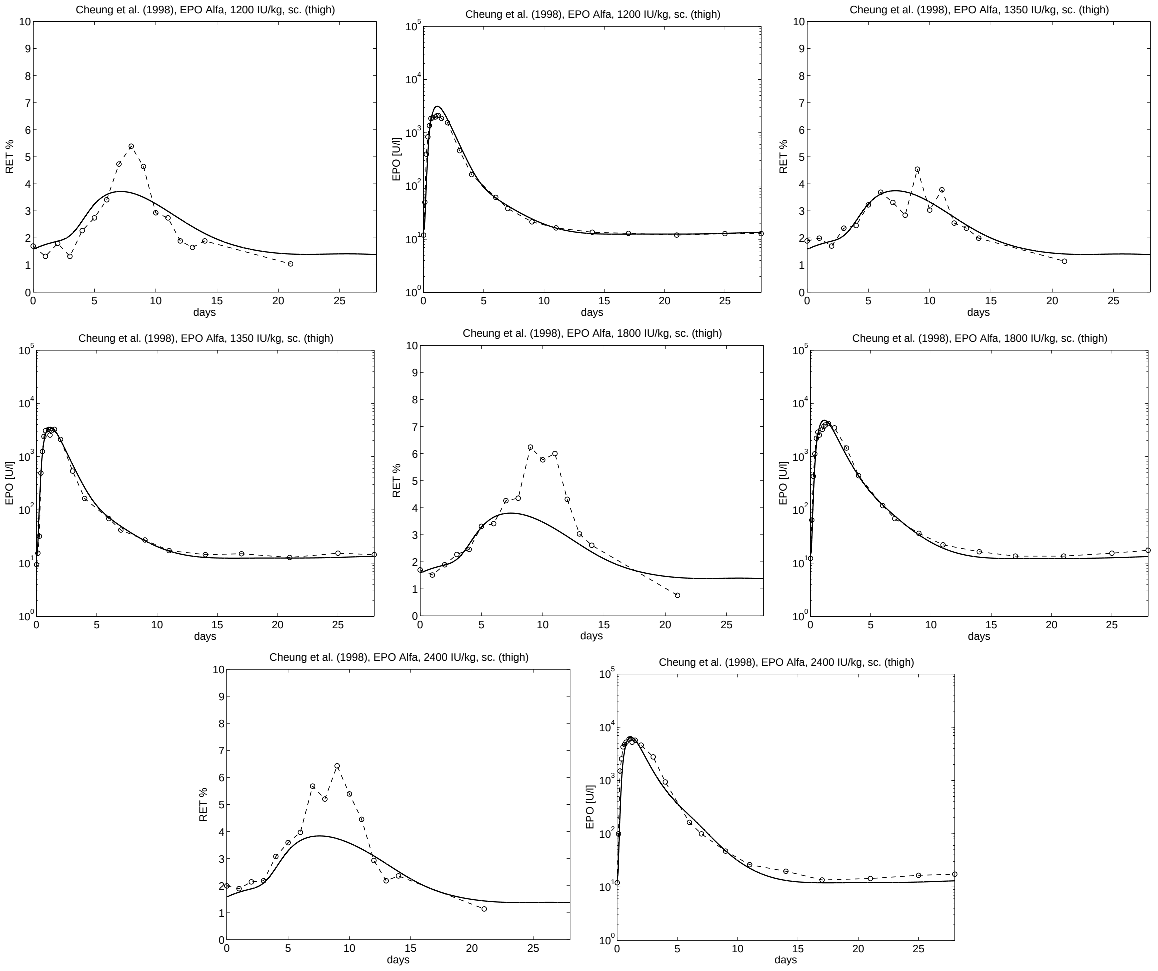

Figure 17: Serum concentration of erythropoietin and reticulocytes %, simulation (black line) and data (circle), data: [16]

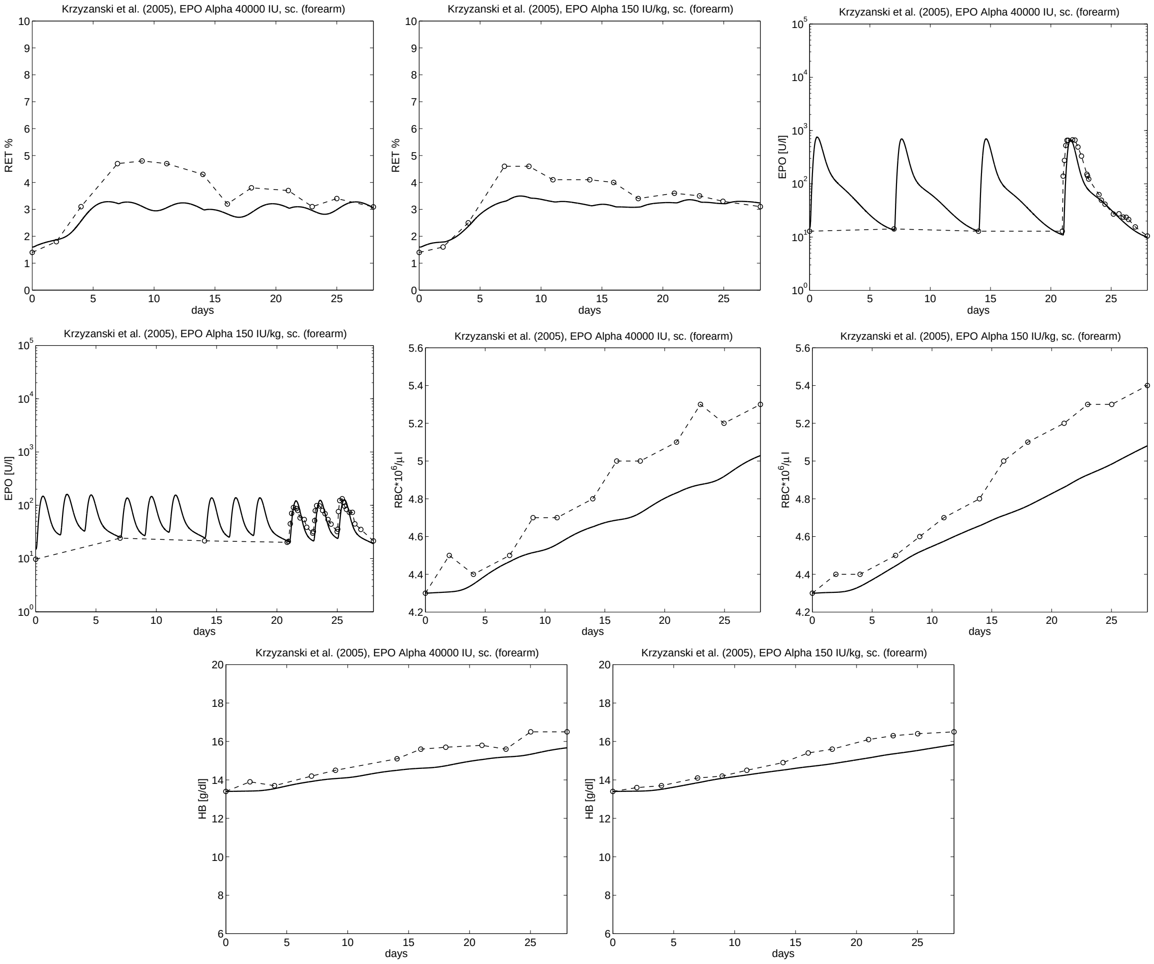

Figure 18: Serum concentration of erythropoietin, HB value, RBC, and reticulocytes %, simulation (black line) and data (circle), data: [9]

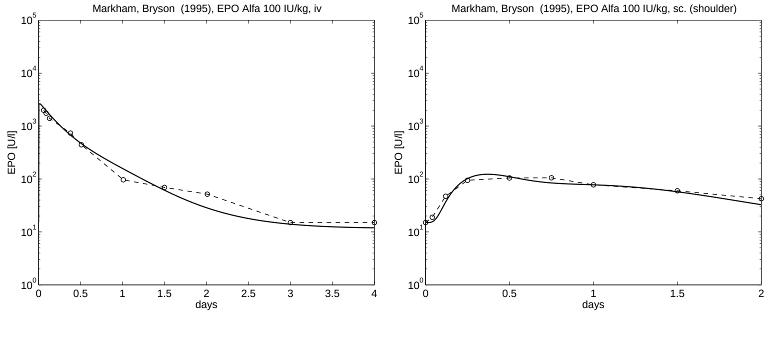

Figure 19: Intravenous injection (left) and subcutaneous injection (right): serum concentration of erythropoietin, Alfa iv, Alfa sc, data: [20]

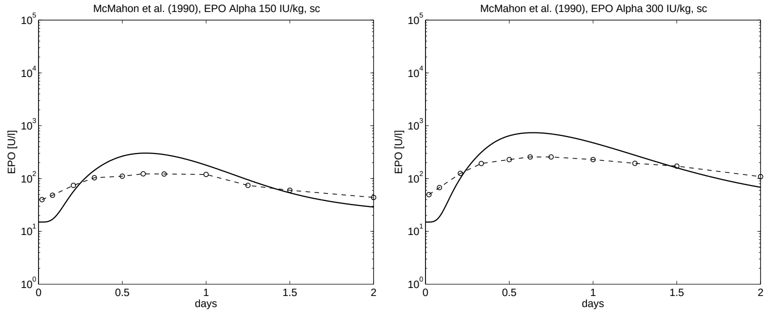

Figure 20: Serum concentration of erythropoietin, simulation (black line) and data (circle), data: [19]

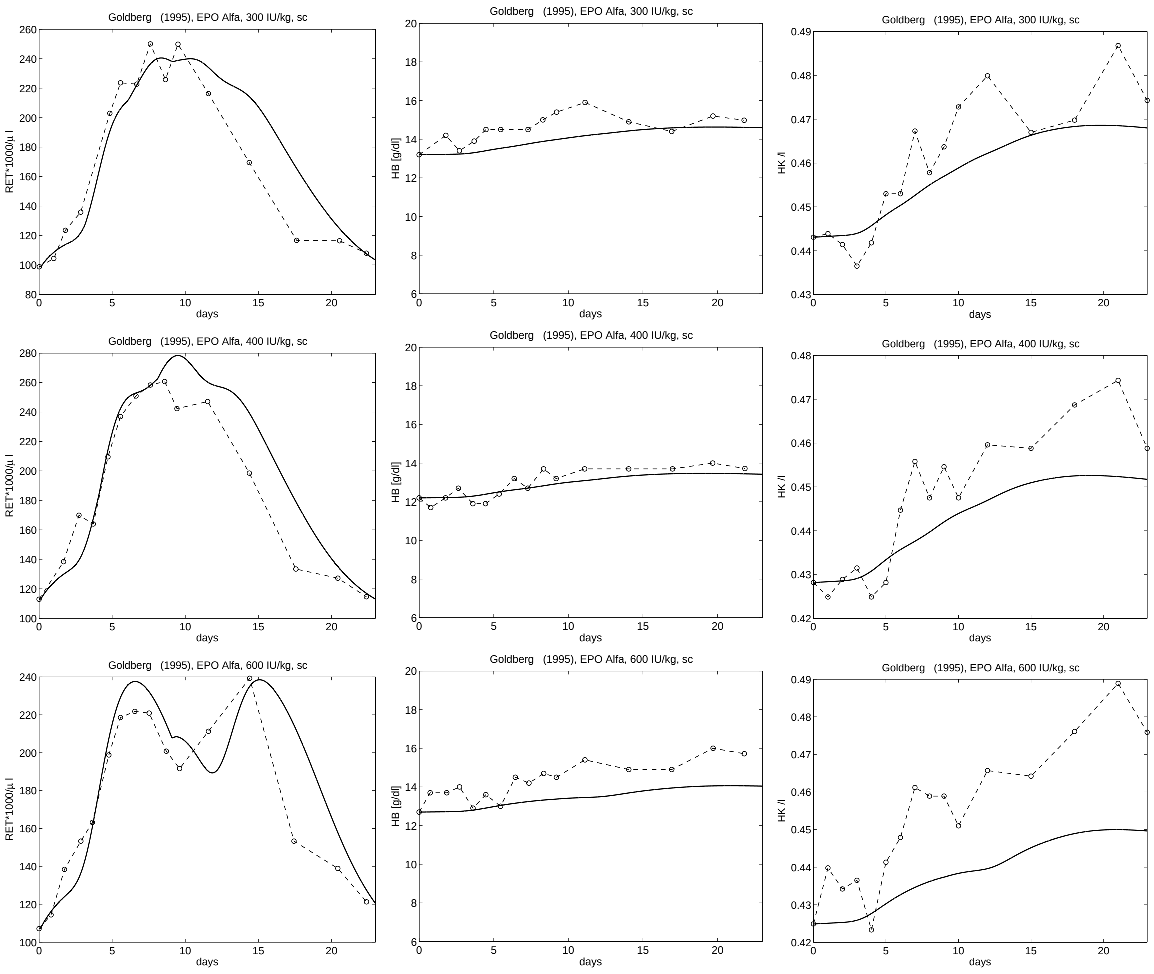

Figure 21: Serum concentration of erythropoietin, HB and HK value, simulation (black line) and data (circle), data: [17], [57], from subjects required to have a baseline HK less than 48%. If the HK rose above 55%, phlebotomy was performed.

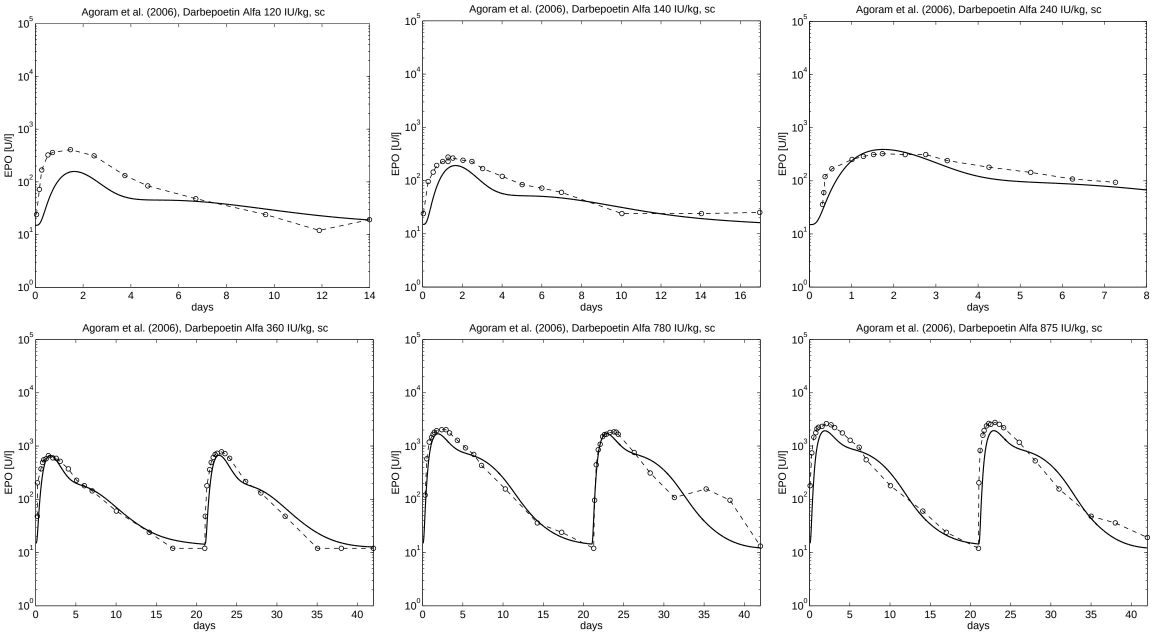

Figure 22: Serum concentration of erythropoietin, simulation (black line) and data (circle), data: [29]

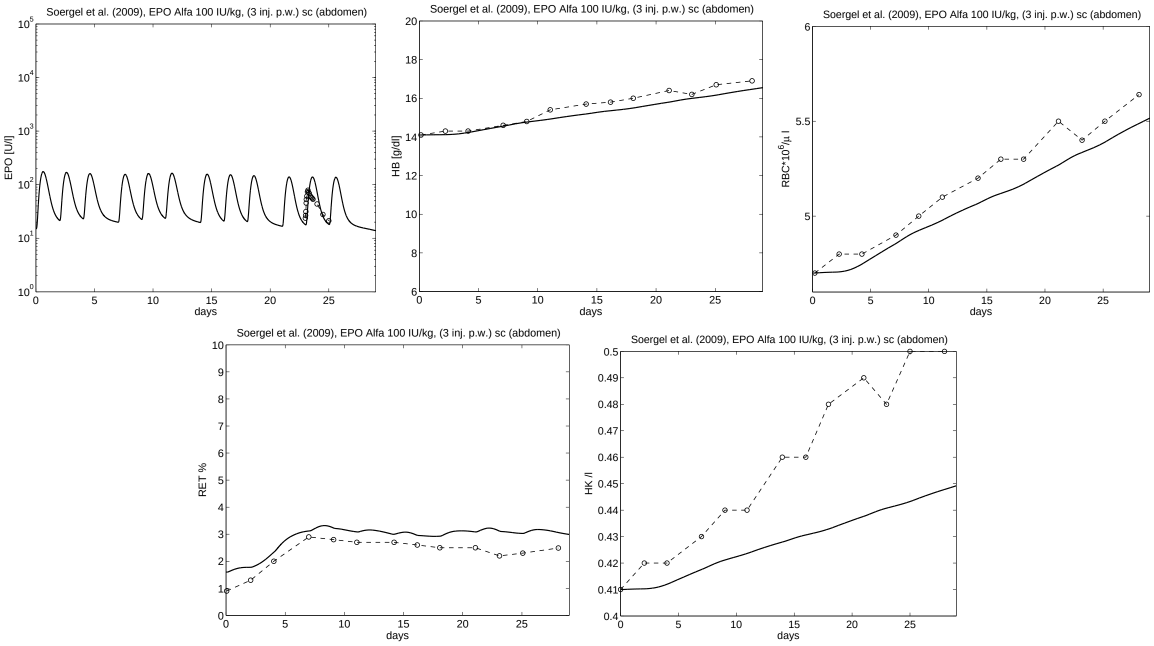

Figure 23: Serum concentration of erythropoietin, HB, RBC, and percentage of reticulocytes, simulation (black line) and data (circle), data: [22]

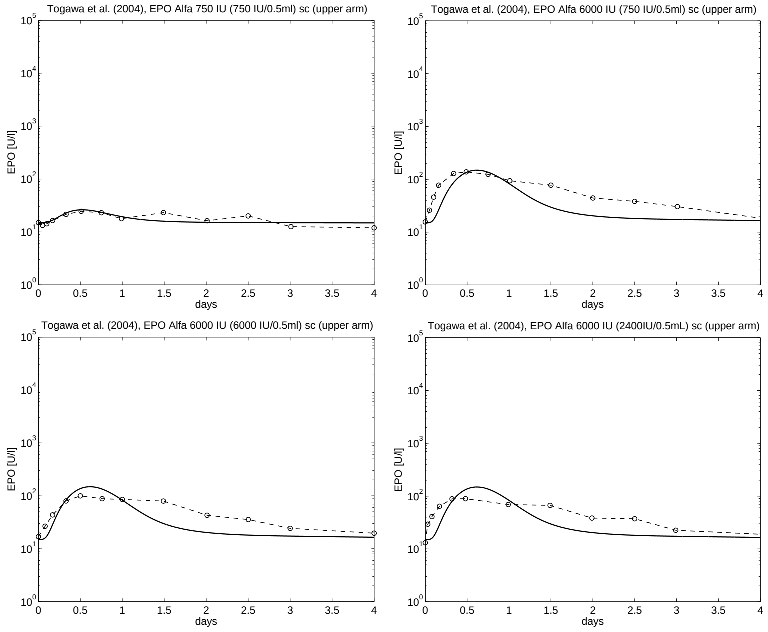

Figure 24: Serum concentration of erythropoietin, simulation (black line) and data (circle), data: [18]

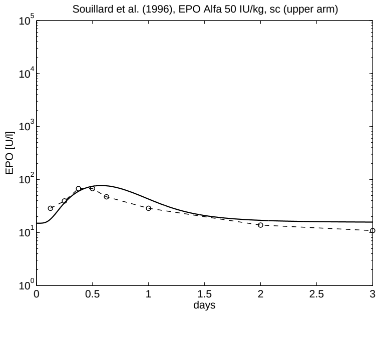

Figure 25: Serum concentration of erythropoietin, simulation (black line) and data (circle), data: [21]

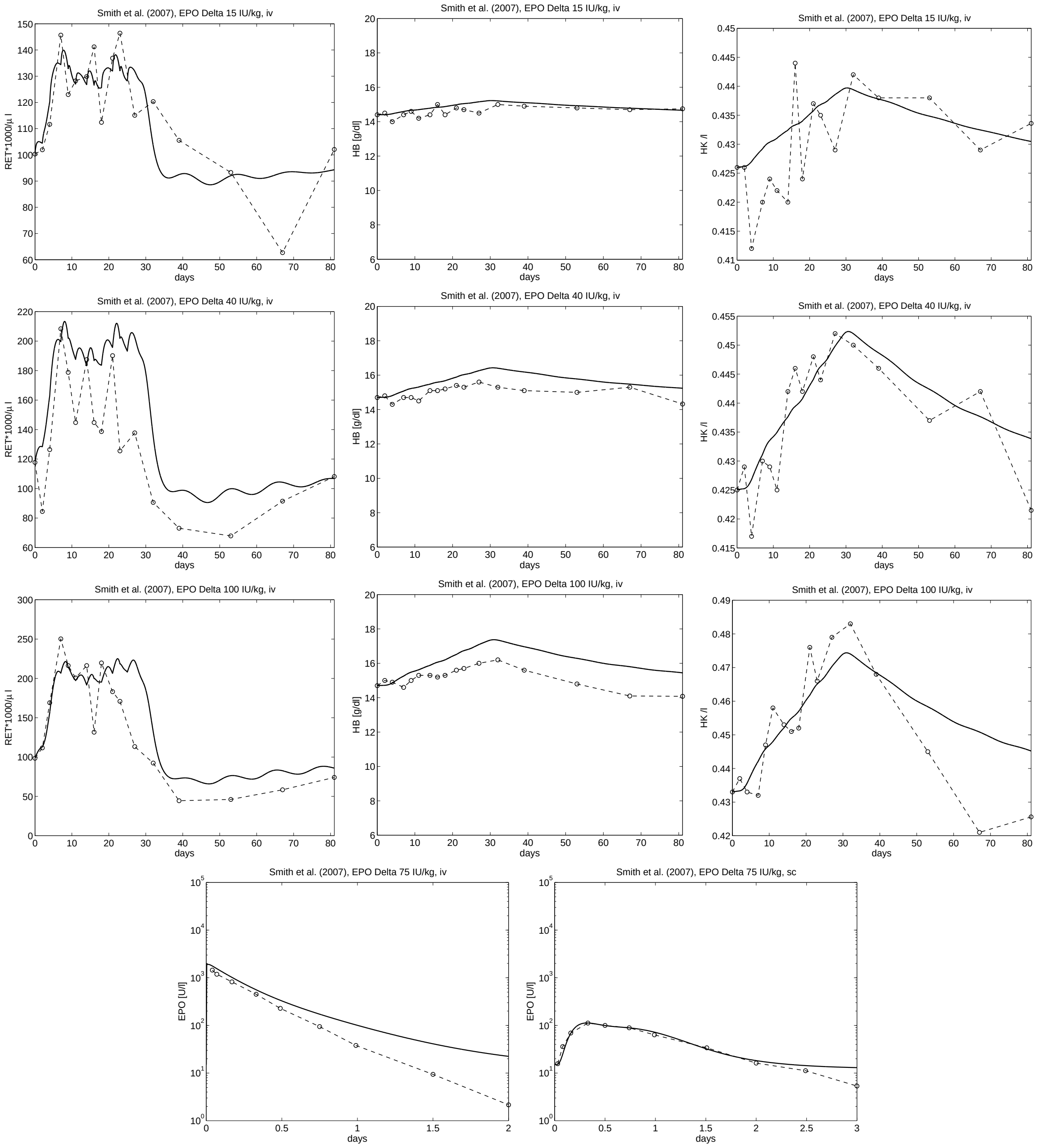

Figure 26: reticulocytes, HB, HK and serum concentration of erythropoietin, simulation (black line) and data (circle), data: [28]

A.9.2 Simulation results of scenarios with combined EPO and chemotherapy applications

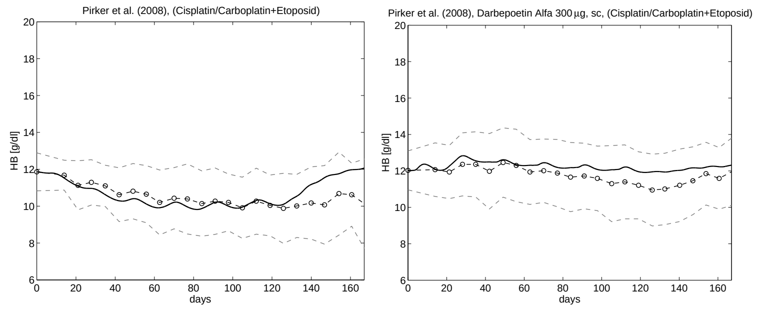

Figure 27: HB value: simulation (black line) and data (circle), mean ± standard deviation (grey line), Chemotherapy: Platinum plus Etoposide, without EPO support (left), with Darbepoetin alfa, sc (right), data: [31]

A.9.3 Simulation results of G-CSF injections into healthy volunteers

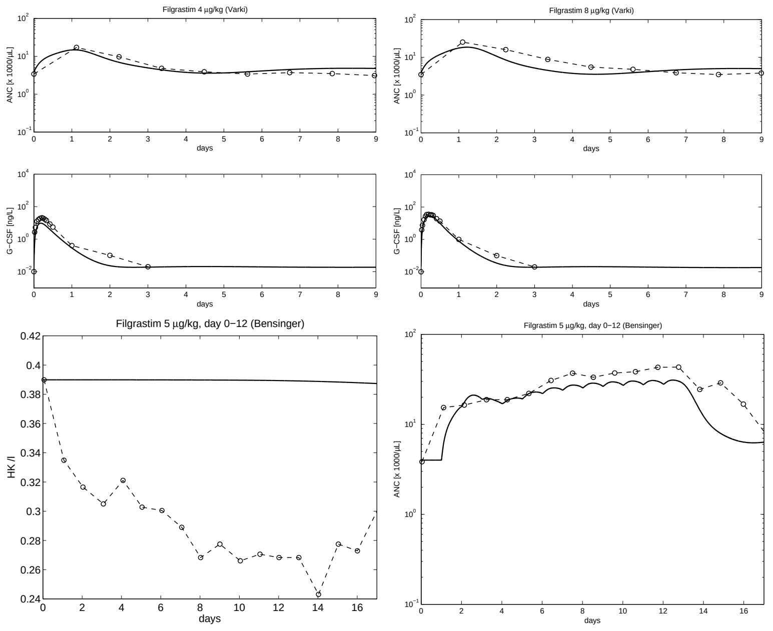

Figure 28: simulation (black line) and data (circle), data: [43, 44]. The blood cell donation in the scenario of Bensinger et al. [44] is not taken into account.

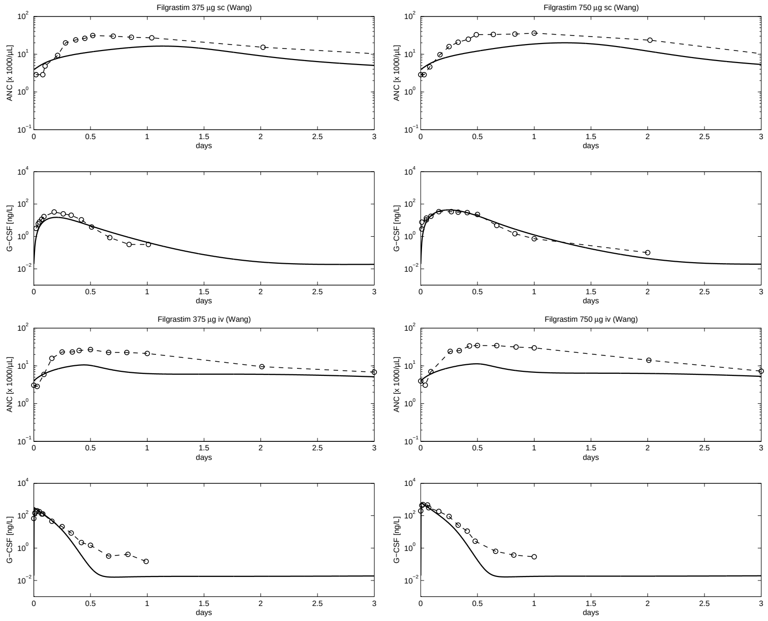

Figure 29: simulation (black line) and data (circle), data: [45]

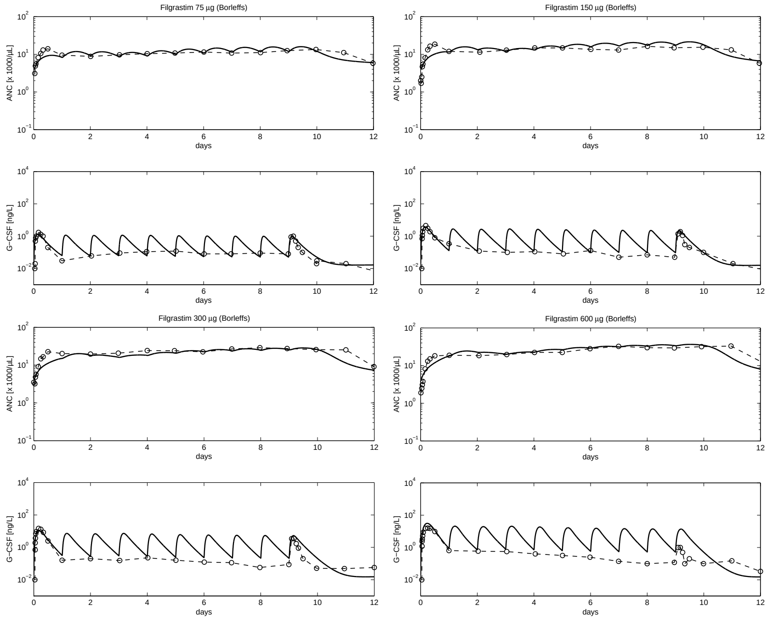

Figure 30: simulation (black line) and data (circle), data: [41]

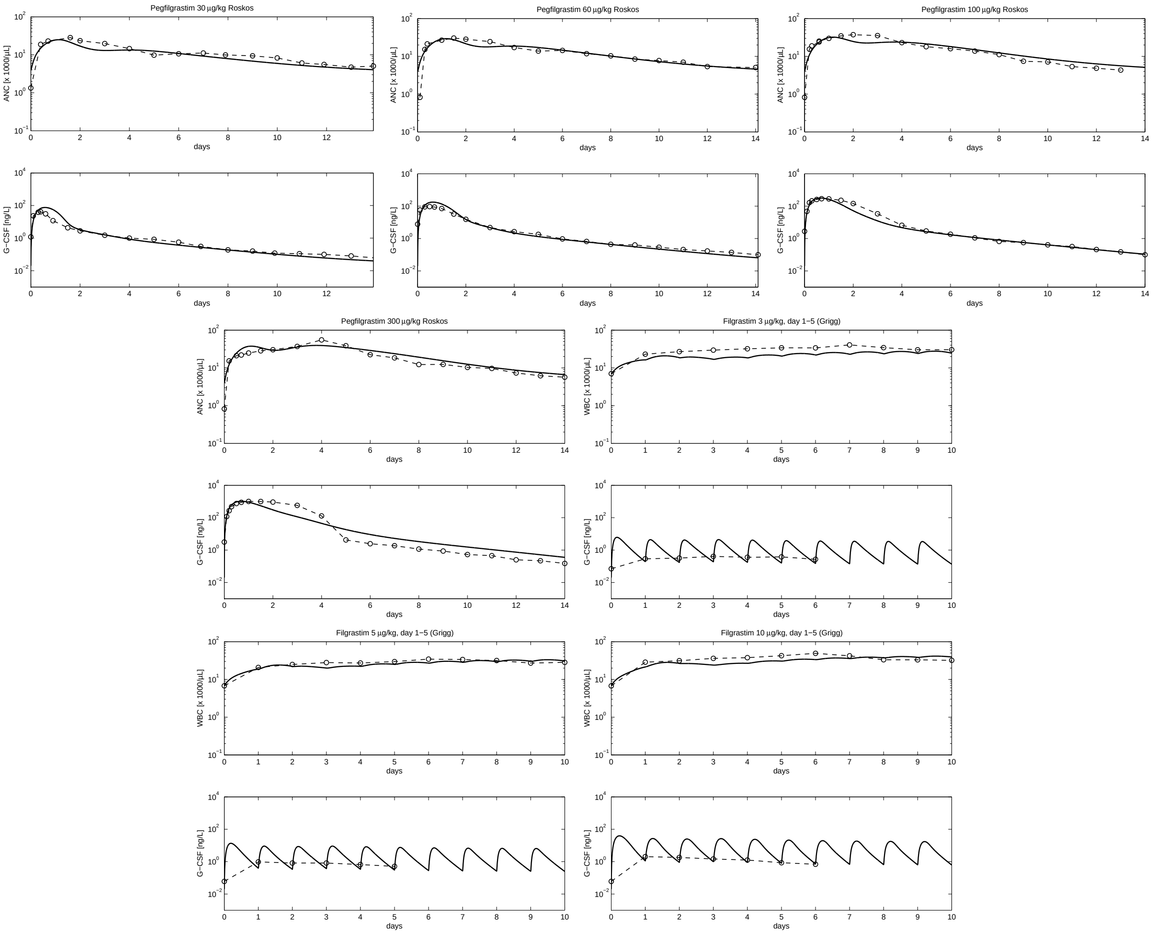

Figure 31: simulation (black line) and data (circle), data: [39, 46]

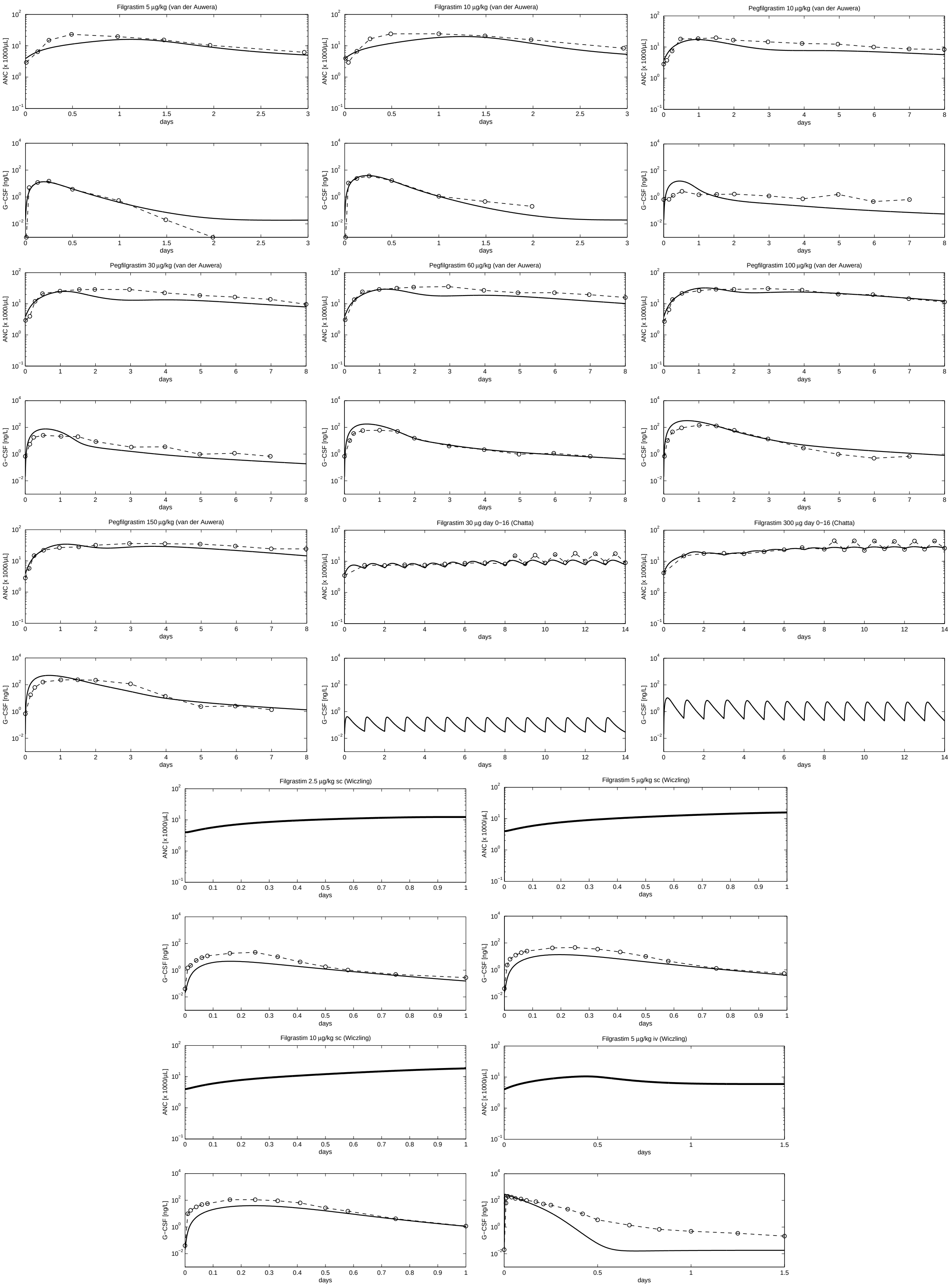

Figure 32: simulation (black line) and data (circle), data: [40, 42, 56]

A.9.4 Simulation results of scenarios with combined G-CSF and chemotherapy application

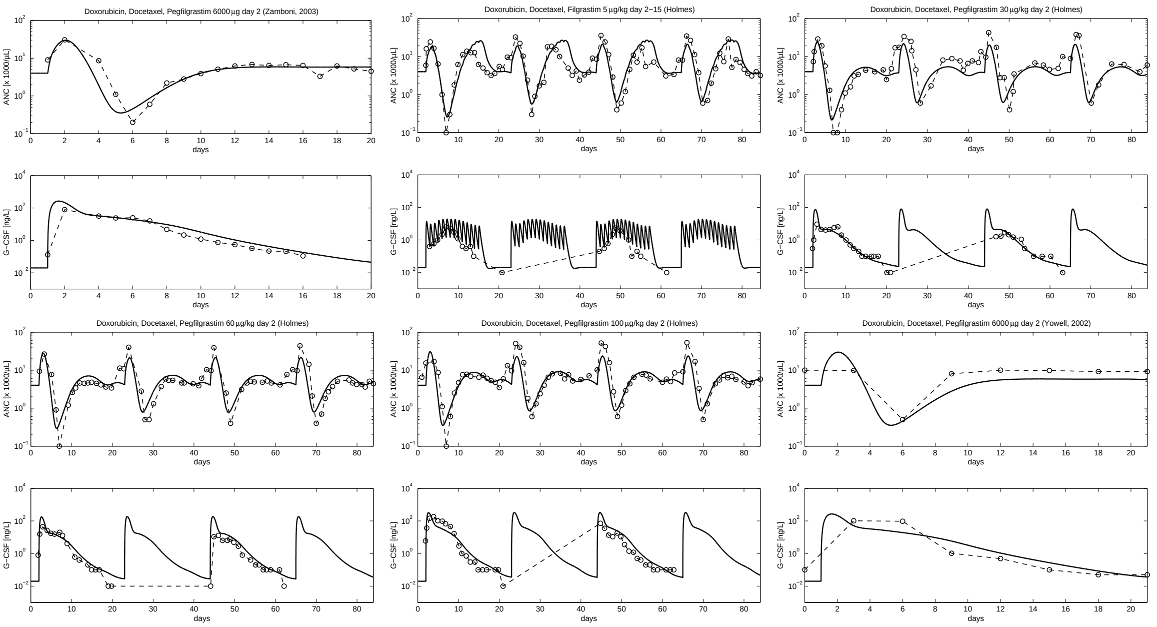

Figure 33: simulation (black line) and data (circle), data: [47, 49, 50]

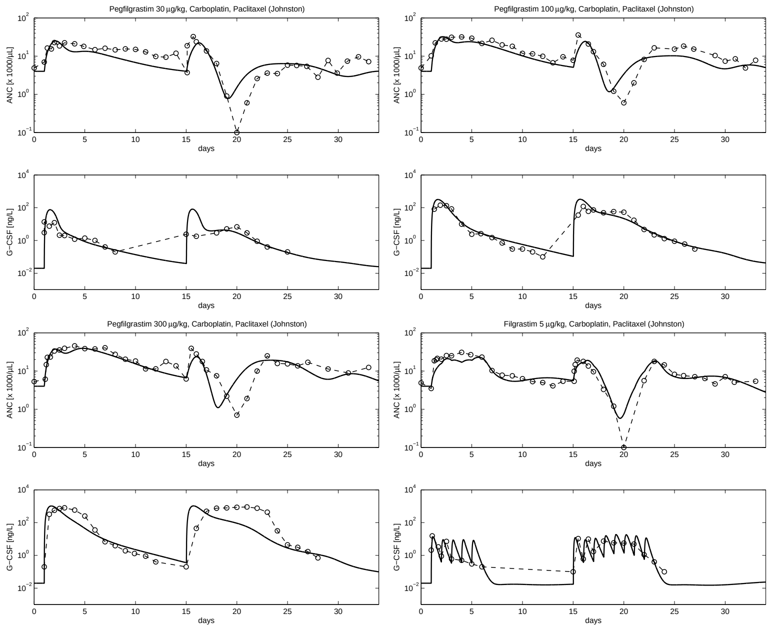

Figure 34: simulation (black line) and data (circle), data: [38]

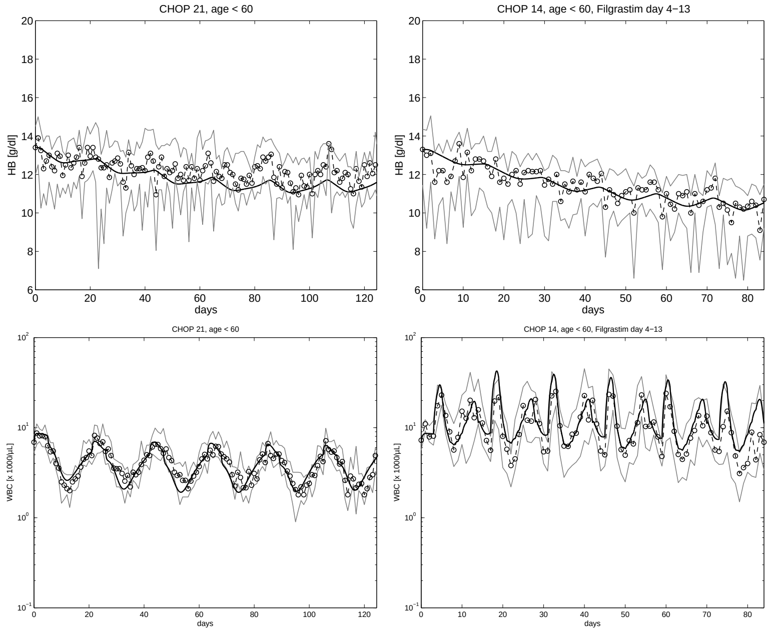

Figure 35: HB value, simulation (black line) and data (circle), percentile 25, 75 (grey line), Chemotherapy: CHOEP 21, CHOEP14, elderly patients, CHOP 21 younger patients, data: [33].

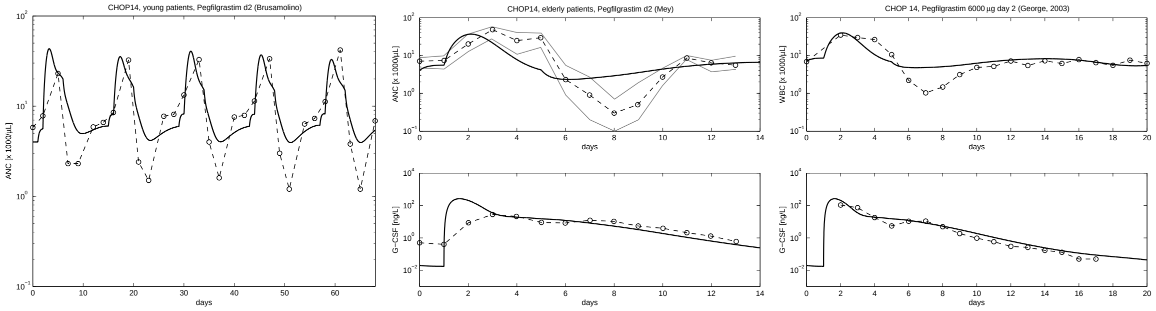

Figure 36: simulation (black line) and data (circle), data: [51–53].

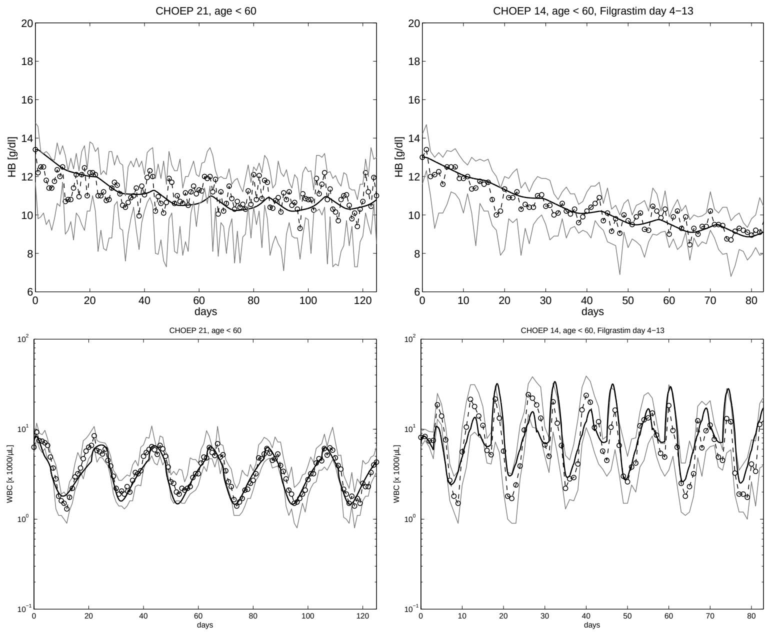

Figure 37: HB value, simulation (black line) and data (circle), percentile 25, 75 (grey line), Chemotherapy: CHOP14, CHOEP 21, CHOEP14, younger patients, data: [33].

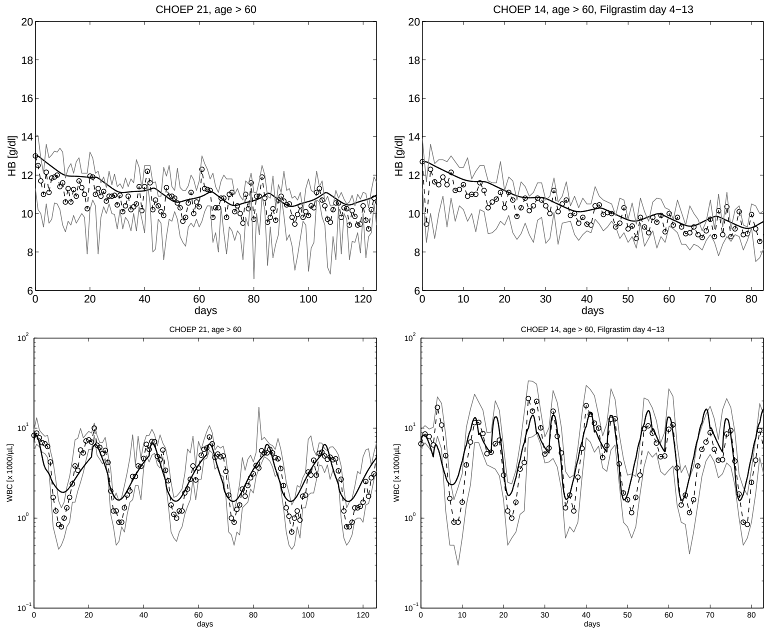

Figure 38: HB value, simulation (black line) and data (circle), percentile 25, 75 (grey line), Chemotherapy: CHOEP 21, CHOEP14, elderly patients, data: [32].

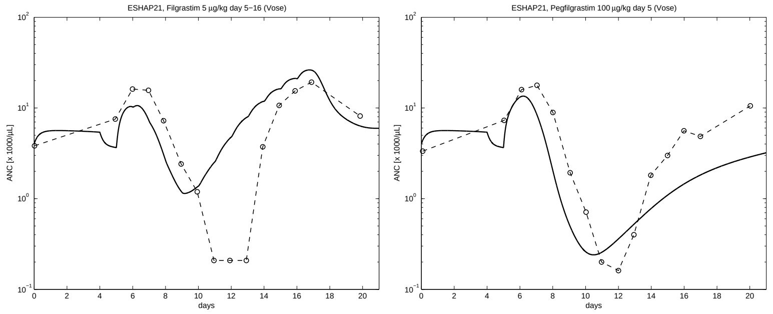

Figure 39: simulation (black line) and data (circle), data: [54]

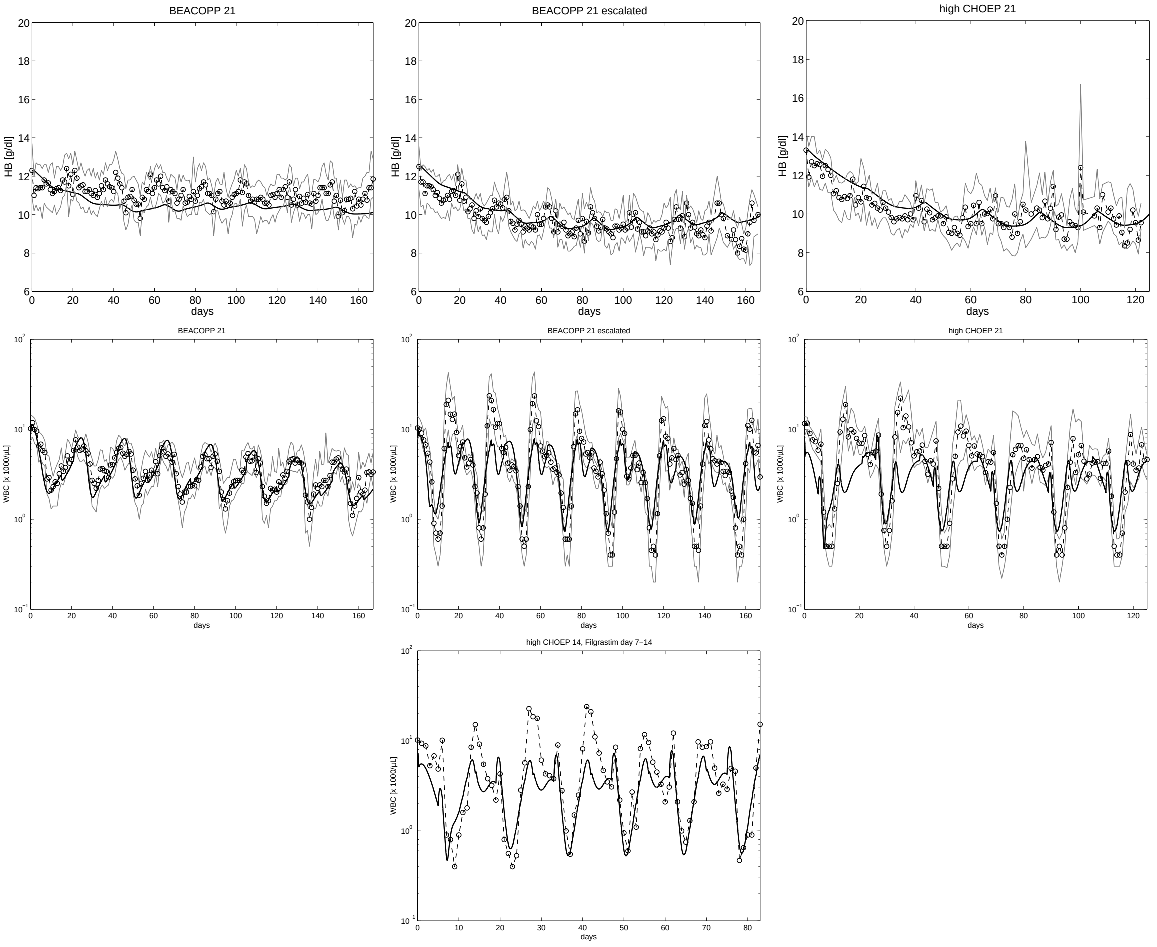

Figure 40: HB value, simulation (black line) and data (circle), percentile 25, 75 (grey line), Chemotherapy: BEACOPP 21 escalated, BEACOPP 21, highCHOEP 21, highCHOEP 14, data: [34, 36, 37, 58]. Caused by Hodgkins lymphoma activity, an increased endogenous G-CSF production is assumed in BEACOPP treated patients, see [2].

References

1. Wichmann H, Loeffler M: *Mathematical modeling of cell proliferation: Stem cell regulation in hemopoiesis, Vol. 1, 2.* CRC Press 1985.
2. Scholz M, Engel C, Loeffler M: **Modelling Human Granulopoiesis under Polychemotherapy with G-CSF Support.** *J Math Biol* 2005, **50**(4):397–439.
3. Scholz M, Schirm S, Wetzler M, Engel C, Loeffler M: **Pharmacokinetic and -dynamic modelling of G-CSF derivatives in humans.** *Theoretical Biology and Medical Modelling* 2012, **9:32, doi:10.1186/1742-4682-9-32**.
4. Schirm S, Scholz M, Loeffler M, Engel C: **A Biomathematical Model of Human Erythropoiesis under Erythropoietin and Chemotherapy Administration.** *PLoS ONE* 2013, **8**(6, e65630):doi:10.1371/journal.pone.0065630.
5. Loeffler M, Pantel K, Wulff H, Wichmann H: **A mathematical model of erythropoiesis in mice and rats. Part 1: Structure of the model.** *Cell Tissue Kinet* 1989, **22**:13–30.
6. Pantel K: *Erweiterung eines kybernetischen Modelles der Erythropoese und dessen Anwendung für normale und pathologische Mäuse. Dissertation, Universität zu Köln (in german)* 1987.
7. Wichmann H: *Computer modeling of erythropoiesis. In: Current Concepts in Erythropoiesis, Chapter V.*, John Wiley and Sons 1983 .
8. Kota J, Machavaram K, McLennan D, Edwards G, Porter C, et al: **Lymphatic Absorption of Subcutaneously Administered Proteins: Influence of Different Injection Sites on the Absorption of Darbepoetin Alfa Using a Sheep Model.** *Drug Metab Dispos* 2007, **35**(12):2211–2217.
9. Krzyzanski W, Wyska E: **Pharmacokinetics and pharmacodynamics of erythropoietin receptor in healthy volunteers.** *Naunyn-Schmiedeberg's Arch Pharmacol* 2008, **377**:637–645.
10. Sawada K, Krantz S, Kans J, Dessypris E, Sawyer S, et al: **Purification of Human Erythroid Colony-forming Units and Demonstration of Specific Binding of Erythropoietin.** *J Clin Invest* 1987, **80**:357–366.
11. Mackey M, Aprikyan A, Dale D: **The rate of apoptosis in post mitotic neutrophil precursors of normal and neutropenic humans.** *Cell Prolif* 2003, **36**:27–34.
12. Scholz M, Engel C, Loeffler M: **Model-based design of chemotherapeutic regimens that account for heterogeneity in leucopoenia.** *Br J Haematol* 2006, **132**(6):723–735.
13. Schmitz S, Franke H, Loeffler M, Wichmann H, Diehl V: **Model analysis of the contrasting effects of GM-CSF and G-CSF treatment on peripheral blood neutrophils observed in three patients with childhood-onset cyclic neutropenia.** *Brit J Haematol* 1996, **95**:616–625.
14. Franke M, Schmitz S: *Modell Mensch aus Übersicht über die Modelle zur Hämatopoese 1979-1993.* Universität zu Köln 1993.
15. Krzyzanski W, Jusko W, Wacholtz M, Minton N, Cheung W: **Pharmacokinetic and pharmacodynamic modeling of recombinant human erythropoietin after multiple subcutaneous doses in healthy subjects.** *Eur J Pharm Sci* 2005, **26**:295–306.
16. Cheung W, Goon B, Guilfoyle M, Wachholtz M: **Pharmacokinetics and Pharmacodynamics of recombinant human erythropoietin after single and multiple subcutaneous doses to healthy subjects.** *Clin Pharmacol Ther* 1998, **8**(4):309–317.
17. Goldberg M: **Erythropoesis, Erythropoetin, and Iron Metabolism in Elective Surgery: Preoperative Strategies for Avoiding Allogeneic Blood Exposure.** *Am J Surg 170* 1995, (6a):37S–43S.
18. Togawa A, Tanaka T, Nagashima S, Keta H, Kobayashi Y, et al: **A comparison of the bioequivalence of two formulations of epoetin alfa after subcutaneous injection.** *Br J Clin Pharmacol* 2004, **58**(3):269–276.
19. McMahon F, Vargas R, Ryan M, Jain A, Abels R, et al: **Pharmacokinetics and effects of recombinant human erythropoietin after intravenous and subcutaneous injections in healthy voluteers.** *Blood* 1990, **76**:1718–1722.
20. Markham A, Bryson H: **Epoetin alfa. A review of its pharmacodynamic and pharmacokinetic properties and therapeutic use in nonrenal applications.** *Drugs* 1995, **49**(2):232–254.

21. Souillard A, Audran M, Bressolle F, Gareau R, Duvallet A, Chanal J: **Recombinant human erythropoietin and pharmacodynamic parameters in athletes. Interest of blood sampling for doping control.** *Br J Clin Pharmacol* 1996, **42**(51):355–360.

22. Sörgel F, Thyroff-Friesinger U, Vetter A, Vens-Cappell B, Kinzig M: **Bioequivalence of HX575 (Recombinant Human Epoetin Alfa) and a Comparator Epoetin Alfa after Multiple Subcutaneous Administrations.** *Pharmacology* 2009, **38**:122–130.

23. Flaharty K, Caro J, Erslev A, Whalen J: **Pharmakokinetics and erythropoietic response to human recombinant erythropoietin in healthy men.** *Clin Pharmacol Ther* 1990, **47**(5):557–564.

24. Flaharty K: **Clinical Pharmacology of Recombinant Human Erythropoietin (r-HuEPO).** *Pharmacotherapy* 1990, **10**(2 Pt 2):9S–14S.

25. Lundby C, Thomsen J, Boushel R, Koskolou M, Warberg J, et al: **Erythropoietin treatment elevates haemoglobin concentration by increasing red cell volume and depressing plasma volume.** *J Physiol* 2007, **578**:309–314.

26. Hayashi N, Kinoshita H, Yukawa E, Higuchi S: **Pharmacokinetic analysis of subcutaneous erythropoietin administration with nonlinear mixed effect model including endogenous production (7).** *Br J Clin Pharmacol* 1998, **46**:11–19.

27. Jensen J, Madsen J, Jensen L, Pedersen E: **Reduction absorption and elimination of erythropoietin in uremia compared with healthy volunteers.** *J Am Soc Nephrol* 1994, **5**:177–185.

28. Smith W, Dowell J, Pratt R: **Pharmacokinetics and Pharmacodynamics of Epoetin Delta in Two Studies in Healthy Volunteers and Two Studies in Patients with Chronic Kidney Disease.** *Clinical Therapeutics* 2007, **29**(7):1368–1380.

29. Agoram B, Sutjandra L, Sullivan J: **Population pharmacokinetics of darbepoetin alfa in healthy subjects.** *Br J Clin Pharmacol* 2006, **63**:41–52.

30. GBG: **German Breast Group, Available: http://www.germanbreastgroup.de /studien /adjuvant /gain/unterlagen.html Accessed 2011 October 11**.

31. Pirker R, Ramlau R, Schuette W, Zatloukal P, Ferreira I, et al: **Safety and Efficacy of Darbepoetin Alfa in Previously Untreated Extensive-Stage Small-Cell Lung Cancer Treated With Platinum Plus Etoposide.** *J Clin Oncology* 2008, **26**.

32. Pfreundschuh M, Truemper L, Kloess M, Schmits R, Feller A, et al: **2-weekly or 3-weekly CHOP Chemotherapy with or without Etoposide for the Treatment of Elderly Patients with Aggressive Lymphomas: results of the NHL-B2 trial of the DSHNHL.** *Blood* 2004, **104**(3):634–641.

33. Pfreundschuh M, Truemper L, Kloess M, Schmits R, Feller A, et al: **2-weekly or 3-weekly CHOP Chemotherapy with or without Etoposide for the Treatment of Young Patients with Good Prognosis (Normal LDH) Aggressive Lymphomas: results of the NHL-B1 trial of the DSHNHL.** *Blood* 2004, **104**(3):626–633.

34. Diehl V, Franklin J, Pfreundschuh M, Lathan B, Paulus U, et al: **Standard and Increased-Dose BEACOPP Chemotherapy Compared with COPP-ABVD for Advanced Hodgkin's Disease.** *N Engl J Med* 2003, **348**.

35. Pfreundschuh M, Schubert J, Ziepert M, Schmits R, et al: **Six versus eight cycles of bi-weekly CHOP-14 with or without rituximab in elderly patients with aggressive CD20+ B-cell lymphomas: a randomised controlled trial (RICOVER-60).** *Lancet Oncol* 2008, **9**(2):105–116.

36. Pfreundschuh M, Zwick C, Zeynalova S, Dührsen U, Pflüger KH, et al: **Dose-escalated CHOEP for the treatment of young patients with aggressive non-Hodgkin's lymphoma: II. Results of the randomized high-CHOEP trial of the German High-Grade Non-Hodgkin's Lymphoma Study Group (DSHNHL).** *Ann Oncol* 2008, **19**:545–552.

37. Trümper L, Zwick C, Ziepert M, Hohloch K, Schmits R: **German High-Grade Non-Hodgkin's Lymphoma Study Group (DSHNHL). Dose-escalated CHOEP for the treatment of young patients with aggressive non-Hodgkin's lymphoma: I. A randomized dose escalation and feasibility study with bi- and tri-weekly regimens.** *Ann Oncol* 2008, **19**(3):538–544.

38. Johnston E, Crawford C, Blackwell S, Bjurstrom T, Lockbaum P, et al: **Randomized, dose-escalation study of sd/01 compared with daily filgrastim in patients receiving chemotherapy.** *J. Clin. Oncol.* 2000, **18(13)**:2522–2528.

39. Grigg A, Roberts A, Raunow H, Houghton S, Layton J, et al: **Optimizing Dose and Scheduling of Filgrastim (Granulocyte Colony - Stimulating Factor) for Mobilization and Collection of Peripheral Blood Progenitor Cells in Normal Volunteers**. *Blood* 1995, **86**(12):4437–4445.

40. van der Auwera P, Platzer E, Xu Z, Schulz R, Feugeas O, et al: **Pharmacodynamics and pharmacokinetics of single doses of subcutaneous pegylated human g-csf mutant (Ro 25-8315) in healthy volunteers: comparison with single and multiple daily doses of filgrastim**. *Am. J. Hematol.* 2001, **66(4)**:245–251.

41. Borleffs J, Bosschaert M, Vrehen H, Schneider M, van Strijp J, et al: **Effect of escalating doses of recombinant human granulocyte colony-stimulating factor (filgrastim) on circulating neutrophils in healthy subjects**. *Clin. Ther.* 1998, **20(4)**:722–736.

42. Chatta G, Price T, Allen R, Dale D: **Effects of in vivo recombinant methionyl human granulocyte colony-stimulating factor on the neutrophil response and peripheral blood colony-forming cells in healthy young and elderly adult volunteers**. *Blood* 1994, **84(9)**:2923–2929.

43. Varki R, Pequignot E, Leavitt M, Ferber A, Kraft W: **A glycosylated recombinant human granulocyte colony stimulating factor produced in a novel protein production system (AVI-014) in healthy subjects: a first-in human, single dose, controlled study**. *Blood* 2009, **9(2)**.

44. Bensinger W, Price T, Dale D, Appelbaum F, Clift R, et al: **The effects of daily recombinant human granulocyte colony-stimulating factor administration on normal granulocyte donors undergoing leukapheresis .** *Blood* 1993, **81**:1883–1888.

45. Wang B, Ludden T, Cheung E, Schwab G, Roskos L: **Population pharmacokinetic-pharmacodynamic modeling of filgrastim (r-metHuG- CSF) in healthy volunteers**. *J. Pharmacokinet. Pharmacodyn.* 2001, **28**:321–342.

46. Roskos L, Lum P, Lockbaum P, Schwab G, Yang B: **Pharmacokinetic / pharmacodynamic modeling of pegfilgrastim in healthy subjects**. *J. Clin. Pharmacol.* 2006, **46**:747–757.

47. Holmes F, Jones S, O'Shaughnessy J, Vukelja S, George T, et al: **Comparable efficacy and safety profiles of once-per-cycle pegfilgrastim and daily injection filgrastim in chemotherapy-induced neutropenia: a multicenter dose-finding study in women with breast cancer**. *Ann. Oncol.* 2002, **13**:903–909.

48. Green M, Koelb H, Baselga J, Galid A, Guillem V, et al: **A randomized double-blind multicenter phase III study of fixed-dose single-administration pegfilgrastim versus daily filgrastim in patients receiving myelosuppressive chemotherapy. DOI: 10.1093**. *Ann. Oncol.* 2003, **14**:29–35.

49. Zamboni W, Blackwell S: **Pharmacokinetics of pegfilgrastim.** *Pharmacotherapy* 2003, **23**:9S–14S.

50. Yowell S, Blackwell S: **Novel effects with polyethylene glycol modified pharmaceuticals.** *Cancer Treat Rev* 2002, **28 Suppl A**:3–6.

51. George S, Yunus F, Case D, Yang B, Hackett J, et al: **Fixed-dose pegfilgrastim is safe and allows neutrophil recovery in patients with non-hodgkins lymphoma.** *J. Clin. Oncol.* 2003, **44(10)**:1691–1696.

52. Mey U, Maier A, Schmidt-Wolf I, Ziske C, Forstbauer H, et al: **Pegfilgrastim as hematopoietic support for dose-dense chemoimmunotherapy with R-CHOP-14 as first-line therapy in elderly patients with diffuse large B cell lymphoma. (DOI 10.1007/s00520-006-0201-z)**. *Support Care Cancer* 2007, **15**:877–884.

53. Brusamolino E, Rusconi C, Montalbetti L, Gargantini L, Uziel L, et al: **Dose-dense R-CHOP-14 supported by pegfilgrastim in patients with diffuse large B-cell lymphoma: a phase II study of feasibility and toxicity.** *Haematologica* 2006, **91**:496–502.

54. Vose J, Crump M, Lazarus H, Emmanouilides C, Schenkein D, et al: **Randomized, Multicenter, Open-Label Study of Pegfilgrastim Compared With Daily Filgrastim After Chemotherapy for Lymphoma**. *Journal of Clinical Oncology* 2003, **21(3)**:514–519.

55. Zwick C, Zeynalova S, Pöschel V, Nickenig C, et al: **Randomized comparison of pegfilgrastim day 4 versus day 2 for the prevention of chemotherapy-induced leukocytopenia.** *Ann Oncology* 2011, **22**:1872–1877.

56. Wiczling P, Lowe P, Pigeolet E, dicke FL, Balser S, Krzyzanski W: **Population Pharmacokinetic Modelling of Filgrastim in Healthy Adults following Intravenous and Subcutaneous Administrations.** *Clin Pharmacokinet* 2009, **48**(12):817–826.

57. Rutherford C, Schneider T, Dempsey H, Kirn D, Brugnara C, et al: **Efficacy of Different Dosing Regimens for Recombinant Human Erythropoietin in a Simulated Perisurgical Setting: The Importance of Iron Availability in Optimizing Response.** *Am J Med* 1994, **96**:139–145.

58. Sieber M, Bredenfeld H, Josting A, Reineke T, Rueffer U, et al: **14-day variant of the bleomycin, etoposide, doxorubicin, cyclophosphamide, vincristine, procarbazine, and prednisone regimen in advanced-stage Hodgkin's lymphoma: results of a pilot study of the German Hodgkin's Lymphoma Study Group.** *J Clin Oncol* 2003, **21**(9):1734–1739.
